# Supplementary figures and images for: Acidosis attenuates the hypoxic stabilization of HIF-1α by activating lysosomal degradation
Source: J Cell Biol. 2025 Jun 24;224(8):e202409103. doi: 10.1083/jcb.202409103 (PMC12187095; doi:10.1083/jcb.202409103)

Figure 2

A

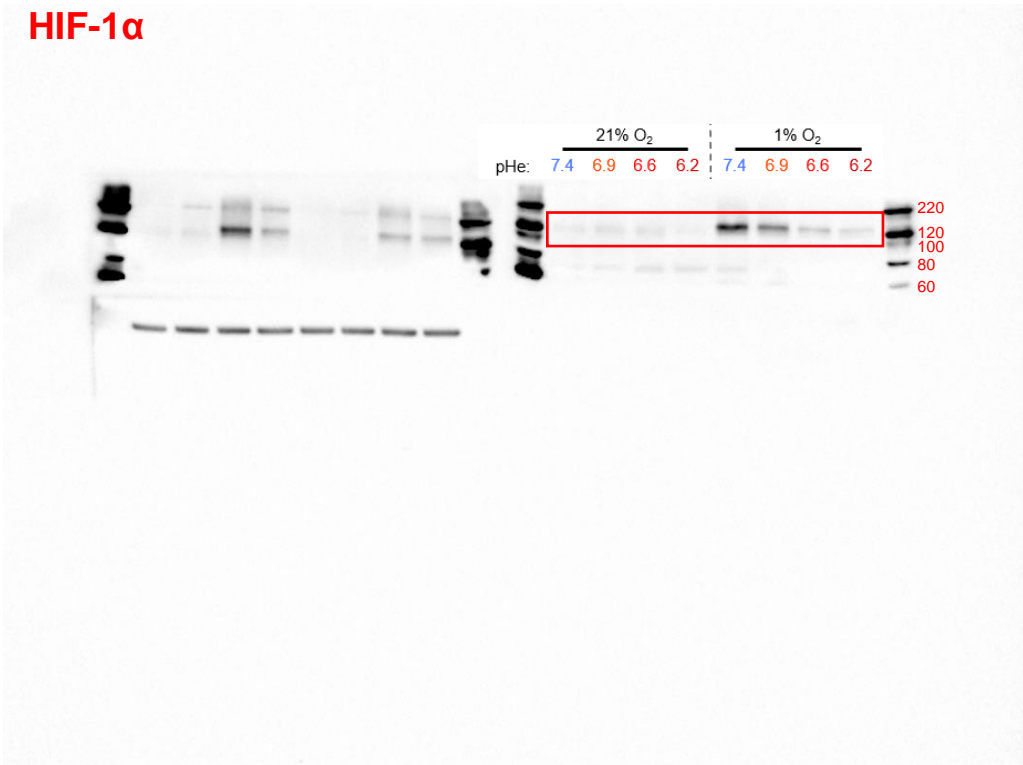

A

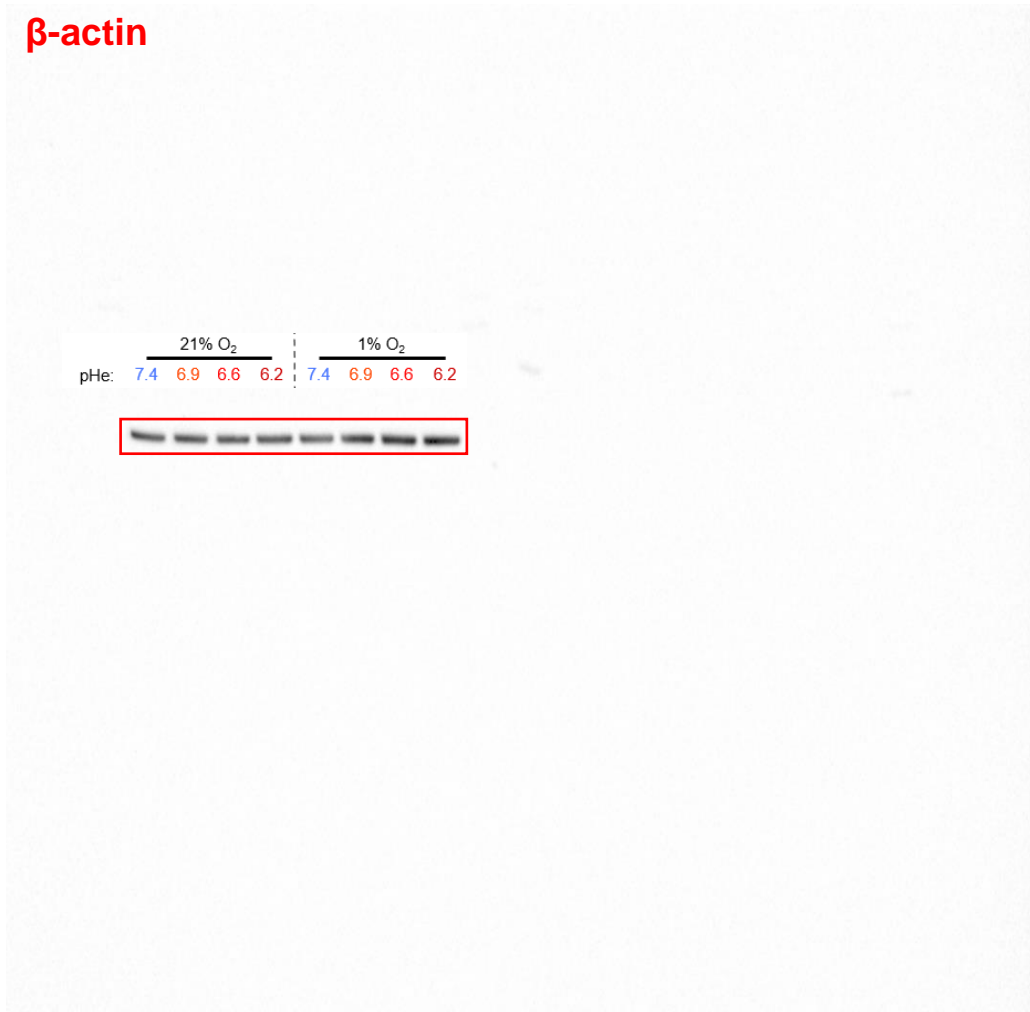

B

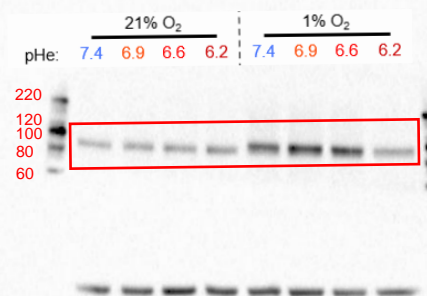

Figure 2

B

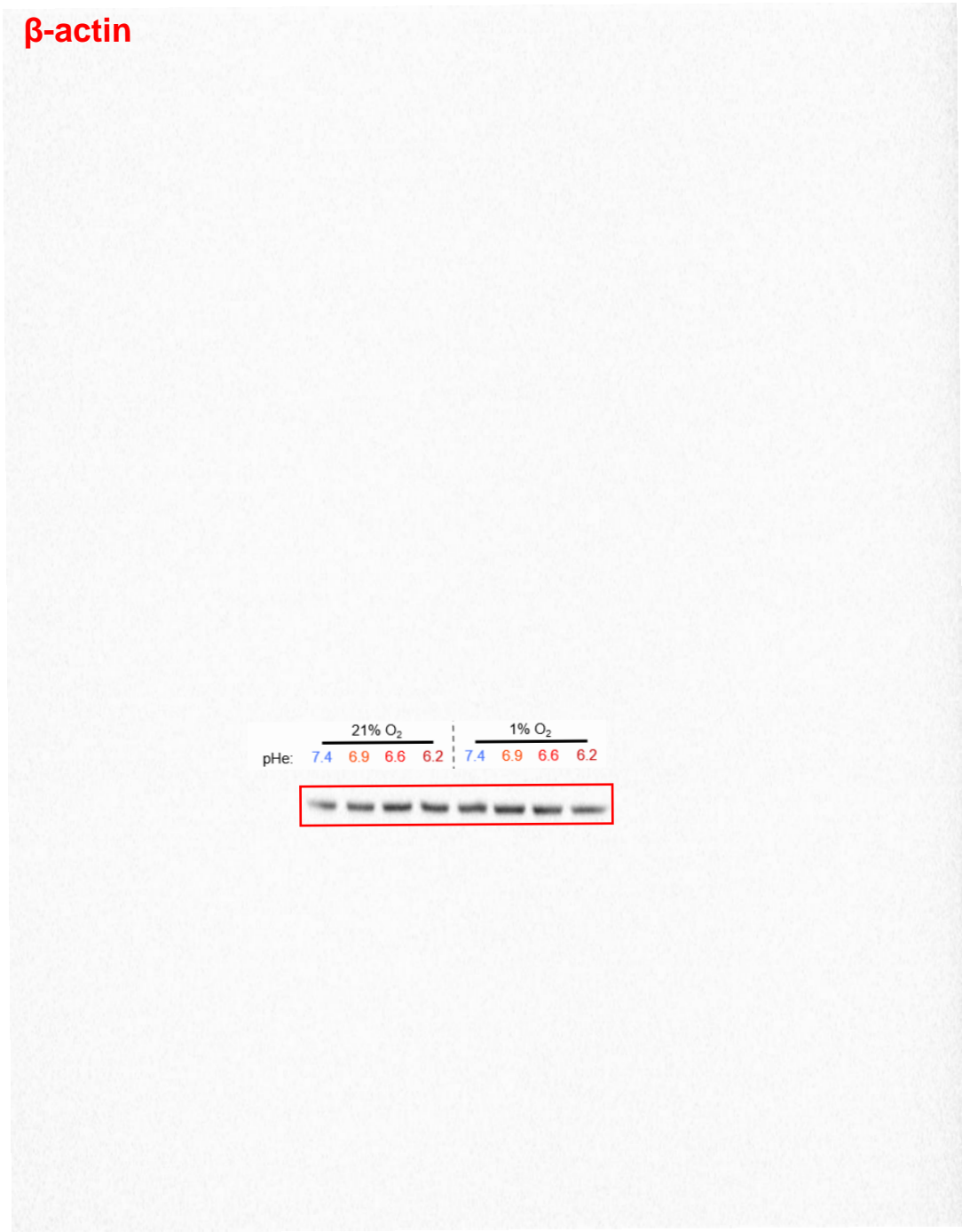

Figure 2

C

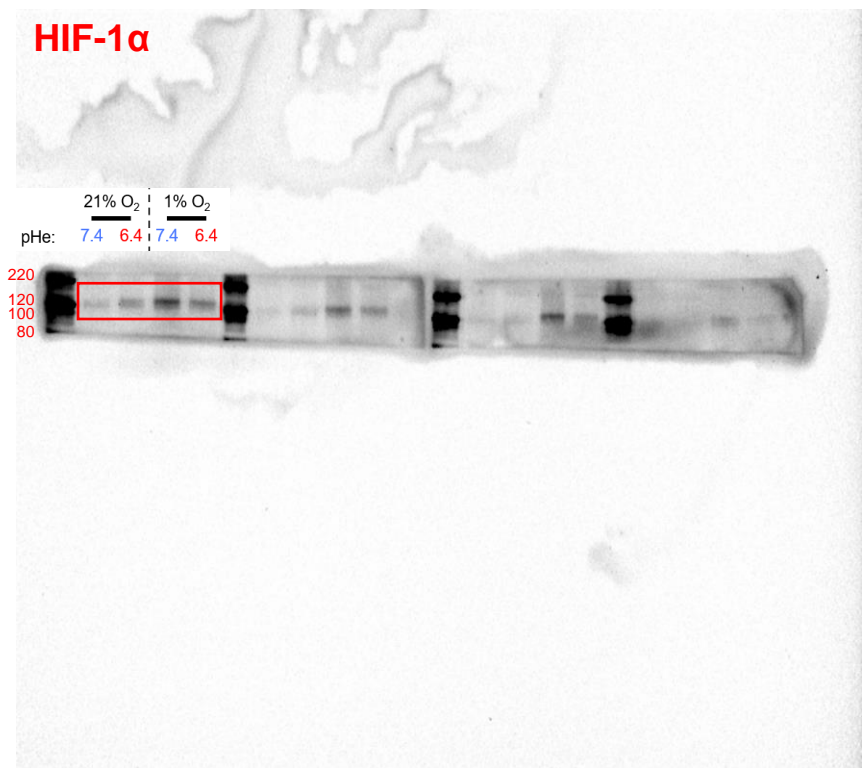

C

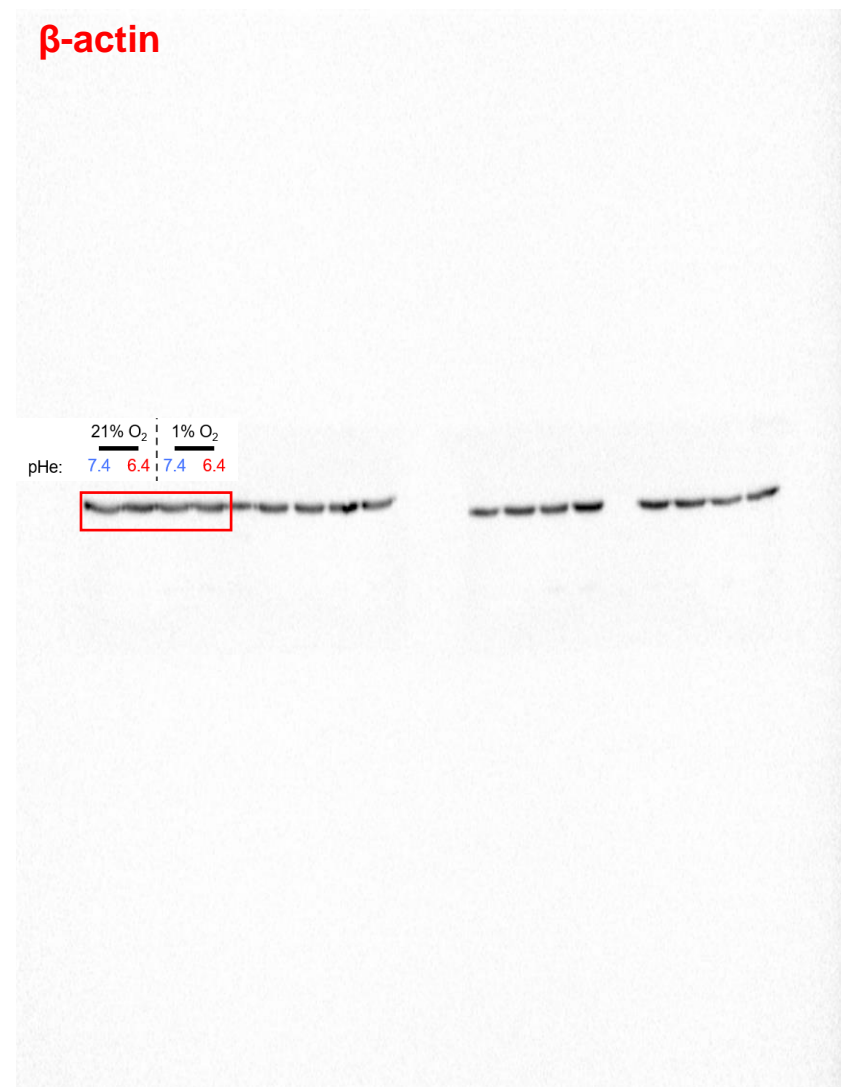

Figure 2

D

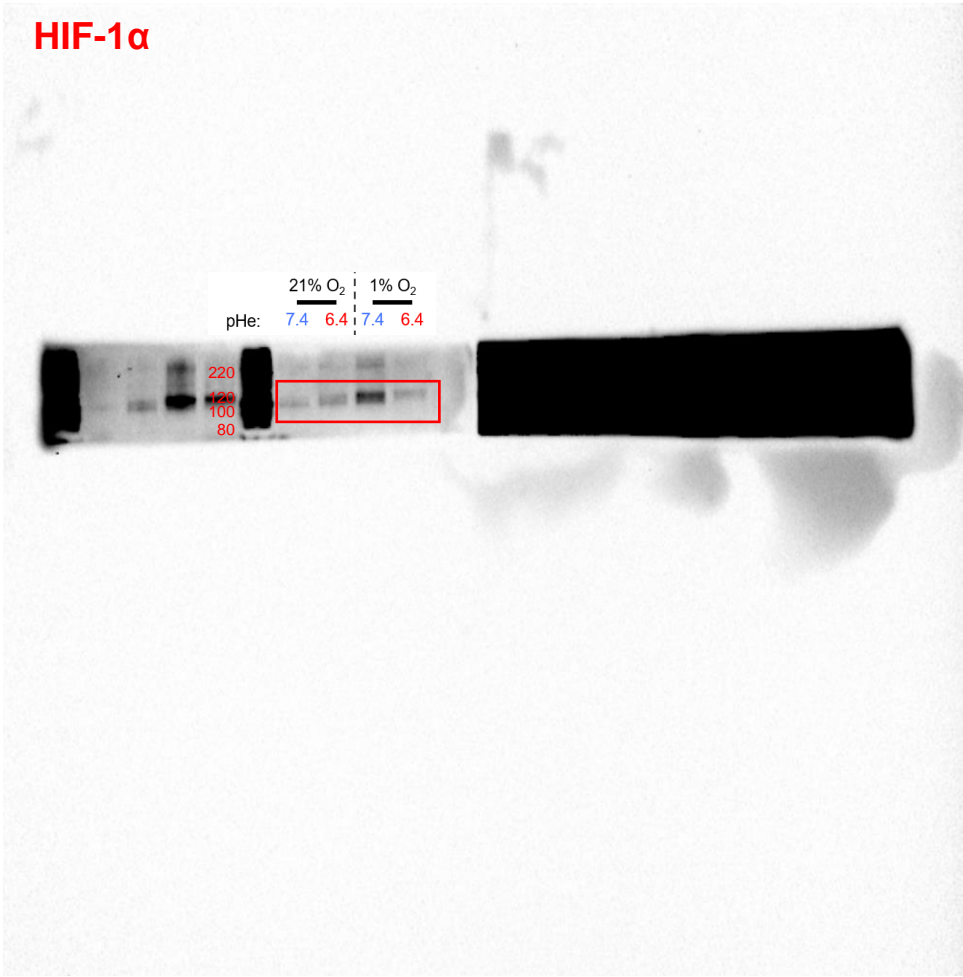

D

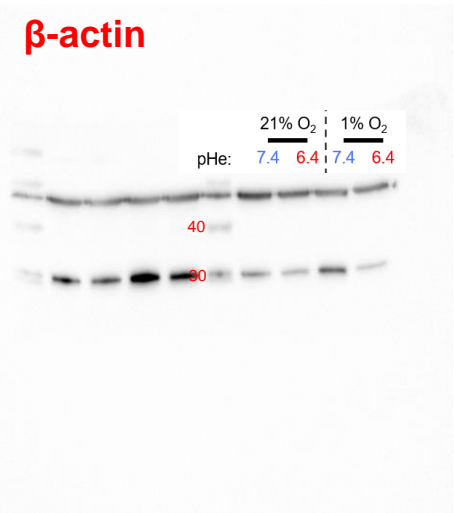

Figure 2

E

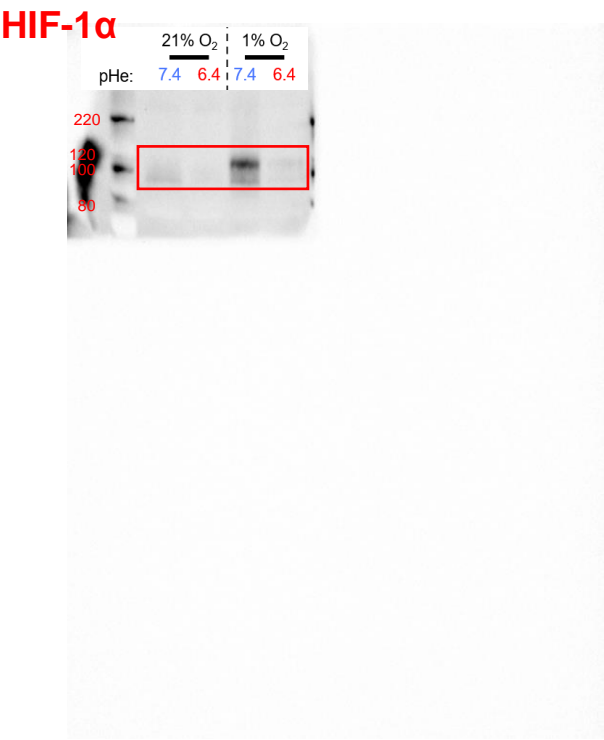

E

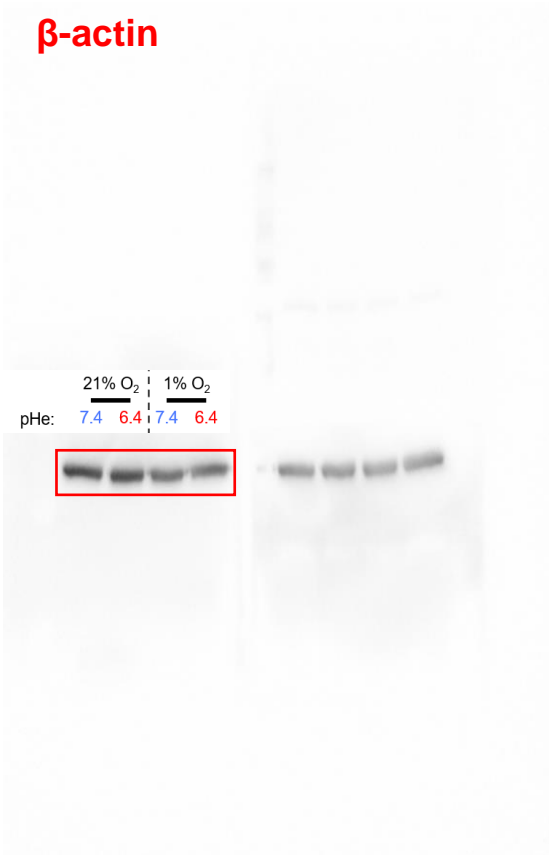

Figure 2

F

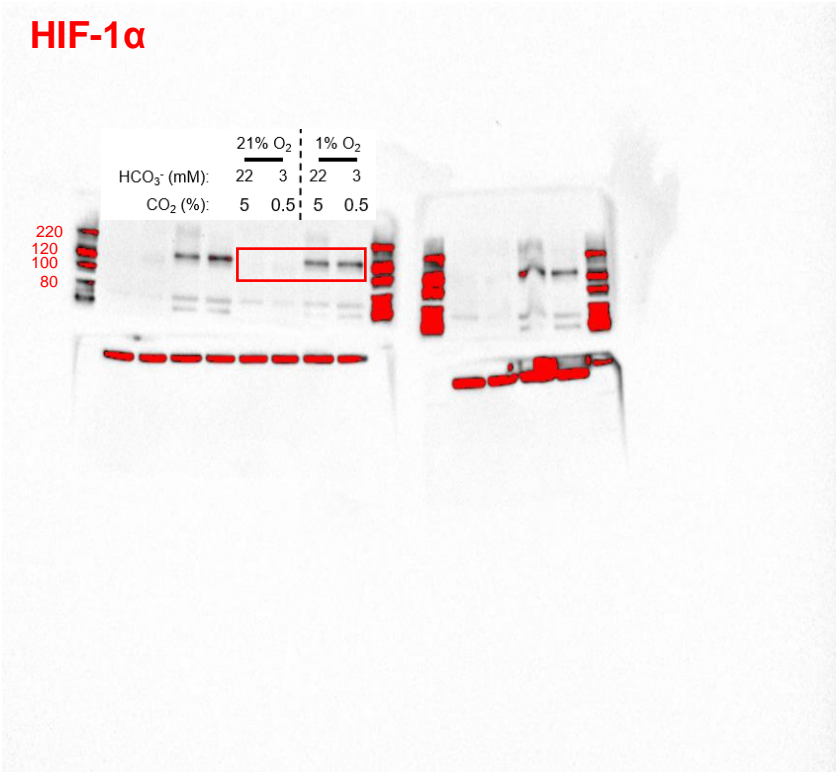

F

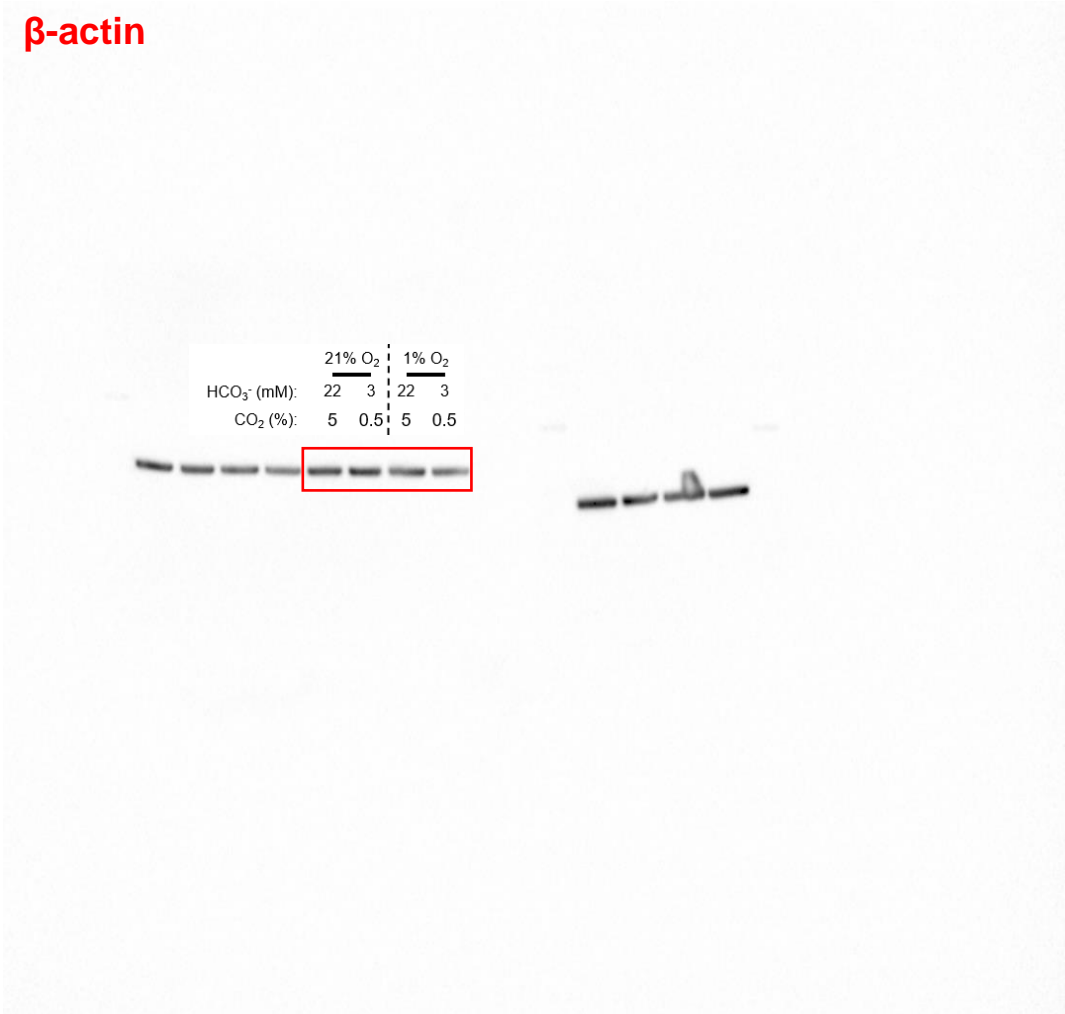

Figure 2

G

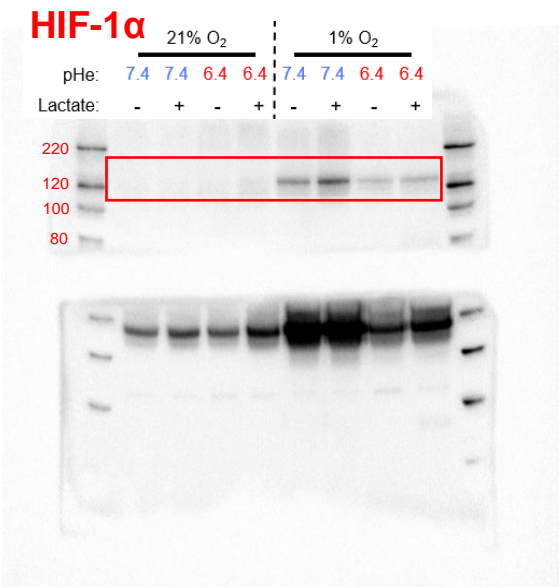

G

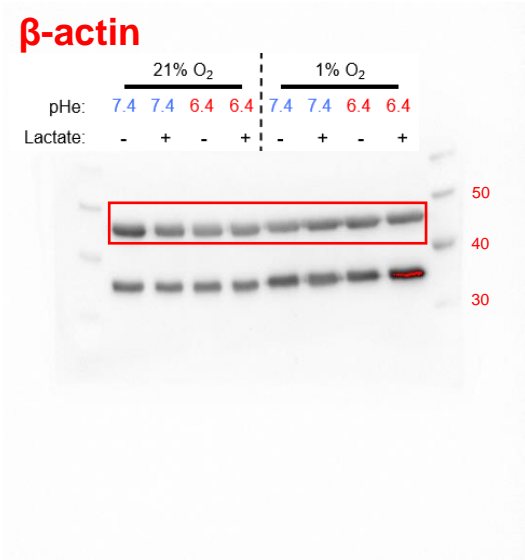

Supplement: SourceData F2 — is the source file for Fig. 2. [file jcb_202409103_sourcedataf2.pdf]

Figure 5

B

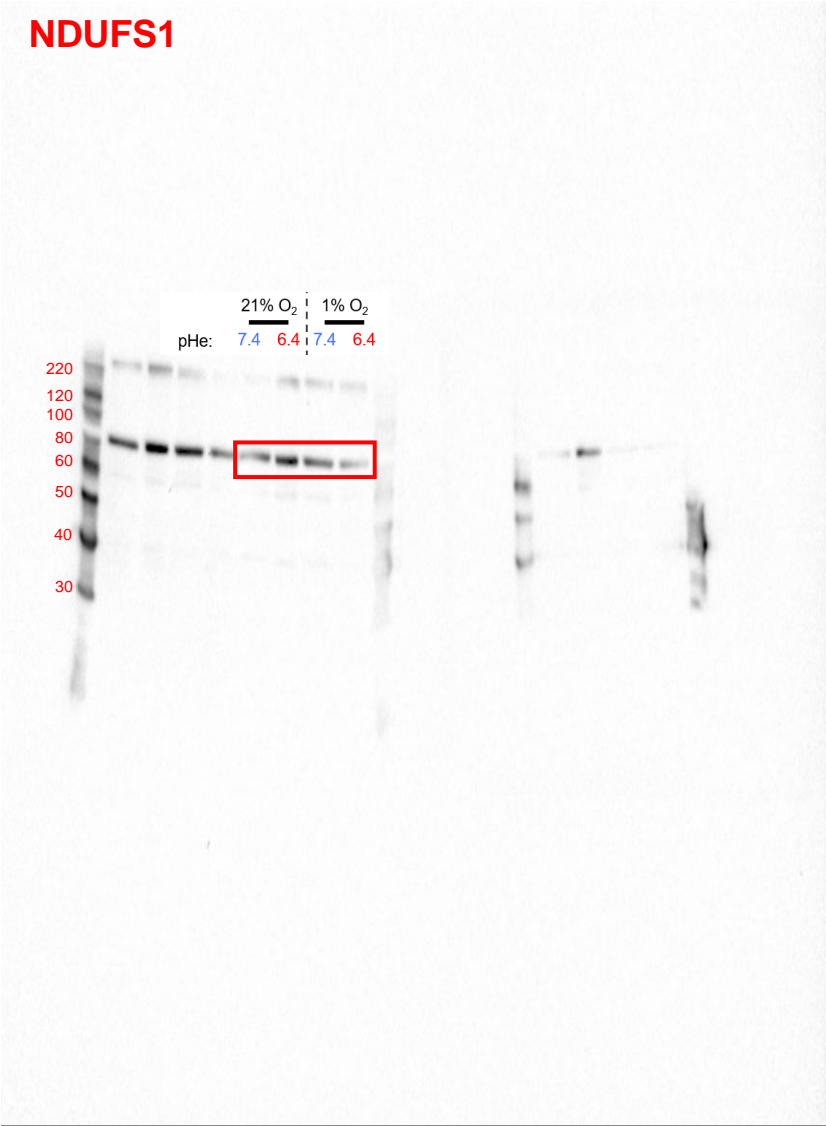

Figure 5

B

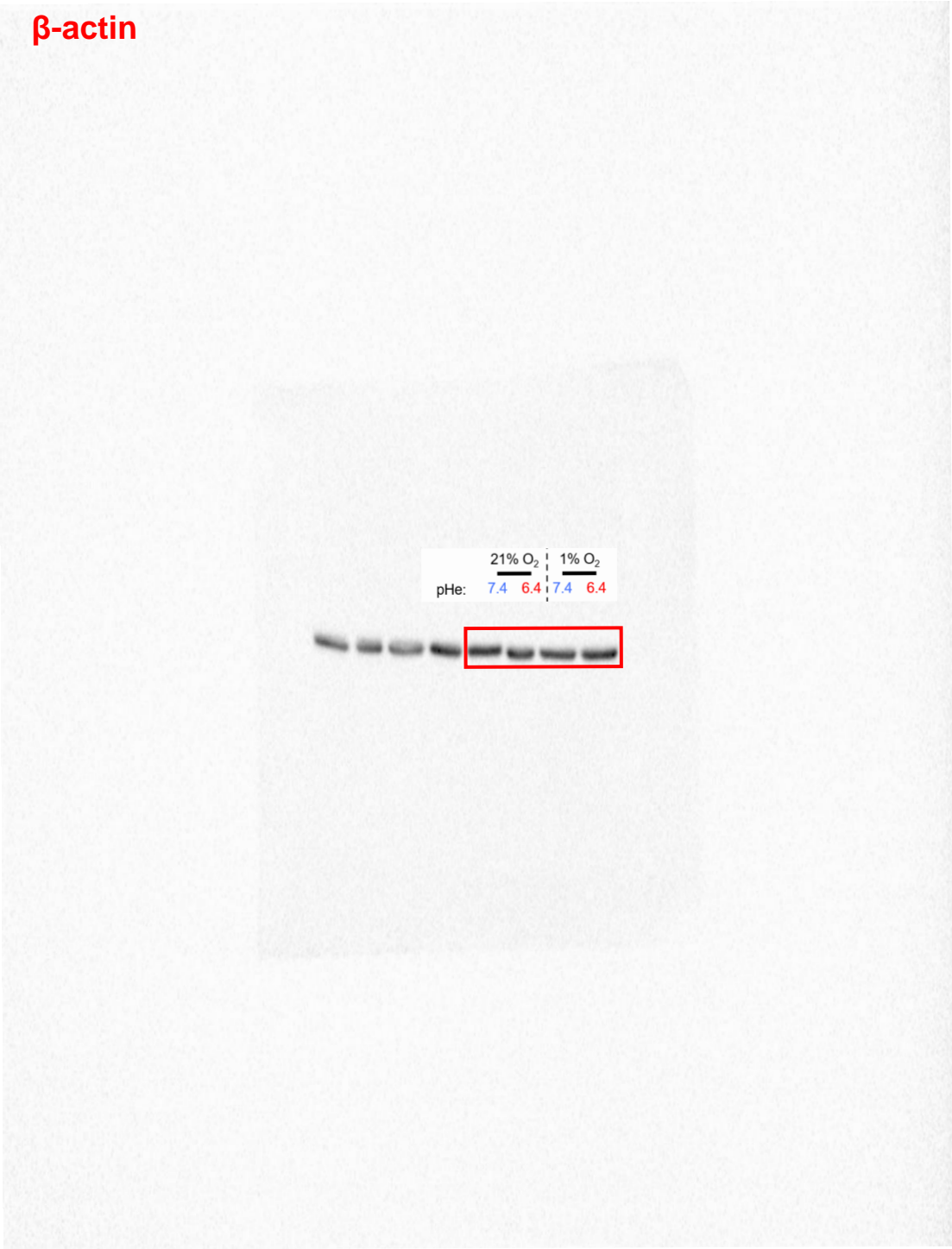

Supplement: SourceData F5 — is the source file for Fig. 5. [file jcb_202409103_sourcedataf5.pdf]

Figure 6

A

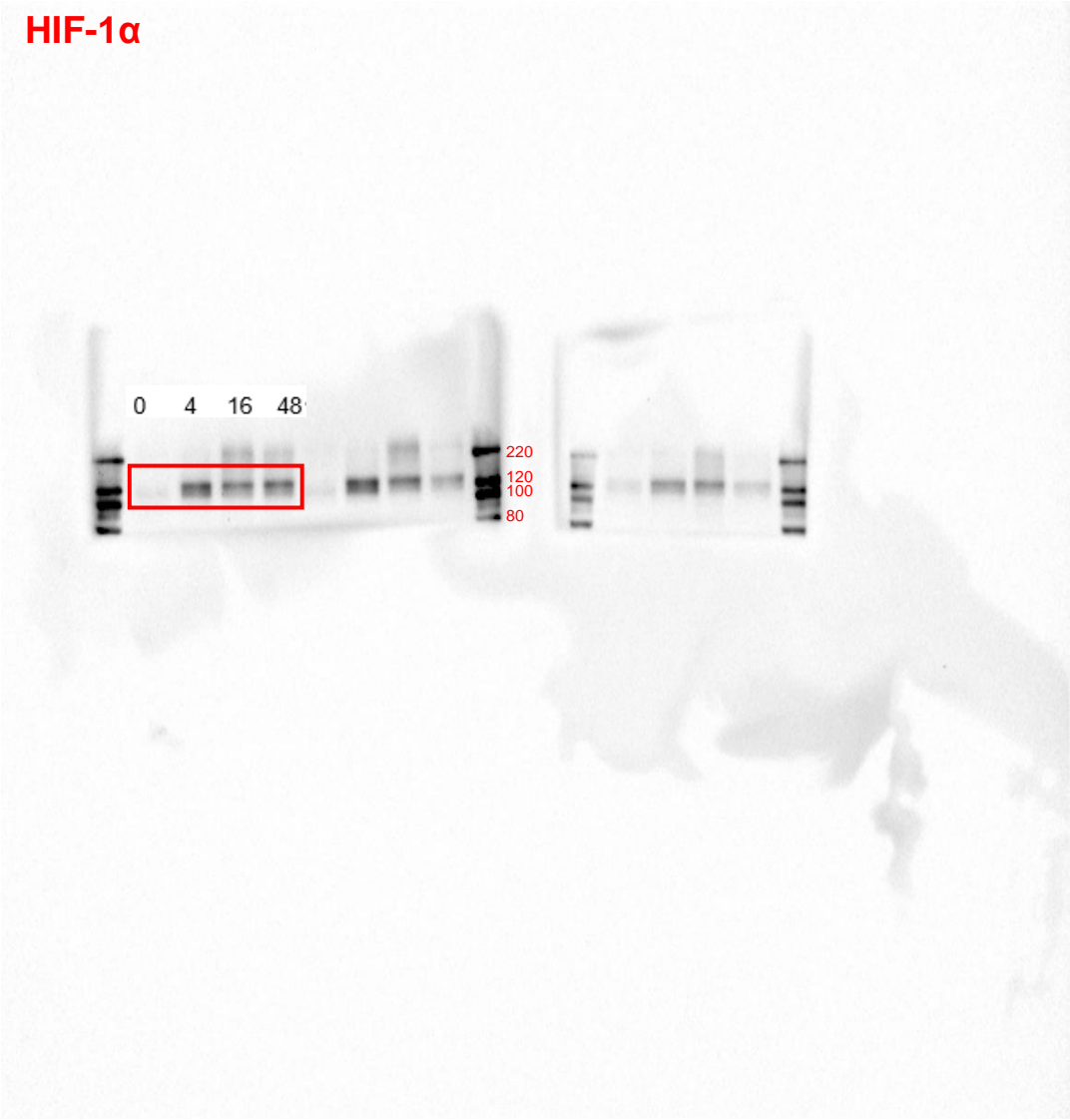

Figure 6

A

$\beta$ -actin

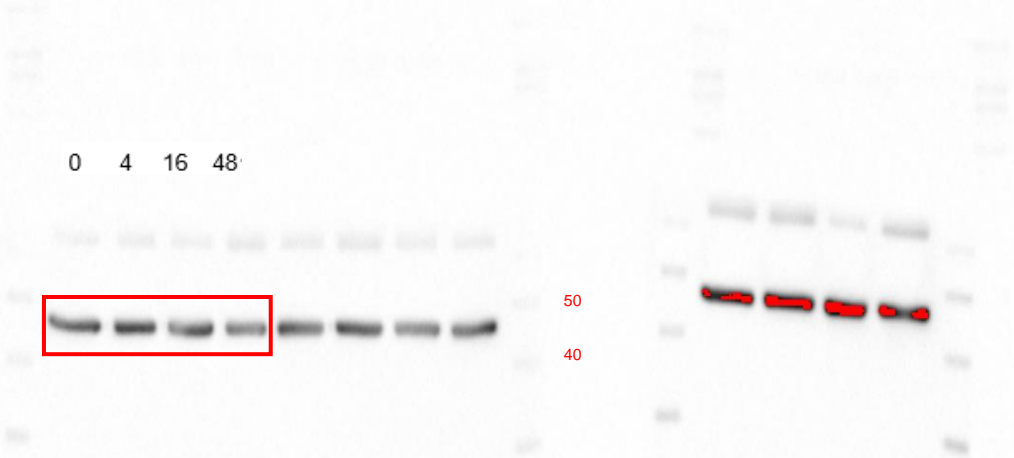

Figure 6

B

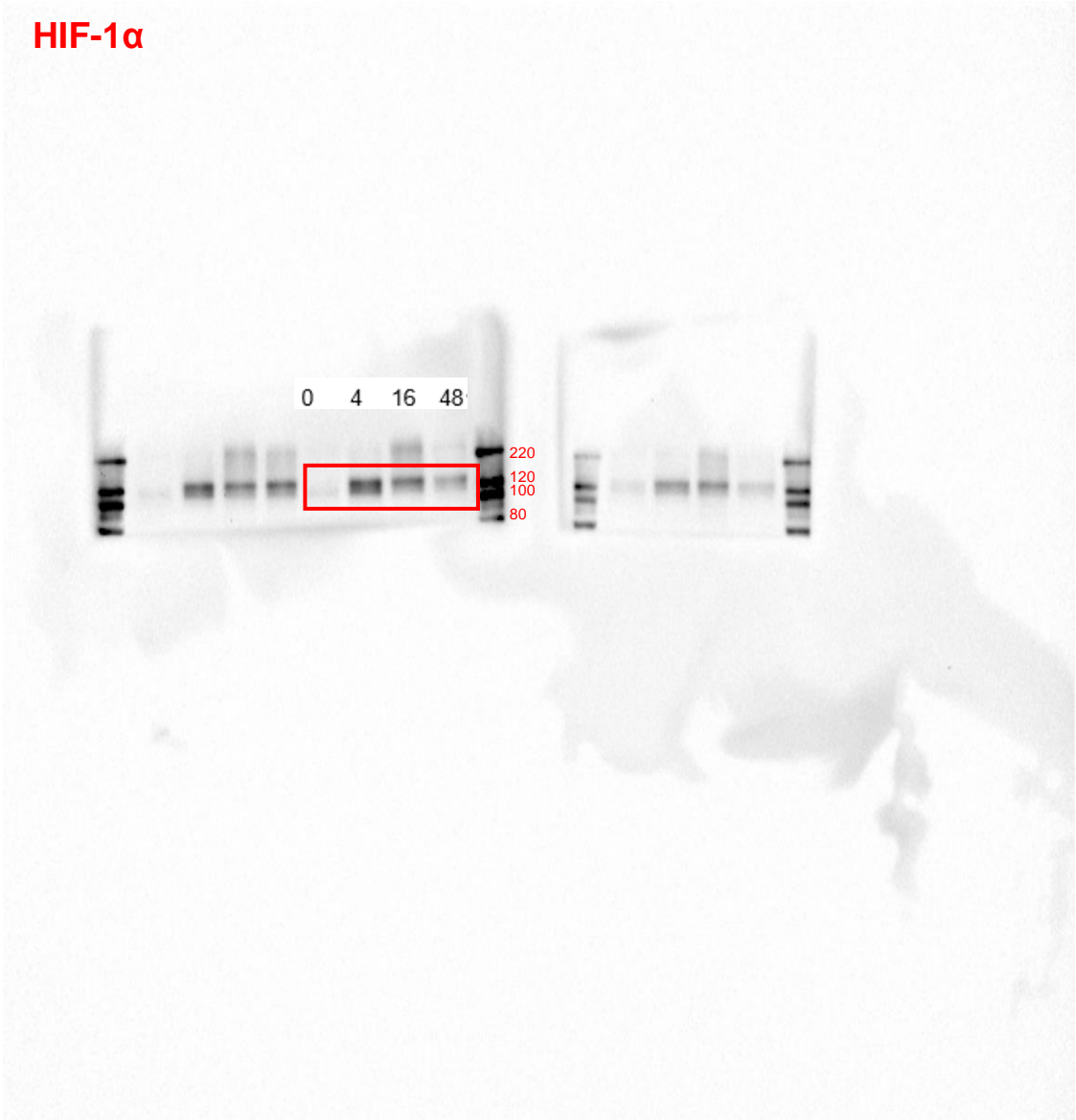

Figure 6

B

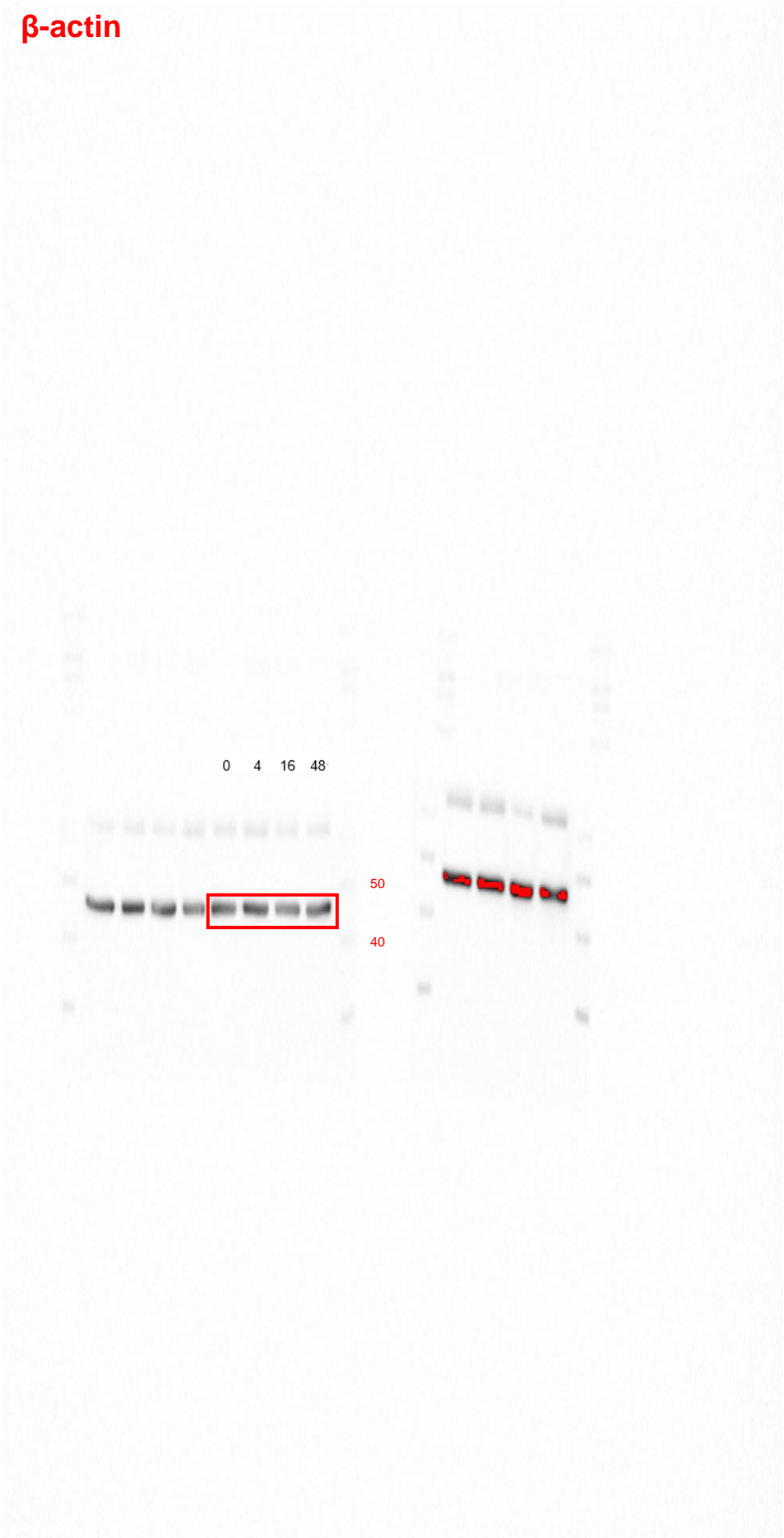

Figure 6

C

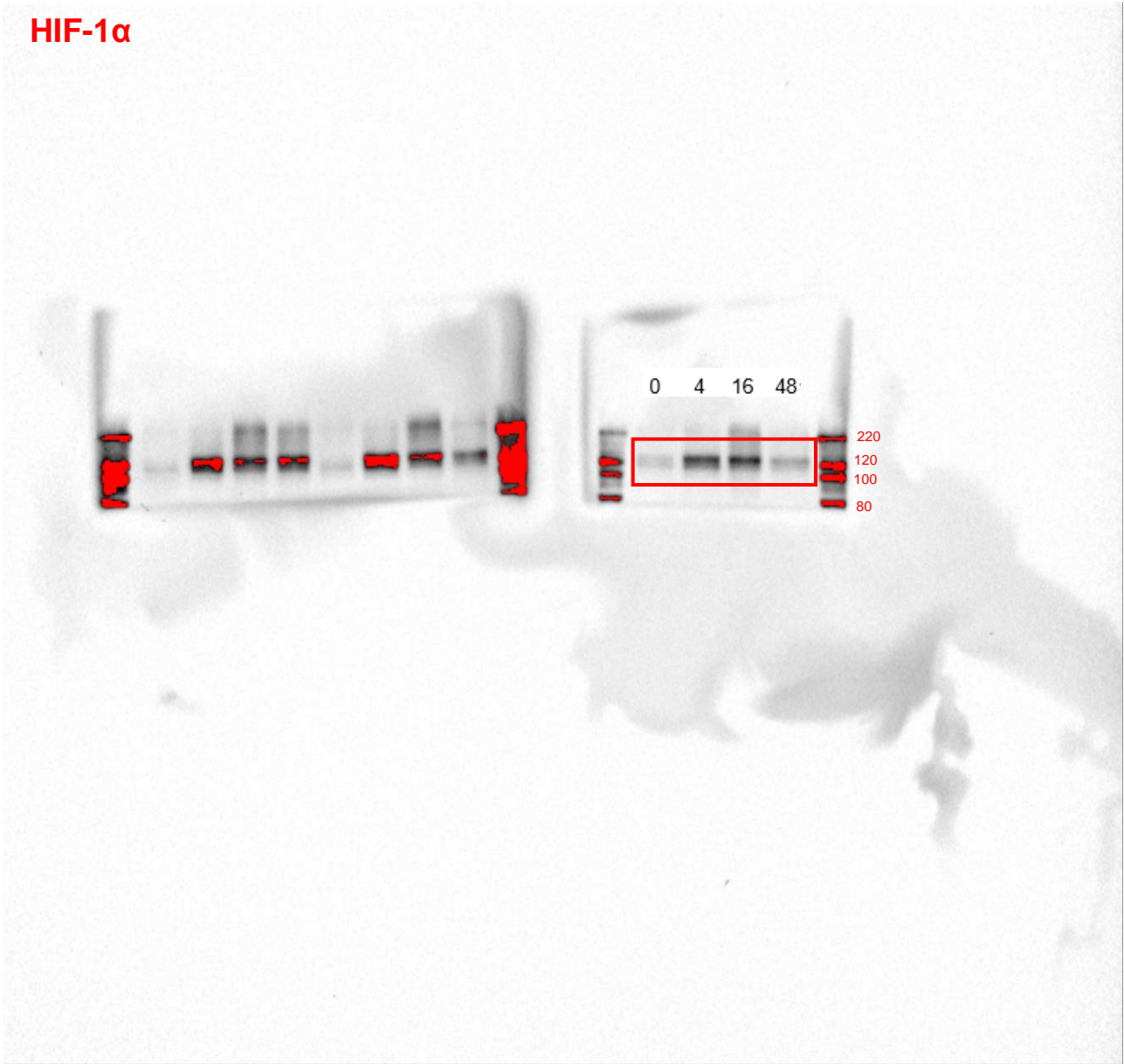

Figure 6

C

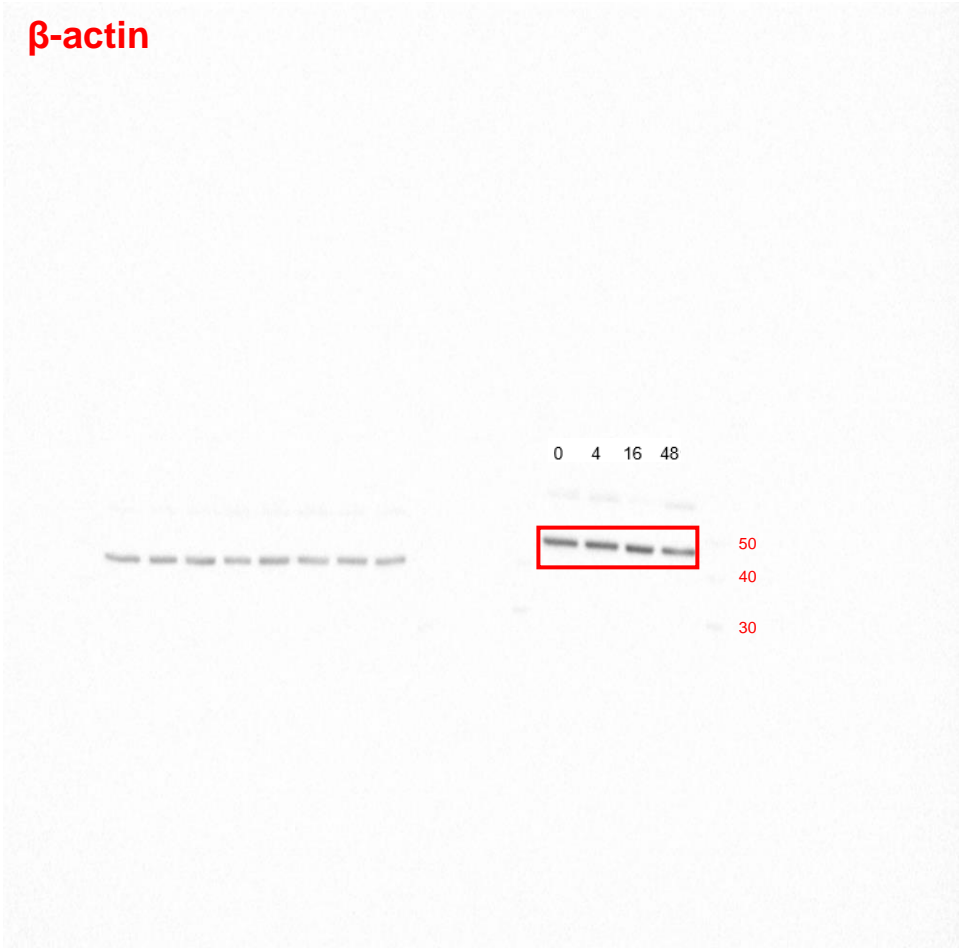

Figure 6

D

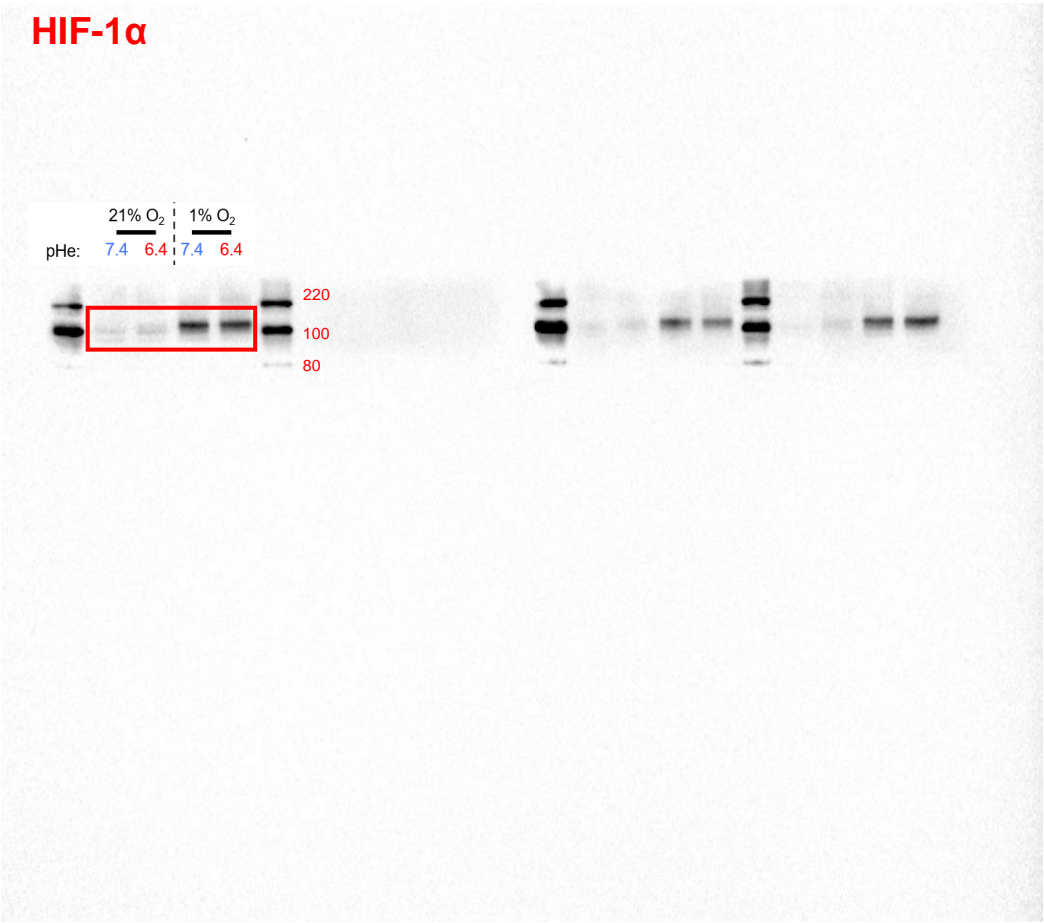

Figure 6

D

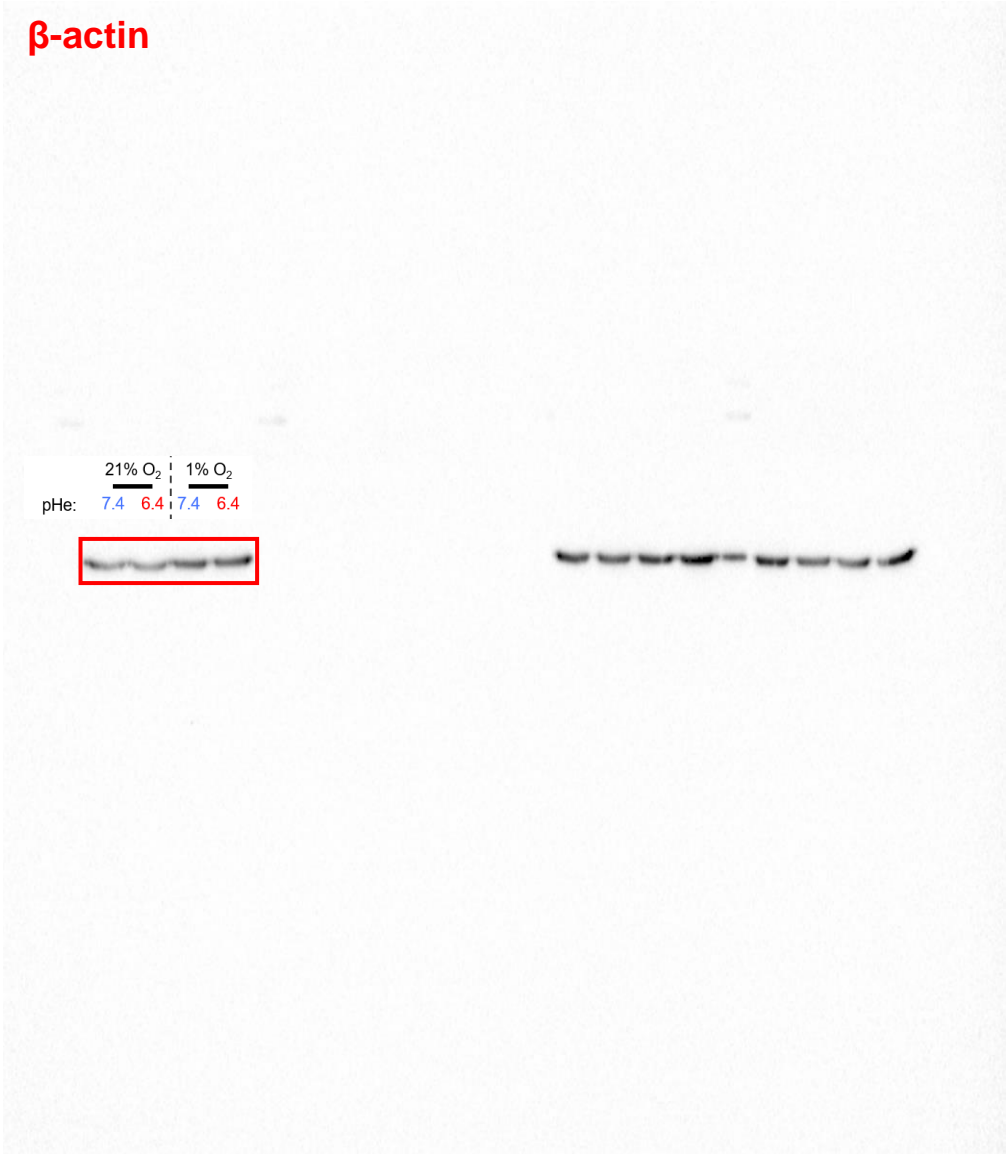

Supplement: SourceData F6 — is the source file for Fig. 6. [file jcb_202409103_sourcedataf6.pdf]

Figure 9

F

Pro-cathepsin B/cathepsin B

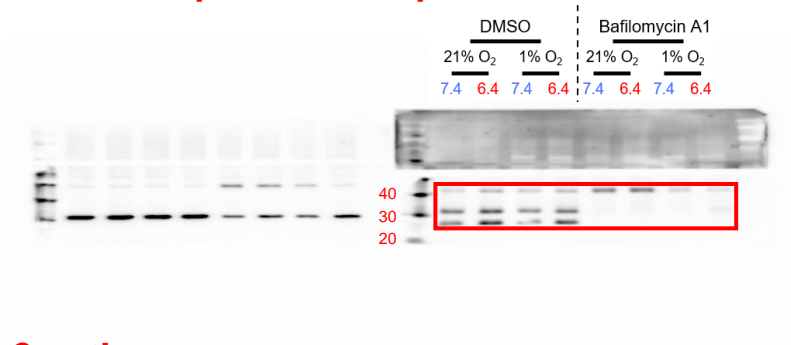

F

β-actin

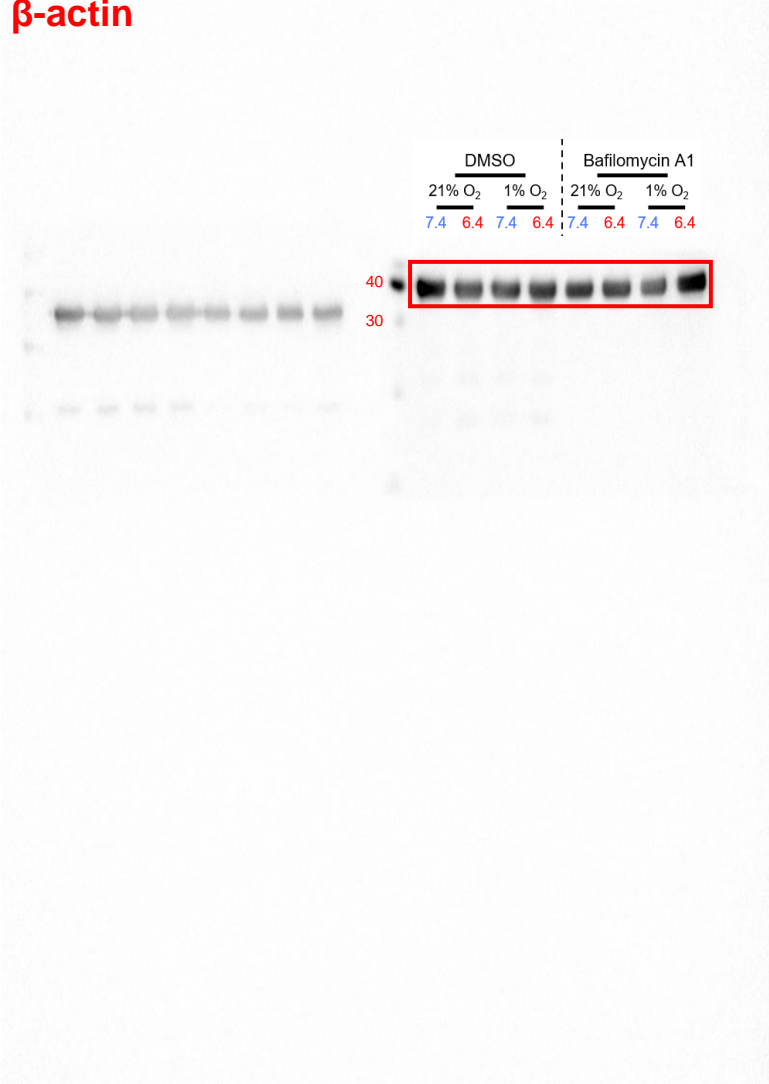

Supplement: SourceData F9 — is the source file for Fig. 9. [file jcb_202409103_sourcedataf9.pdf]
