# Supplementary material for: Acidosis attenuates the hypoxic stabilization of HIF-1α by activating lysosomal degradation
Source: J Cell Biol. 2025 Jun 24;224(8):e202409103. doi: 10.1083/jcb.202409103 (PMC12187095; doi:10.1083/jcb.202409103)

Figure 4

A

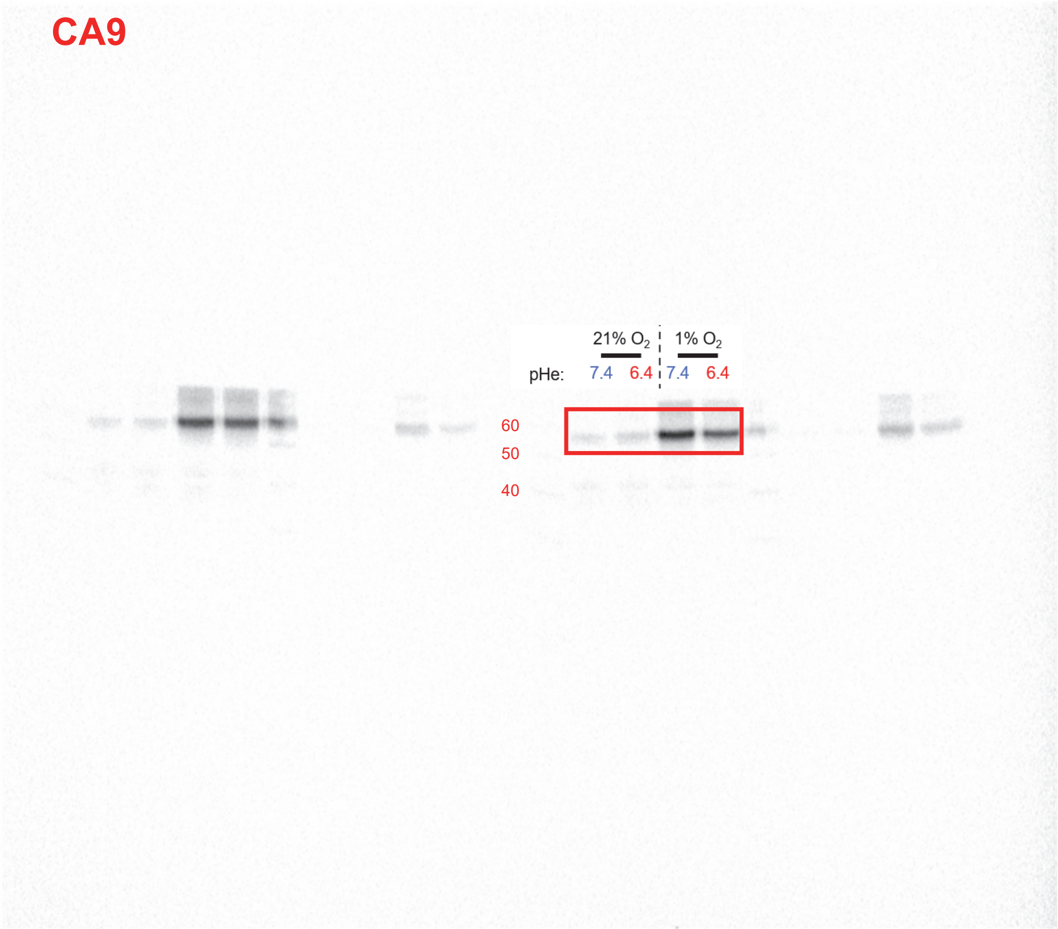

Figure 4

A

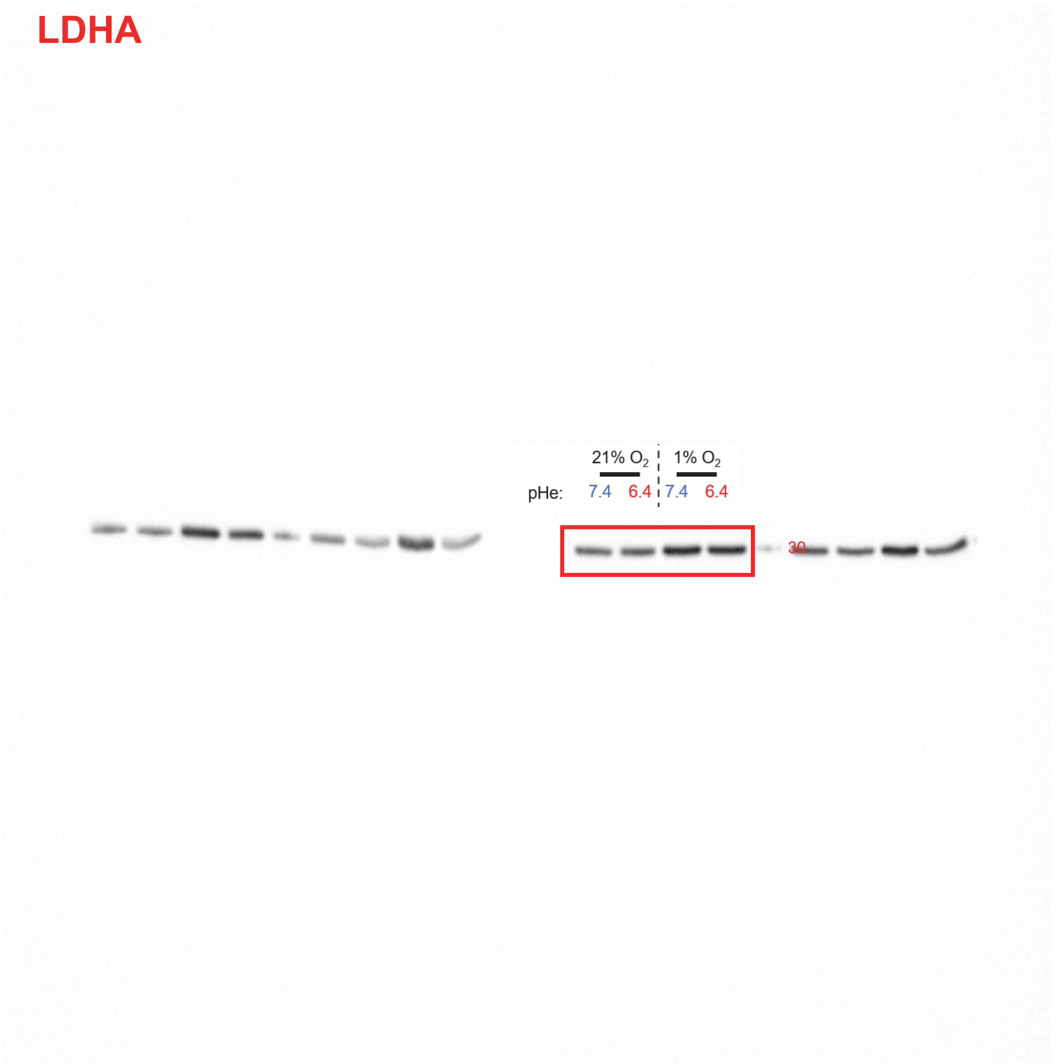

Figure 4

A

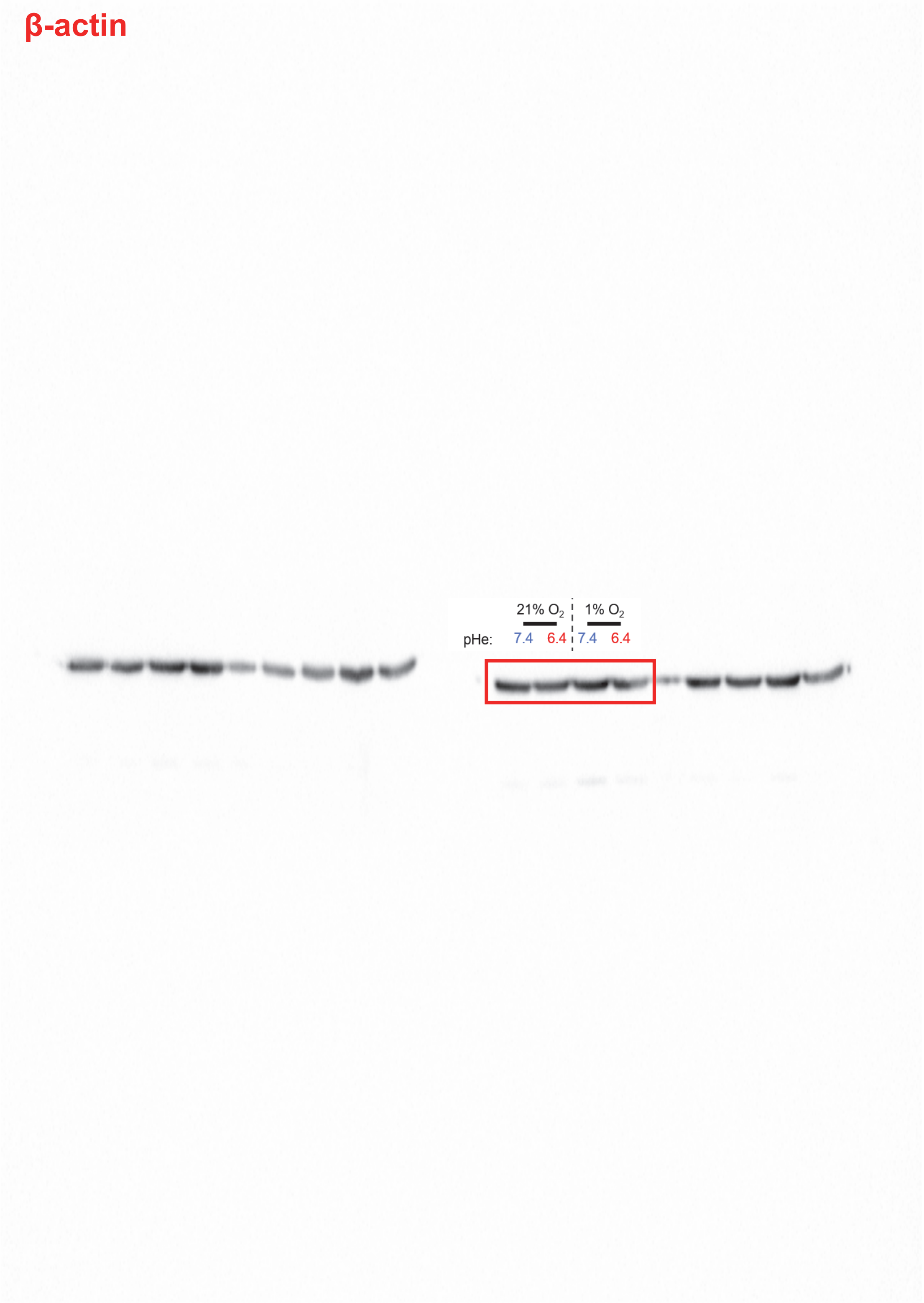

Figure 4

B

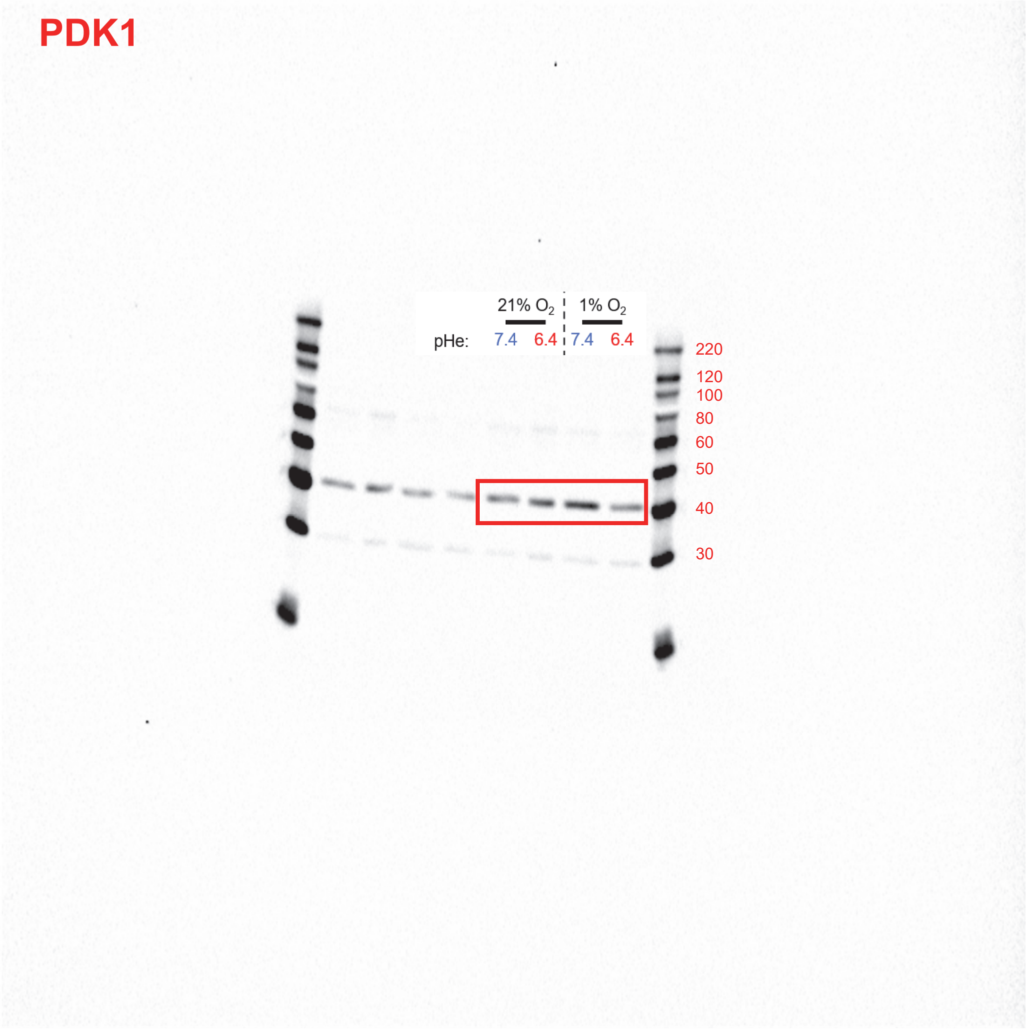

Figure 4

B

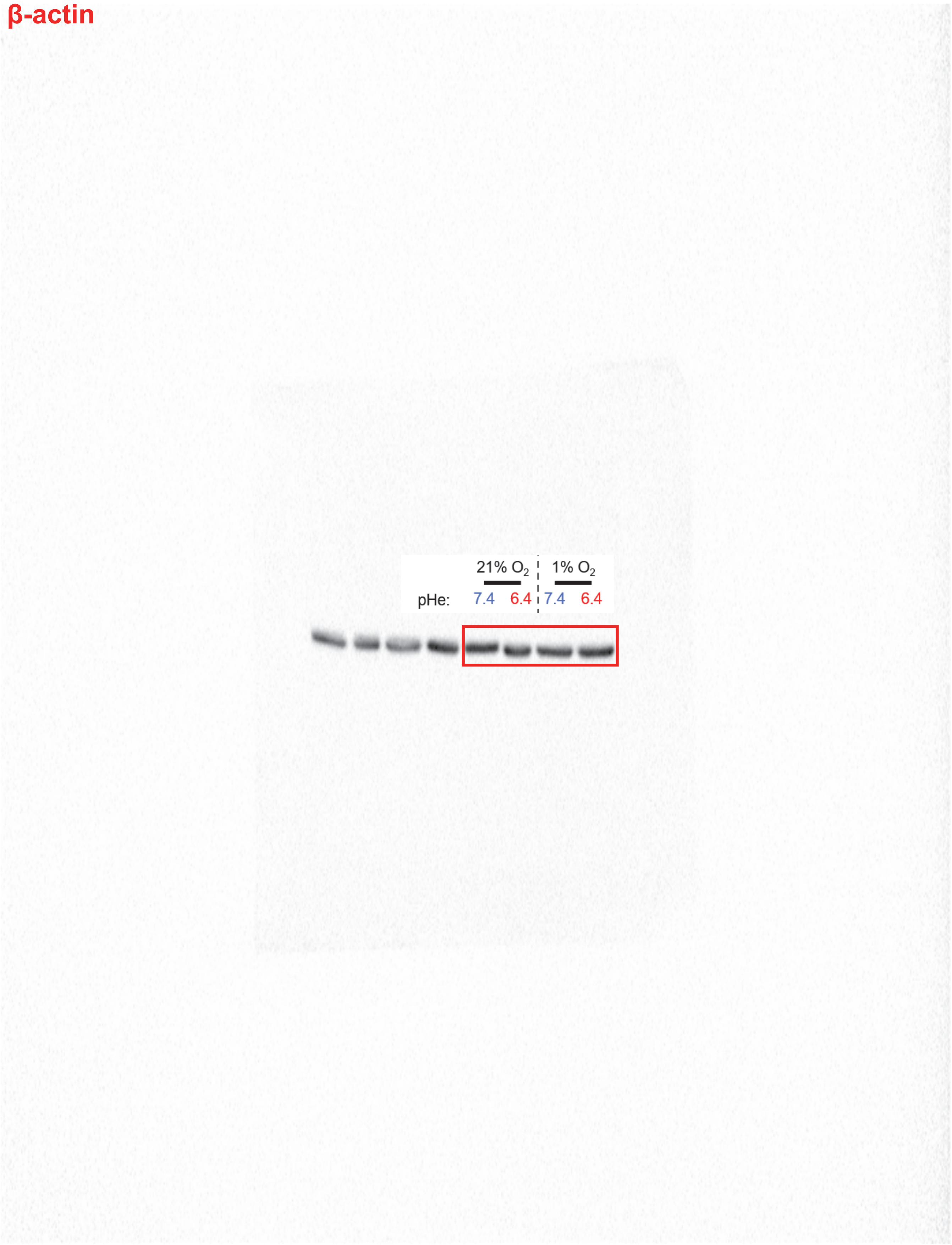

Figure 4

C

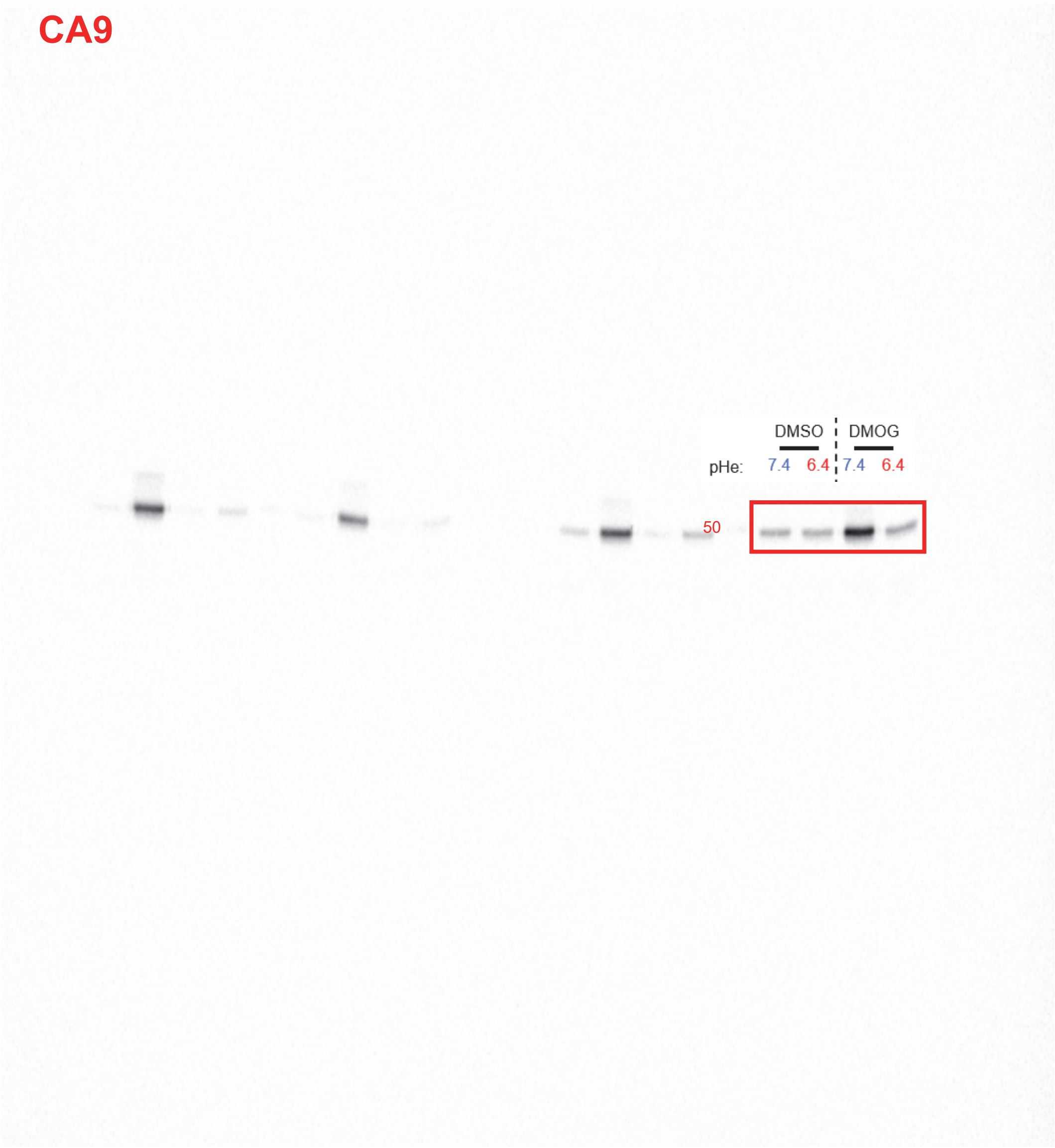

Figure 4

C

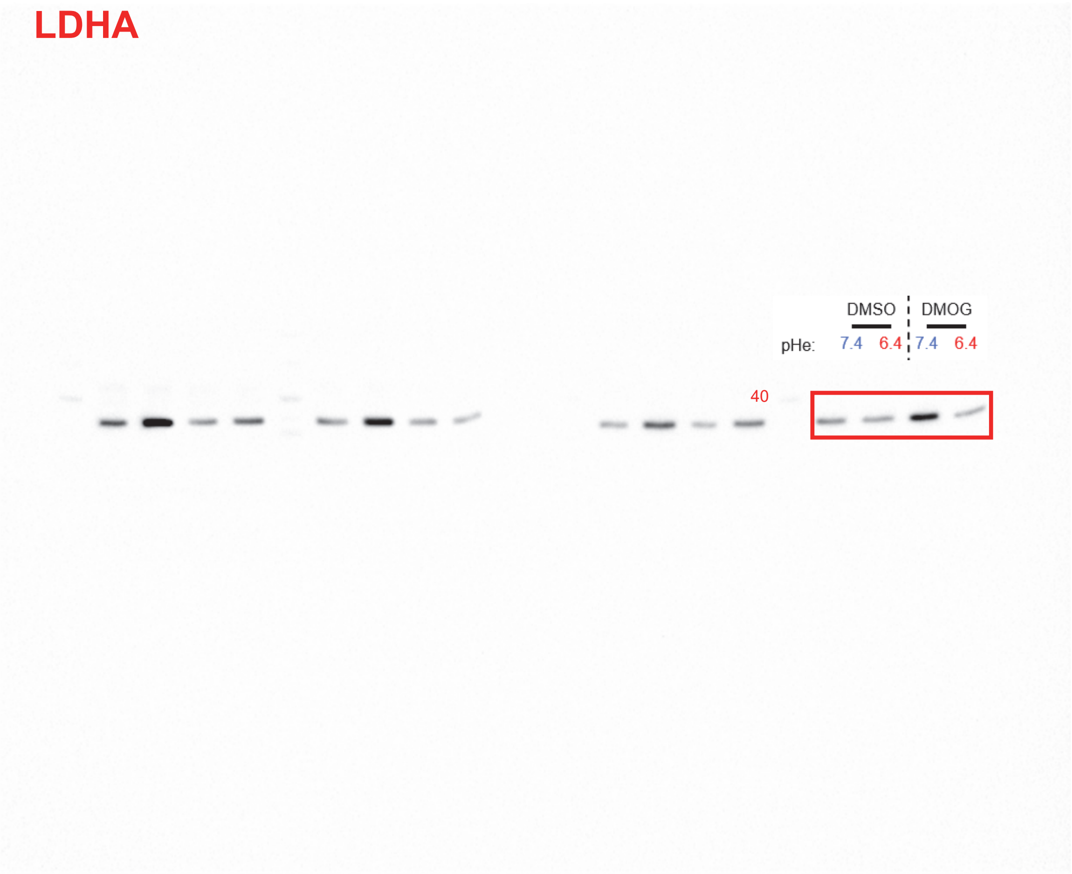

Figure 4

C

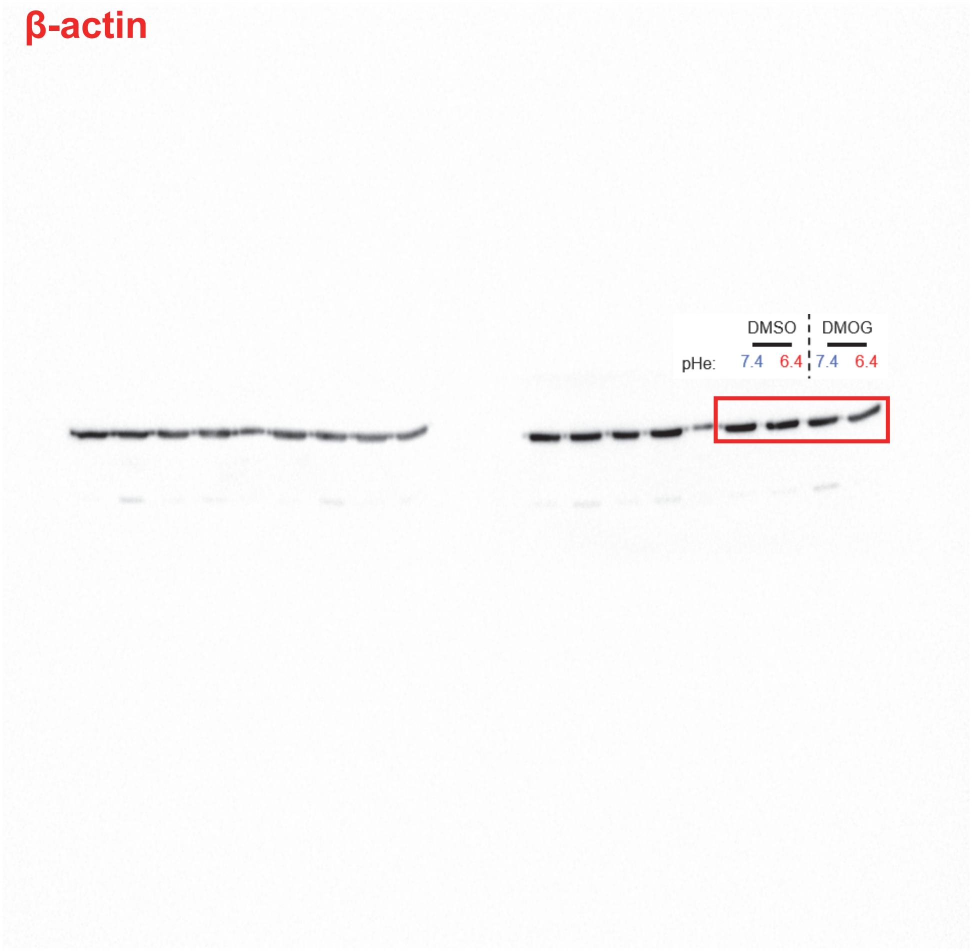

Figure 4

D

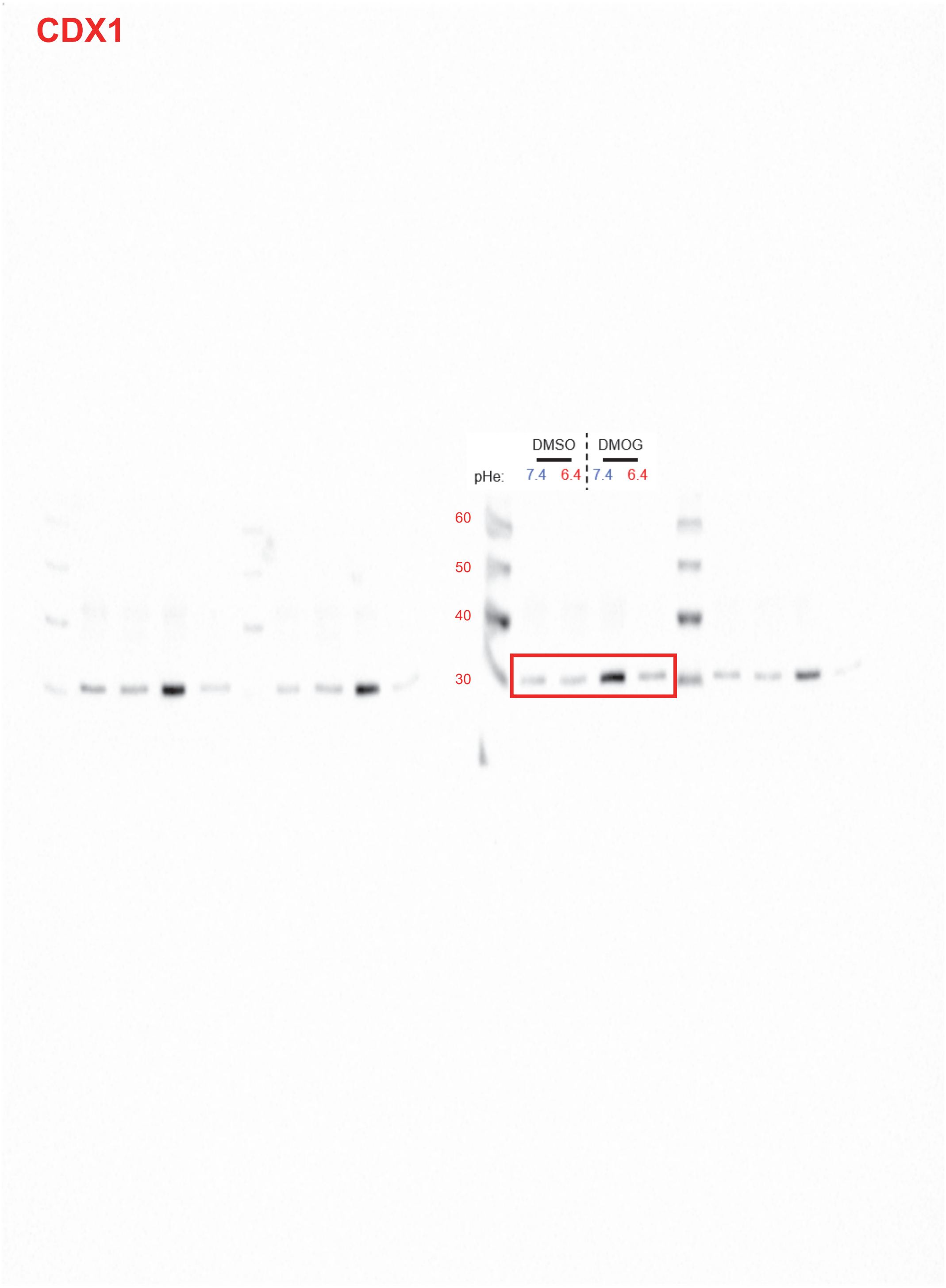

Figure 4

D

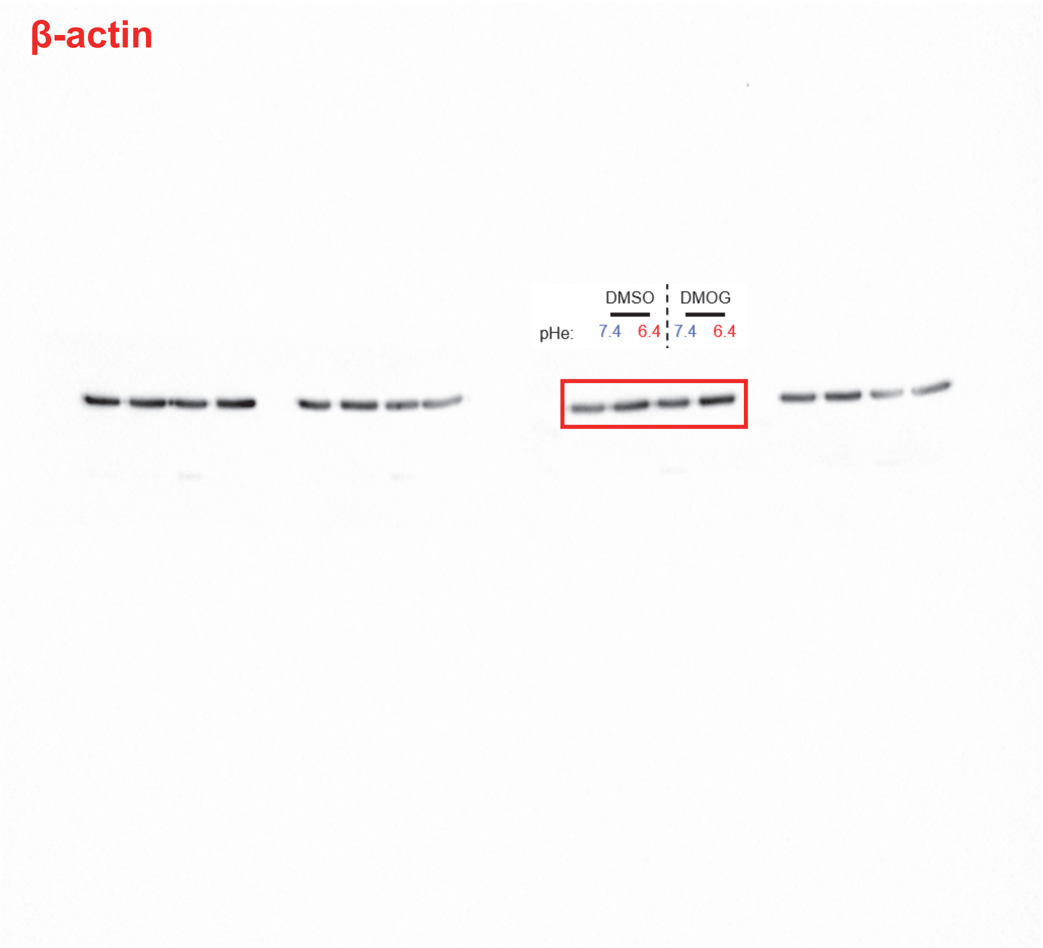

Figure 4

E

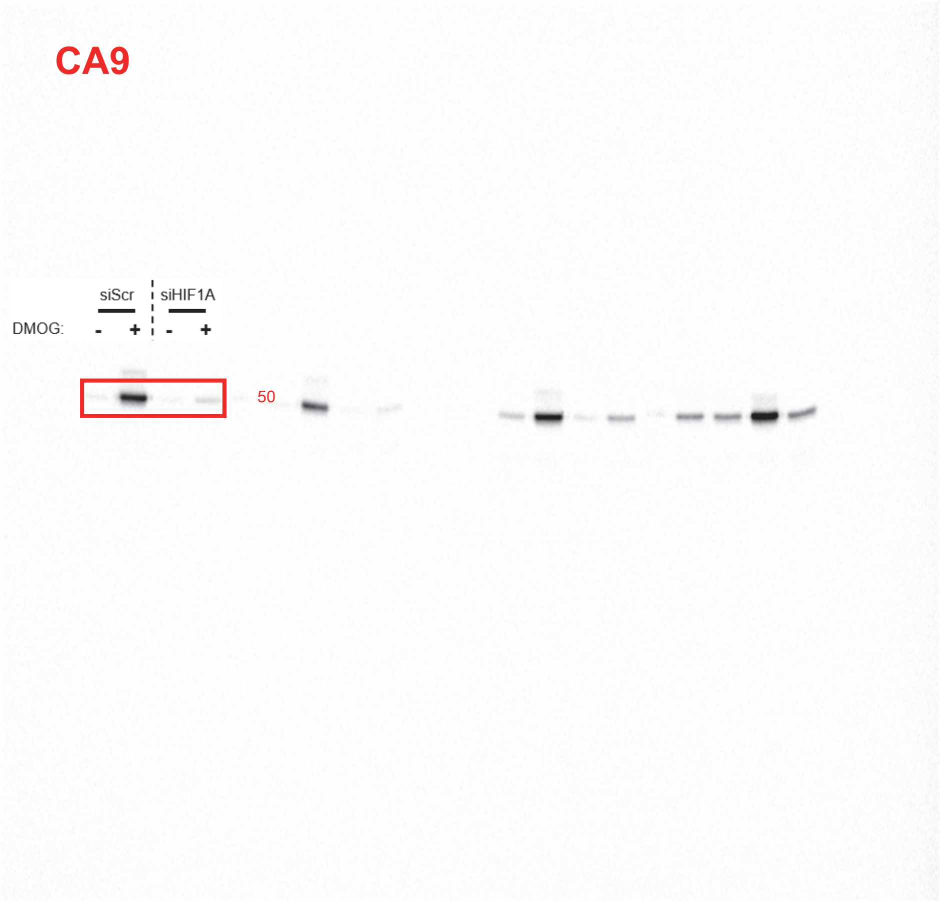

Figure 4

E

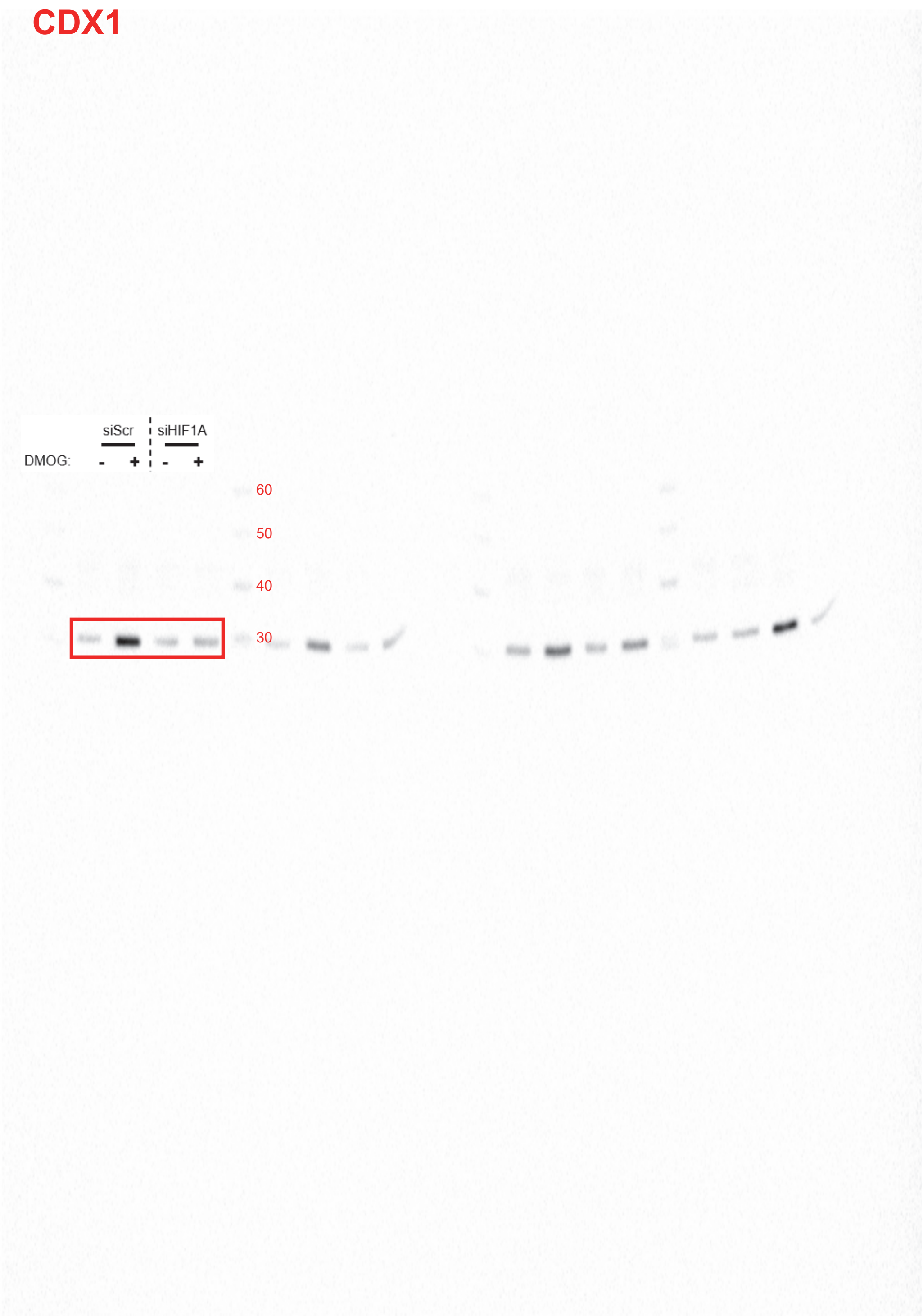

Figure 4

E

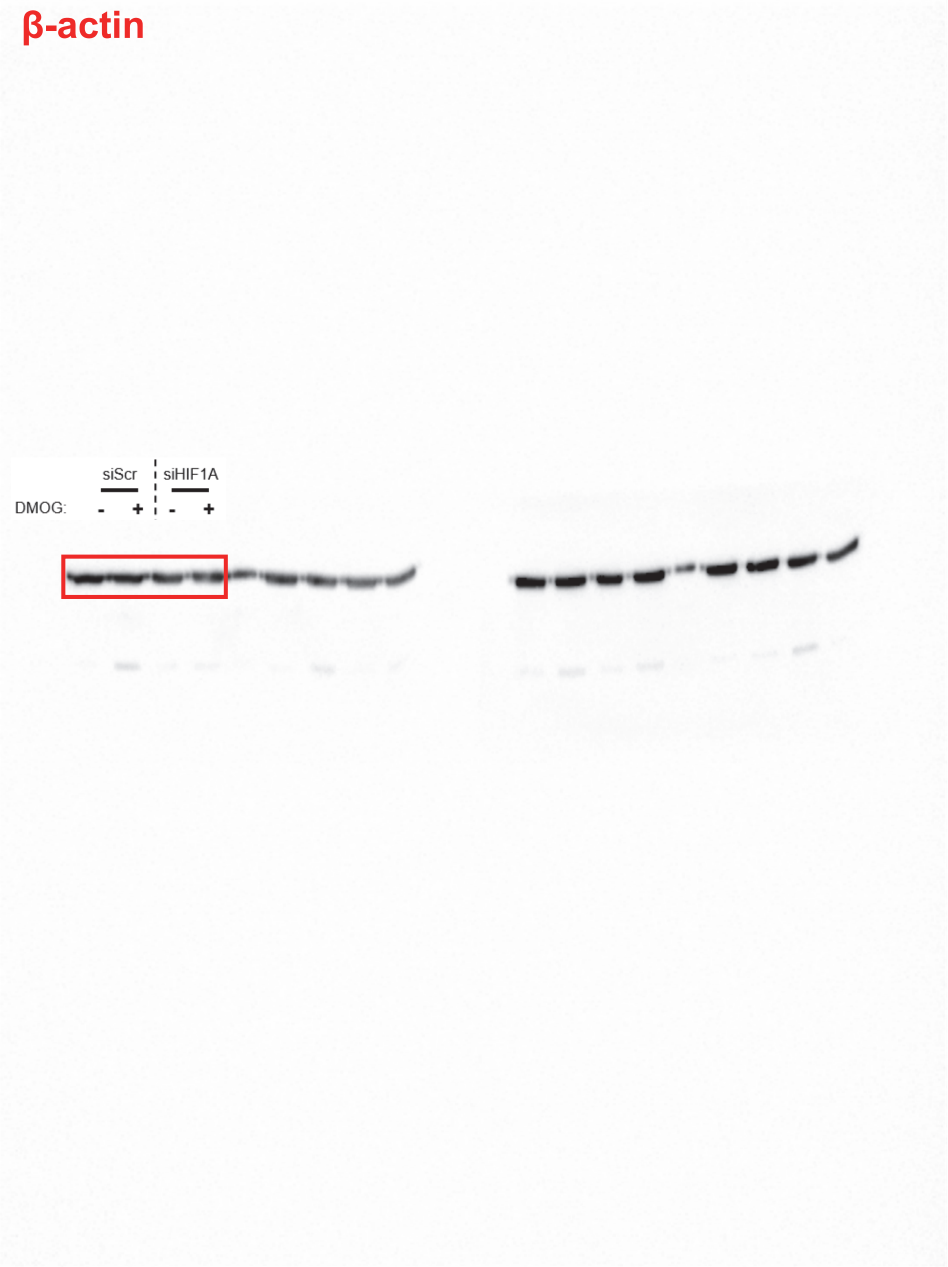

Figure 4

F

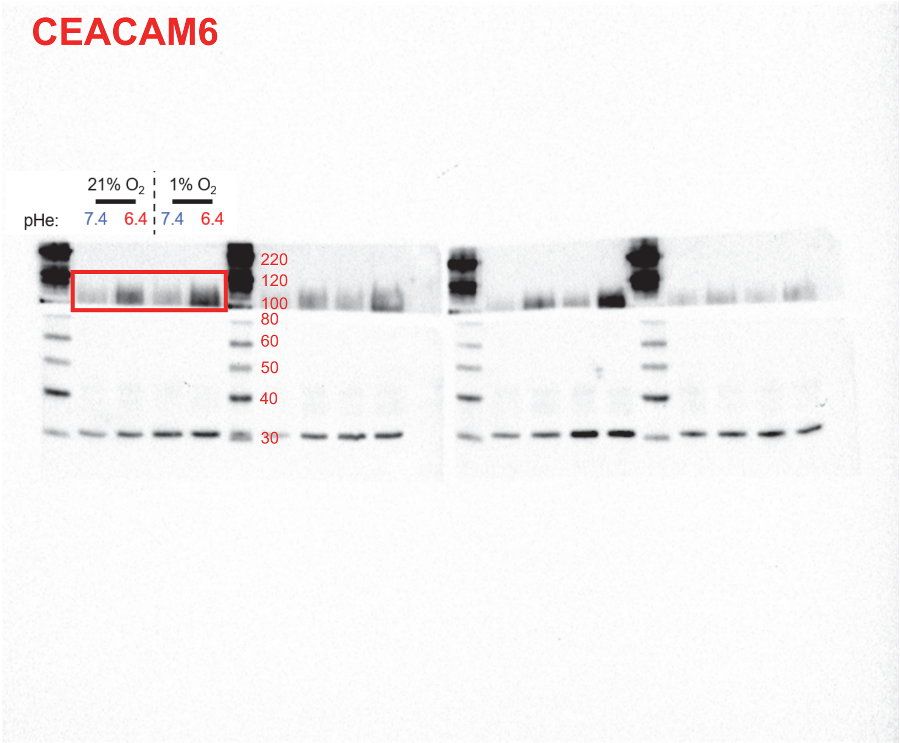
CEACAM6
21% O_2
1% O_2
pHe:
7.4
6.4
7.4
6.4
220
120
100
80
60
50
40
30

F

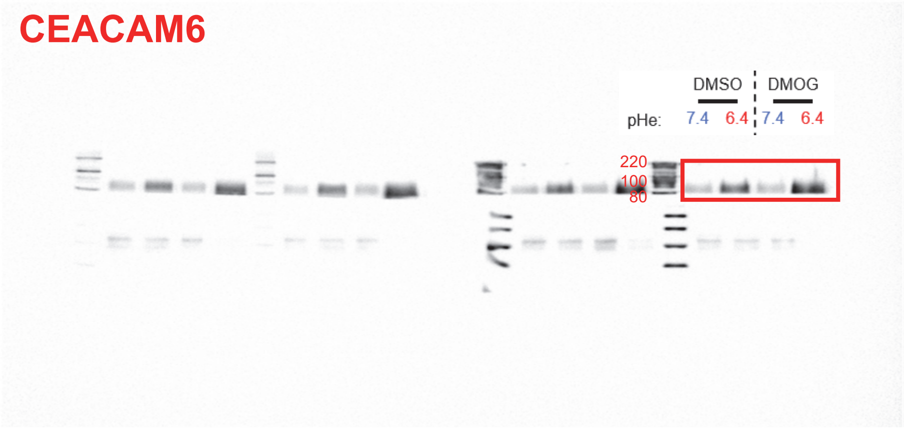
CEACAM6
DMSO
DMOG
pHe:
7.4
6.4
7.4
6.4
220
100
80

Figure 4

F

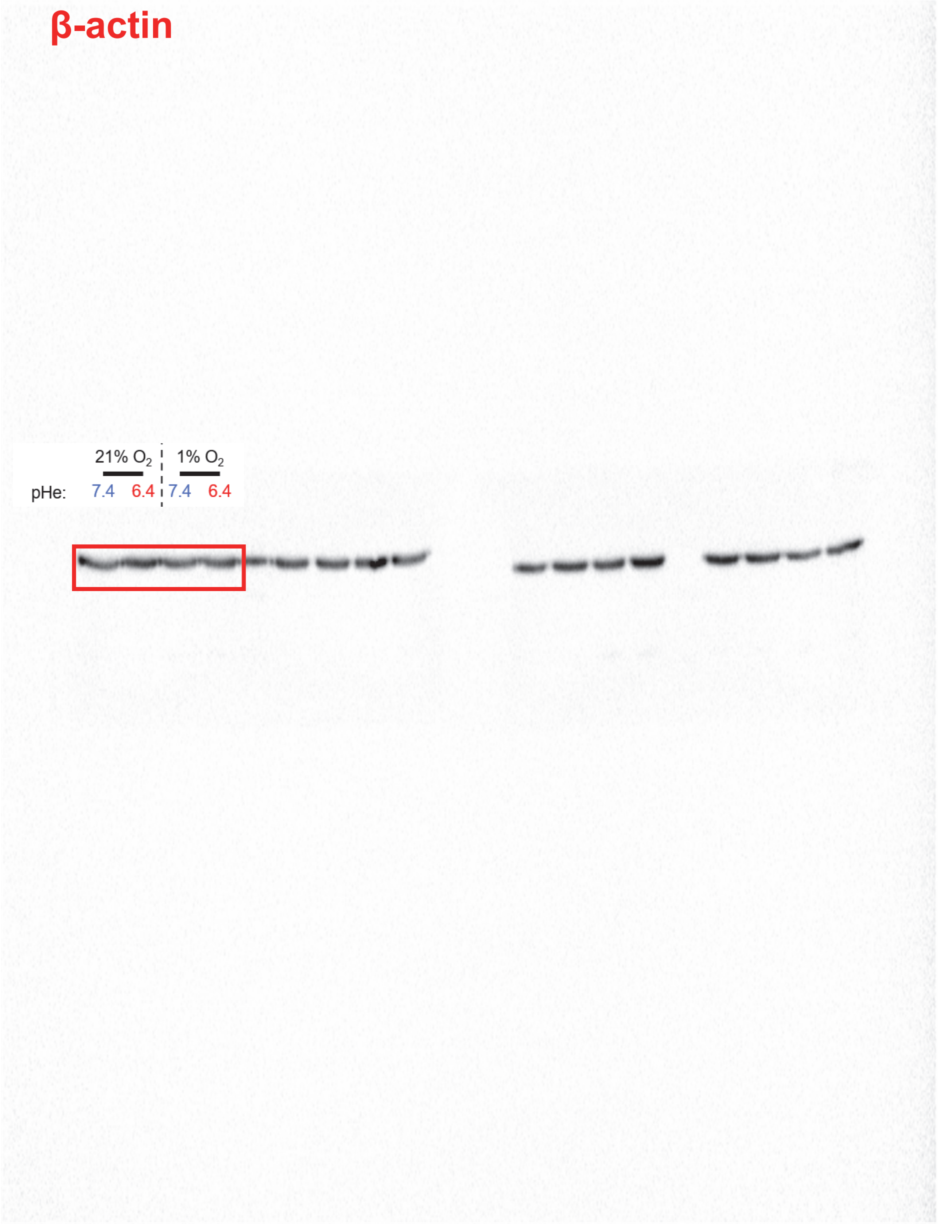

Figure 4

F

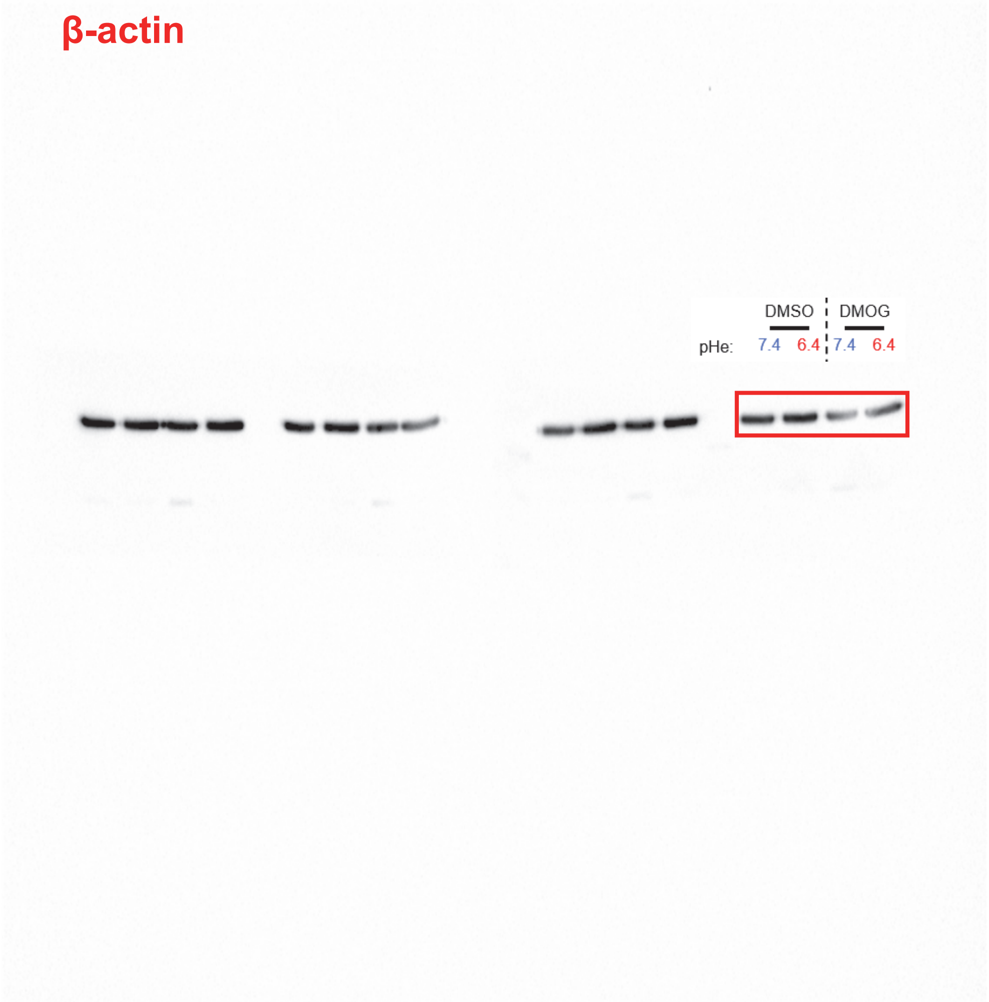

Supplement: SourceData F4 — is the source file for Fig. 4. [file jcb_202409103_sourcedataf4.pdf]
