# Supplementary material for: Acidosis attenuates the hypoxic stabilization of HIF-1α by activating lysosomal degradation
Source: J Cell Biol. 2025 Jun 24;224(8):e202409103. doi: 10.1083/jcb.202409103 (PMC12187095; doi:10.1083/jcb.202409103)
Supplement: SourceData F8 — is the source file for Fig. 8. [file jcb_202409103_sourcedataf8.pdf]

Figure 8

A

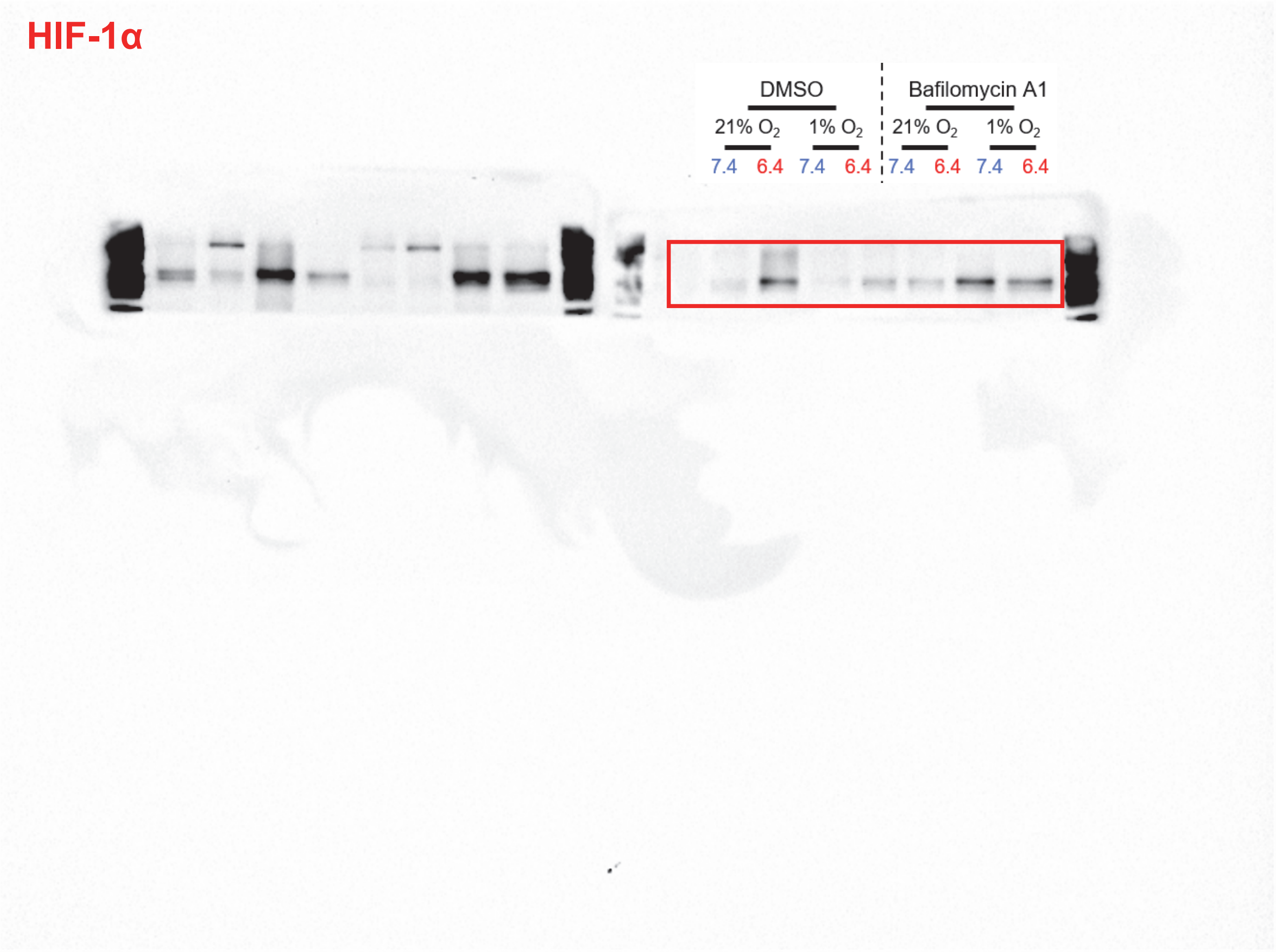
HIF-1α
DMSO
Bafilomycin A1
21% O2
1% O2
21% O2
1% O2
7.4 6.4 7.4 6.4 7.4 6.4 7.4 6.4

Figure 8

A

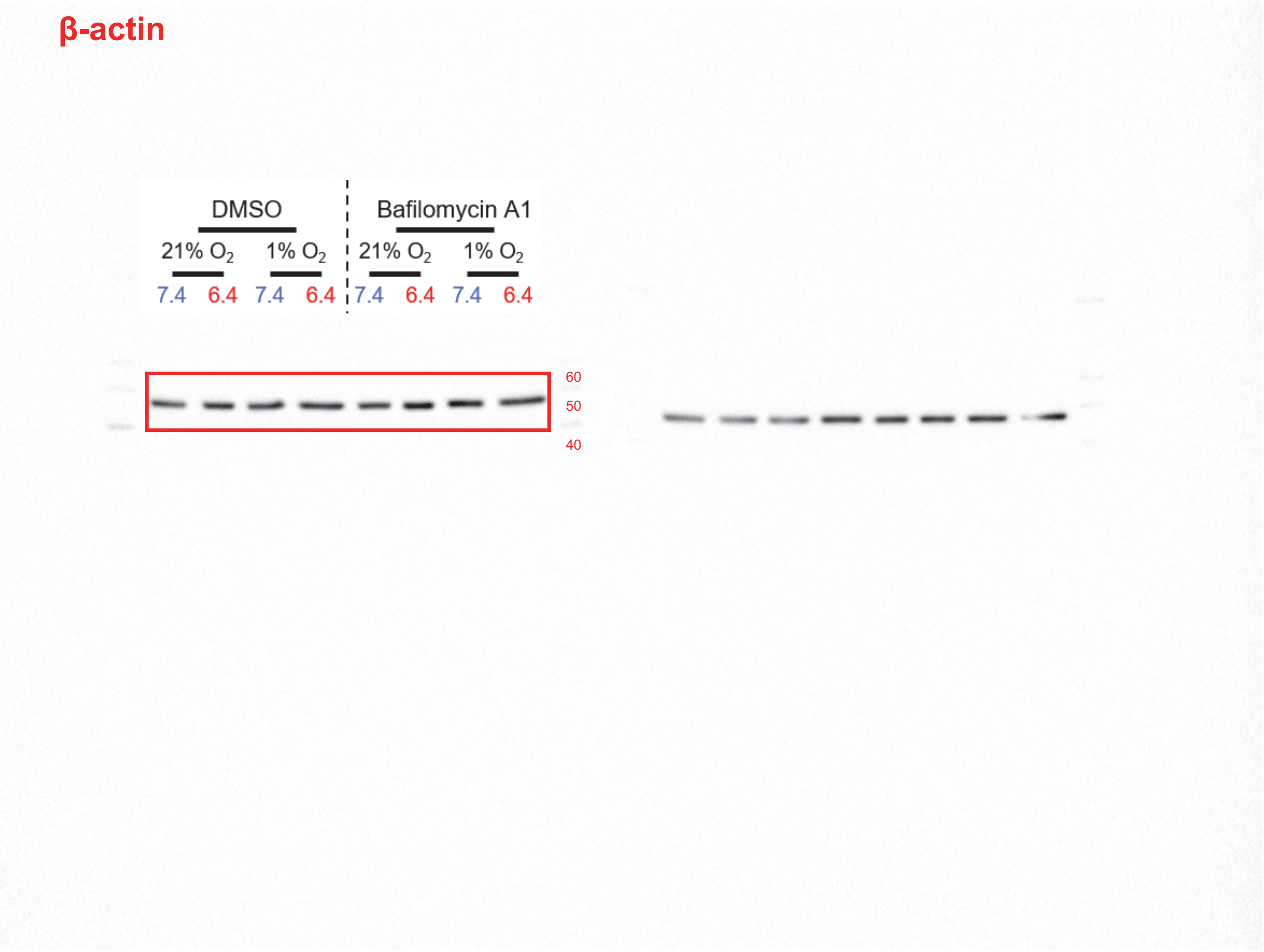
β-actin
DMSO
Bafilomycin A1
21% O2
1% O2
21% O2
1% O2
7.4
6.4
7.4
6.4
7.4
6.4
7.4
6.4
60
50
40

Figure 8

B

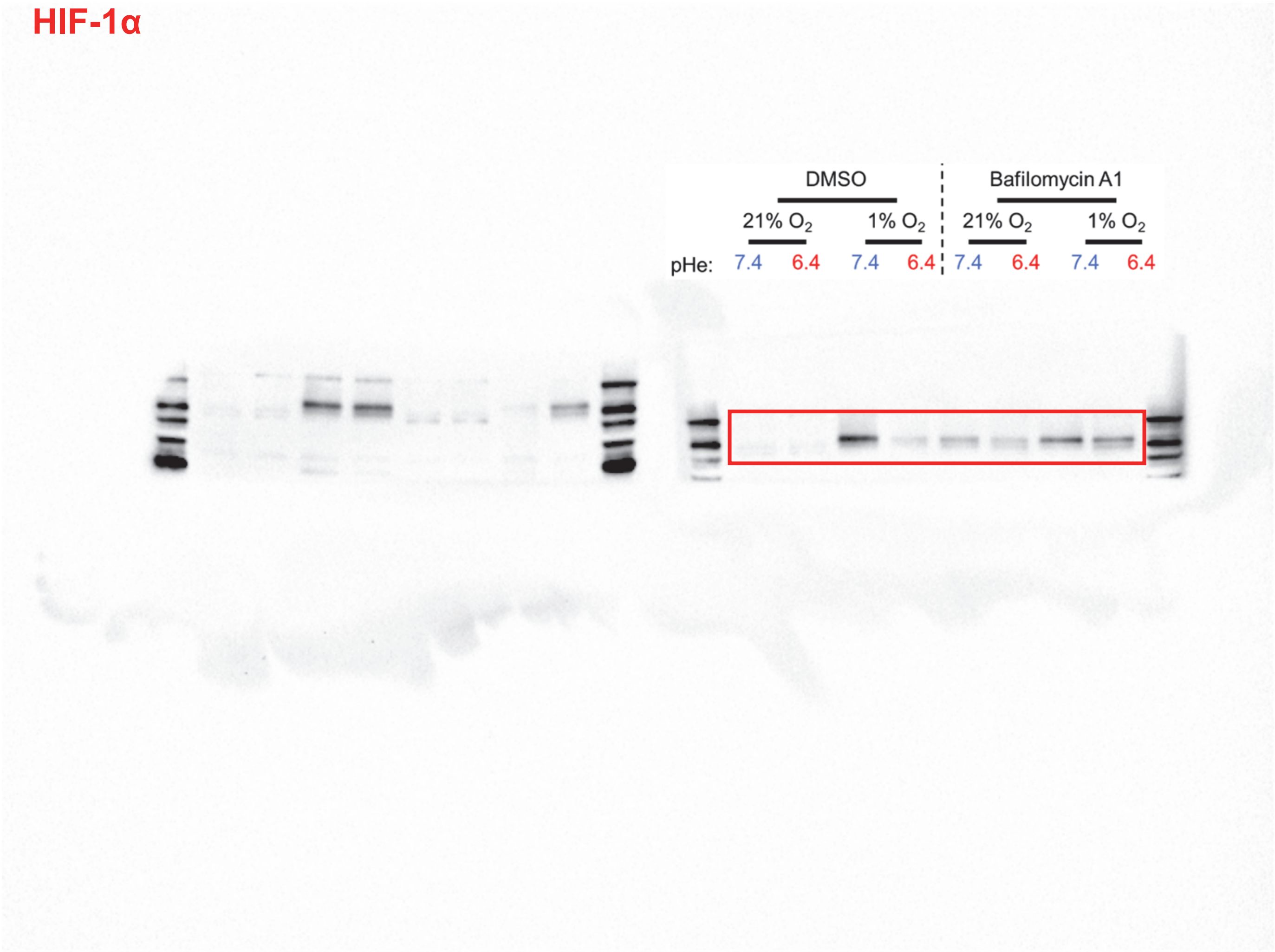

HIF-1α
DMSO
Bafilomycin A1
21% O2
1% O2
21% O2
1% O2
pHe:
7.4
6.4
7.4
6.4
7.4
6.4
7.4
6.4

Figure 8

B

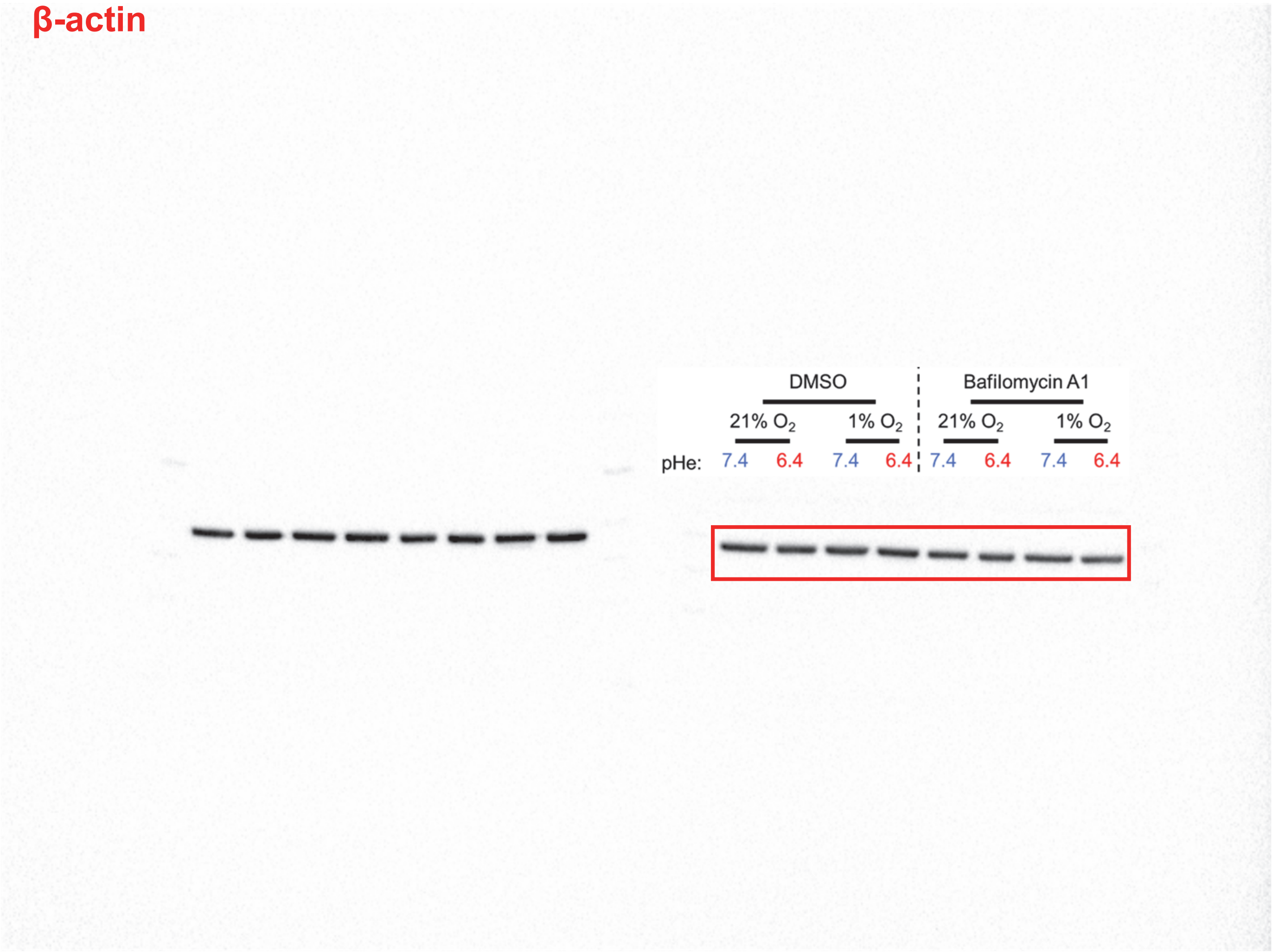
β-actin
DMSO
Bafilomycin A1
21% O2
1% O2
21% O2
1% O2
pHe: 7.4 6.4 7.4 6.4 7.4 6.4 7.4 6.4

Figure 8

C

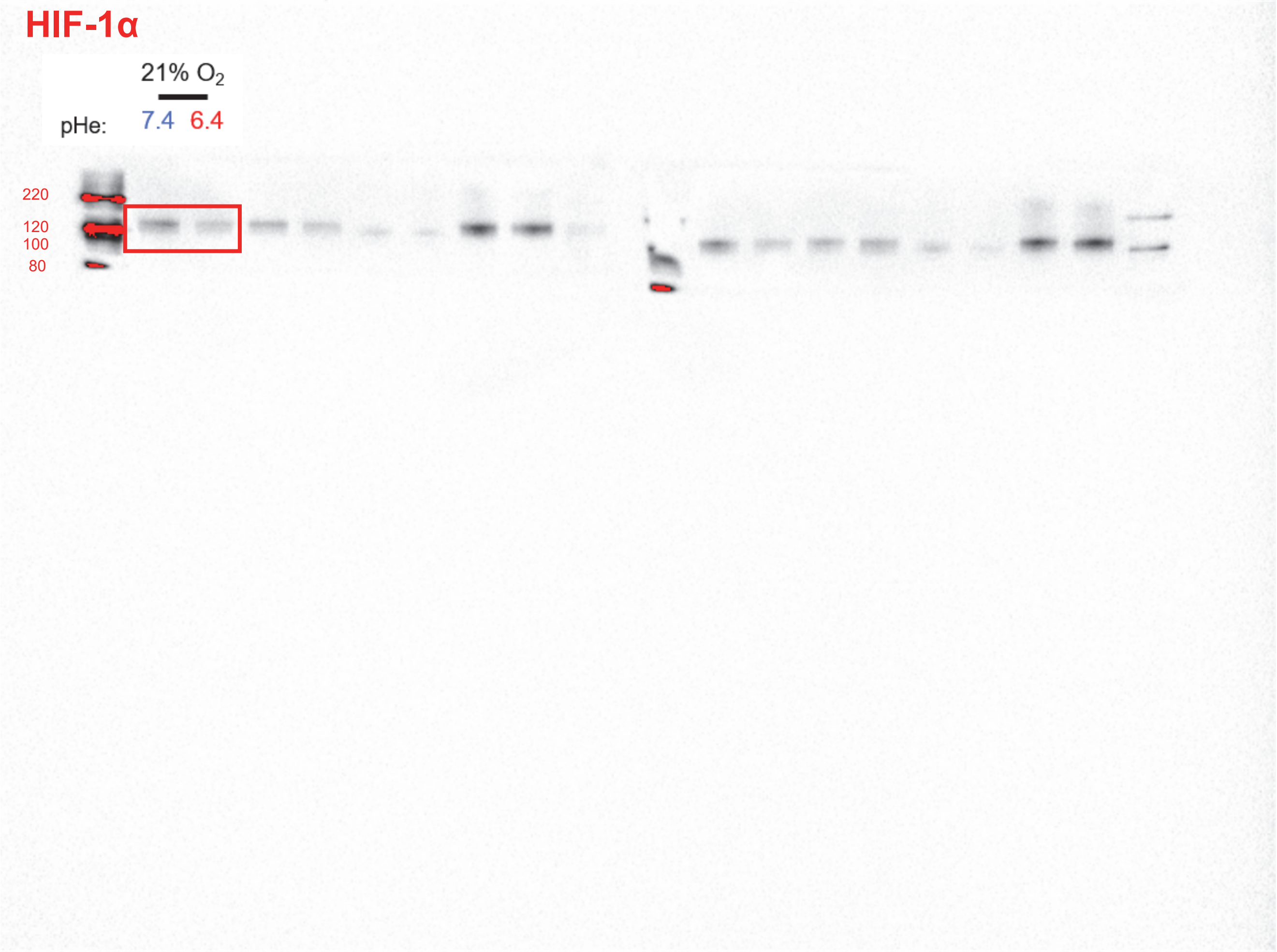

C

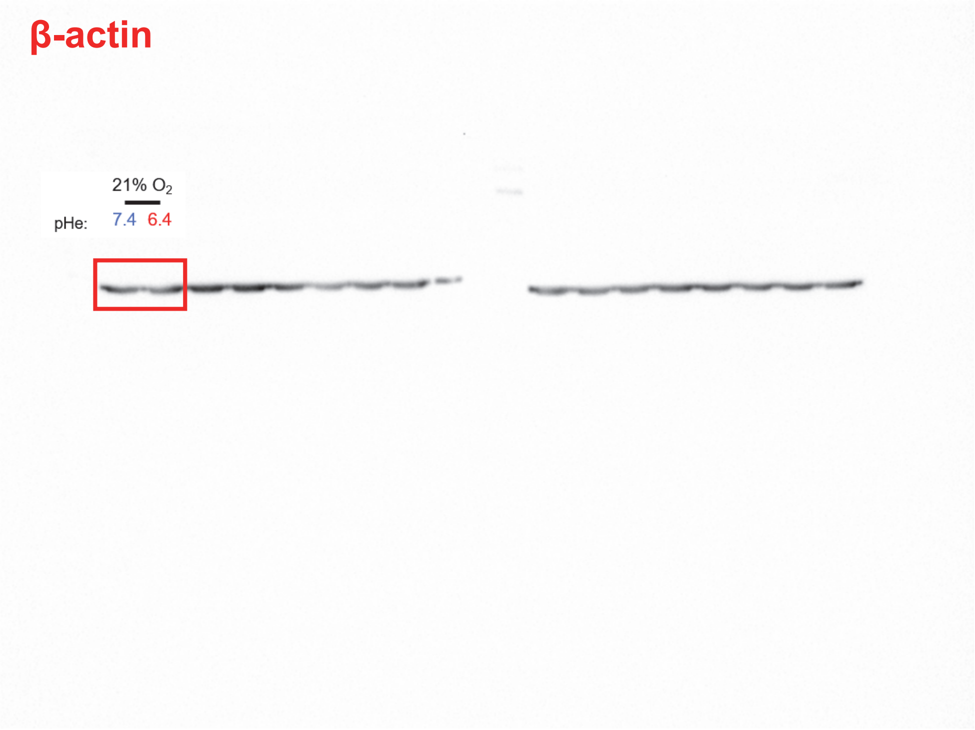

Figure 8

D

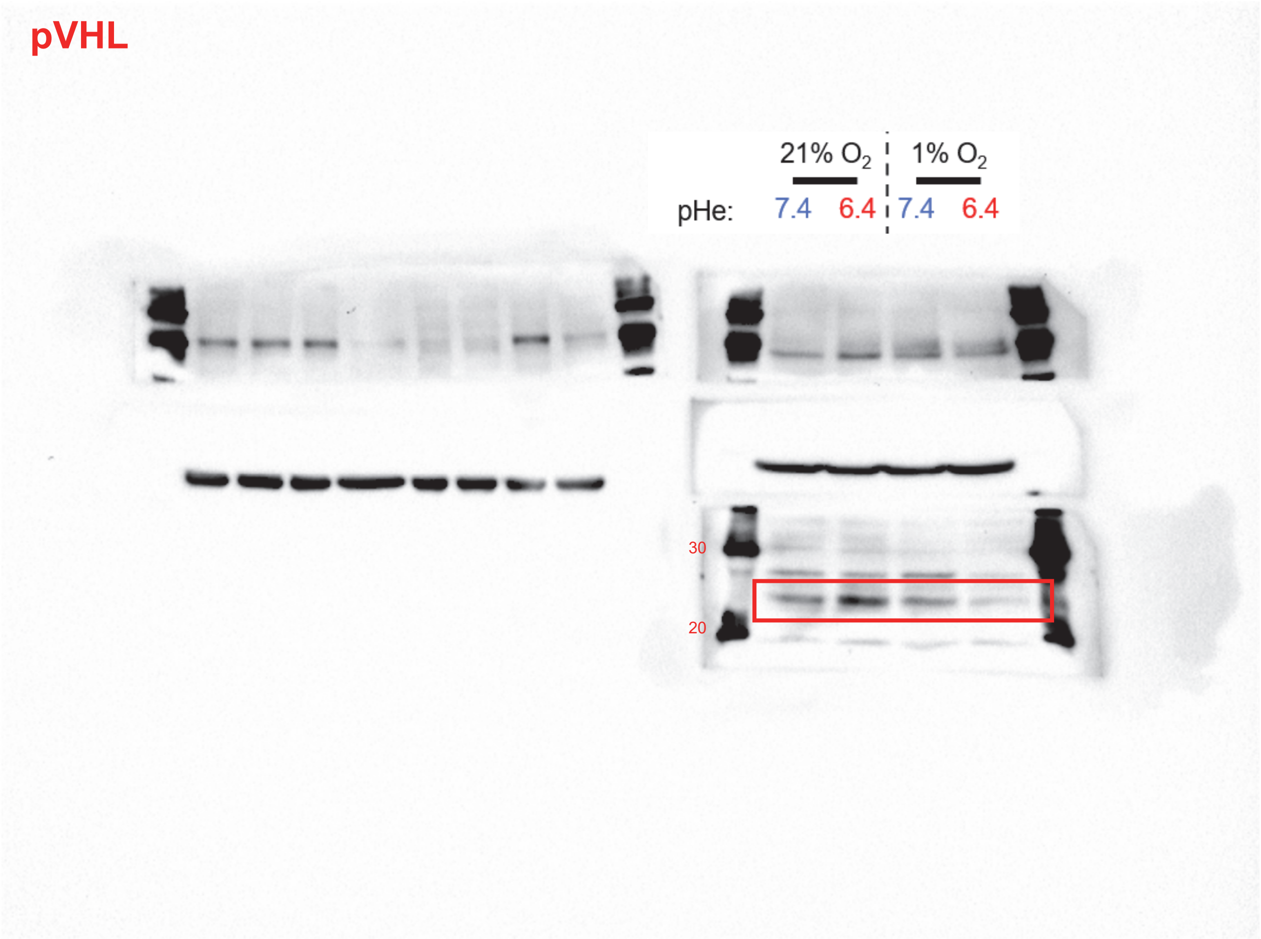

Figure 8

D

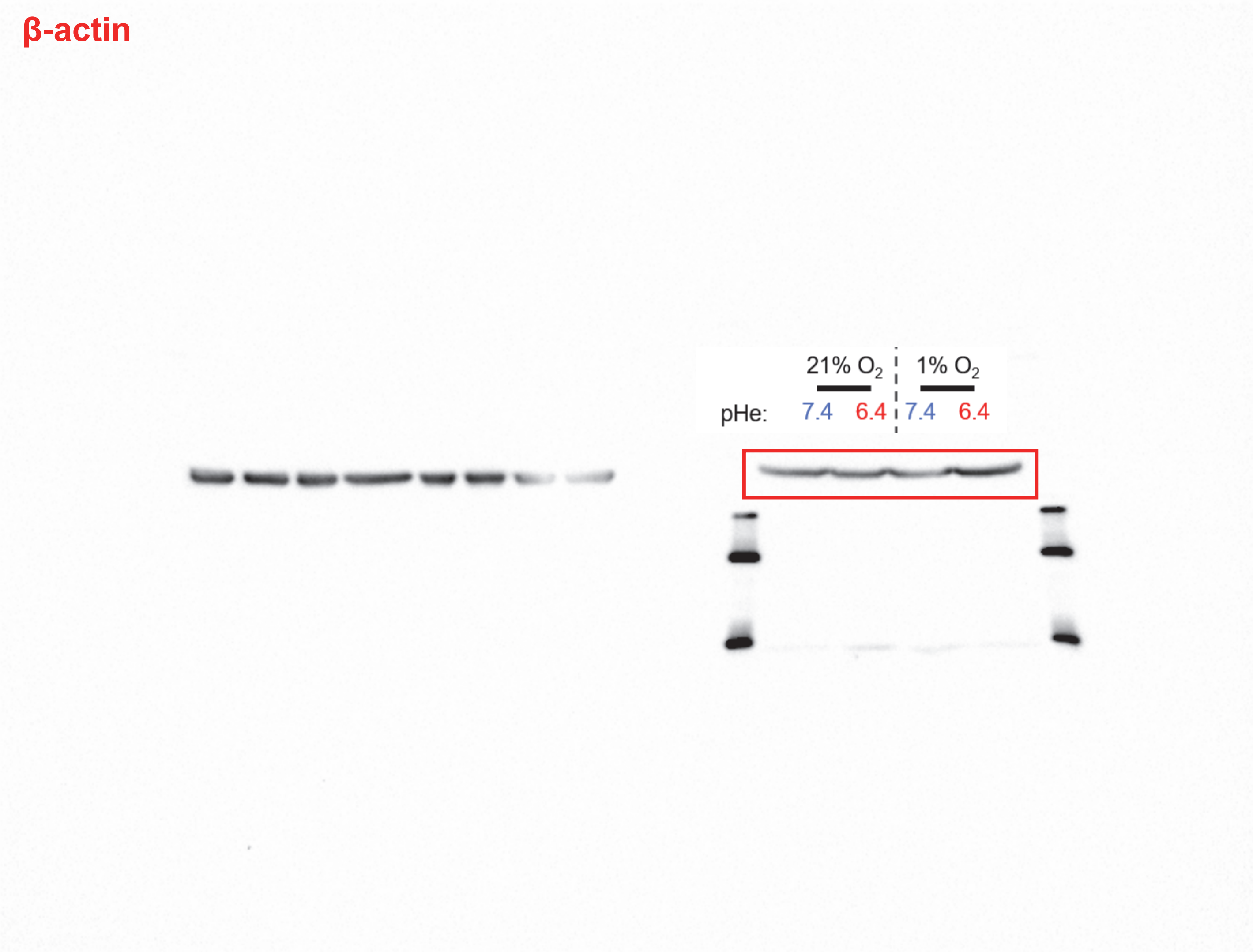

Figure 8

E

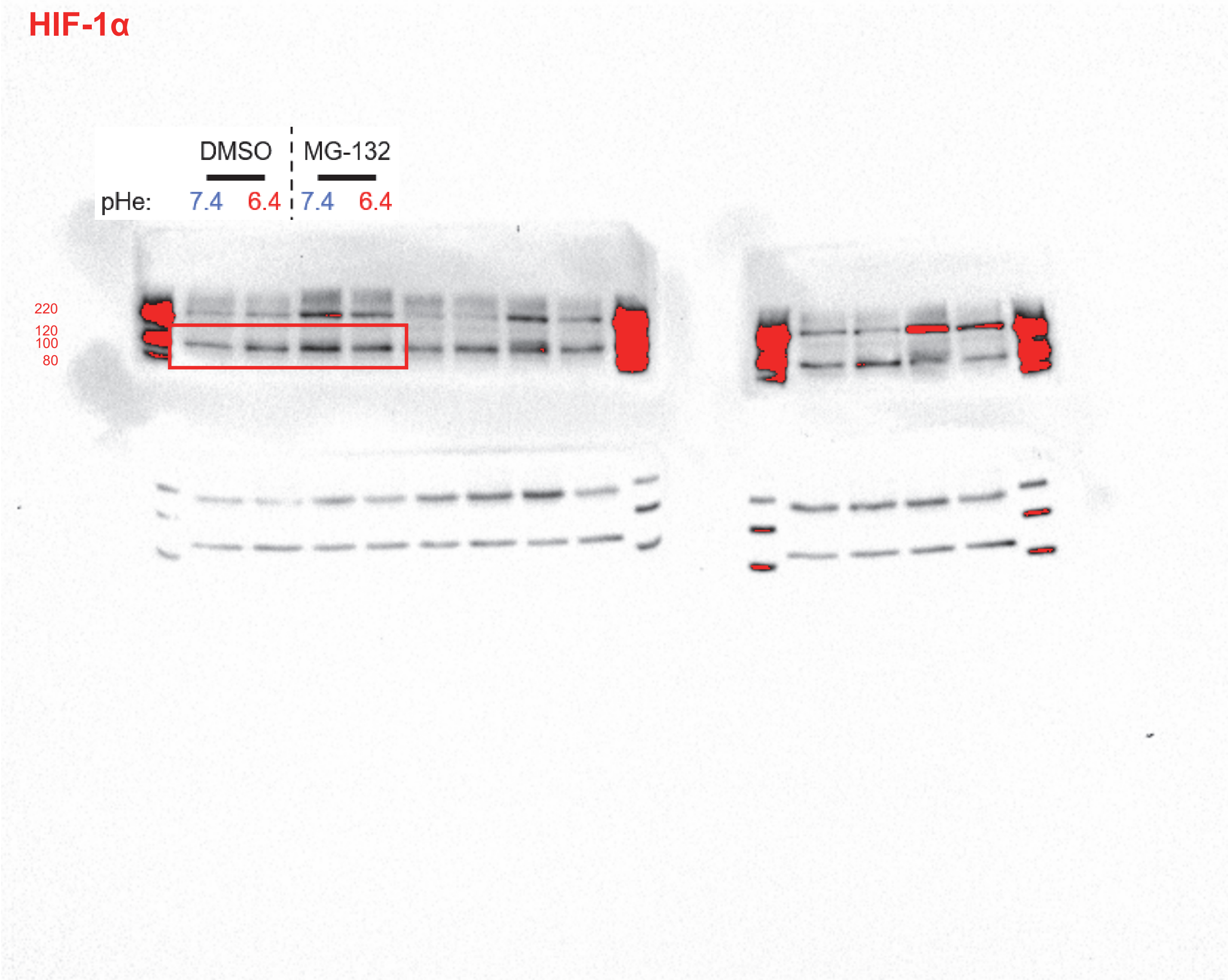

Figure 8

E

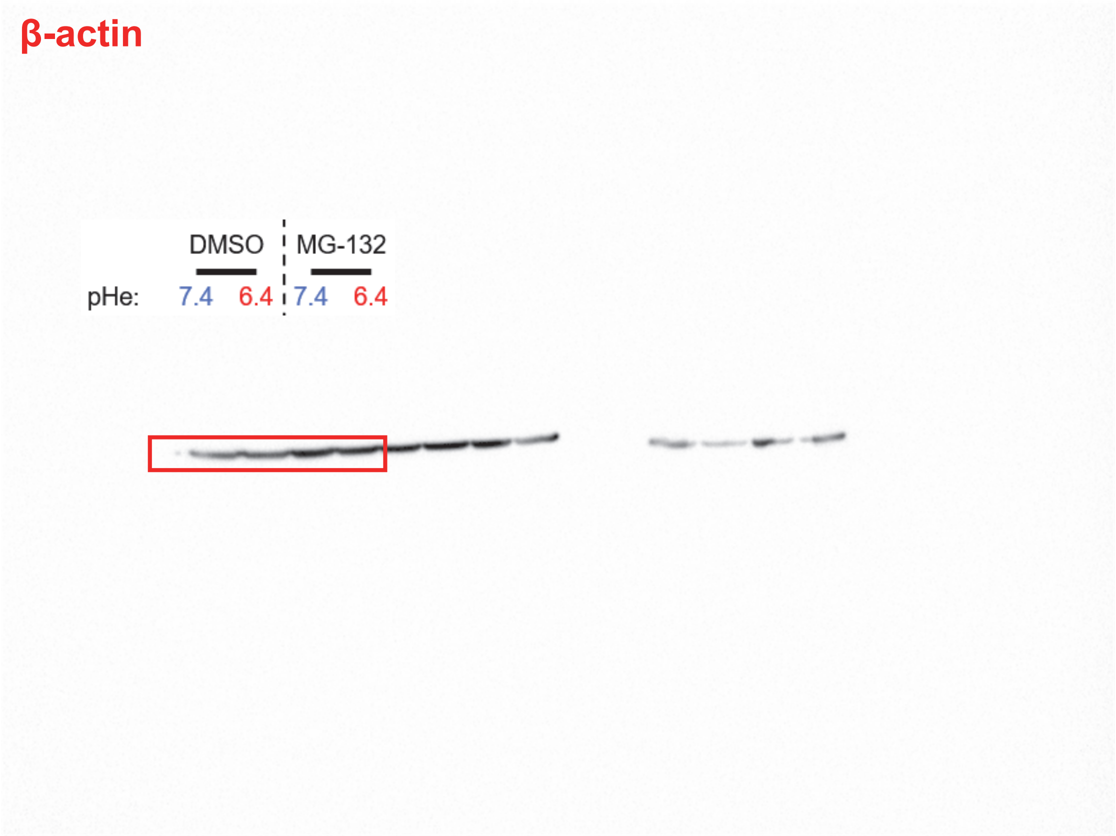

Figure 8

F

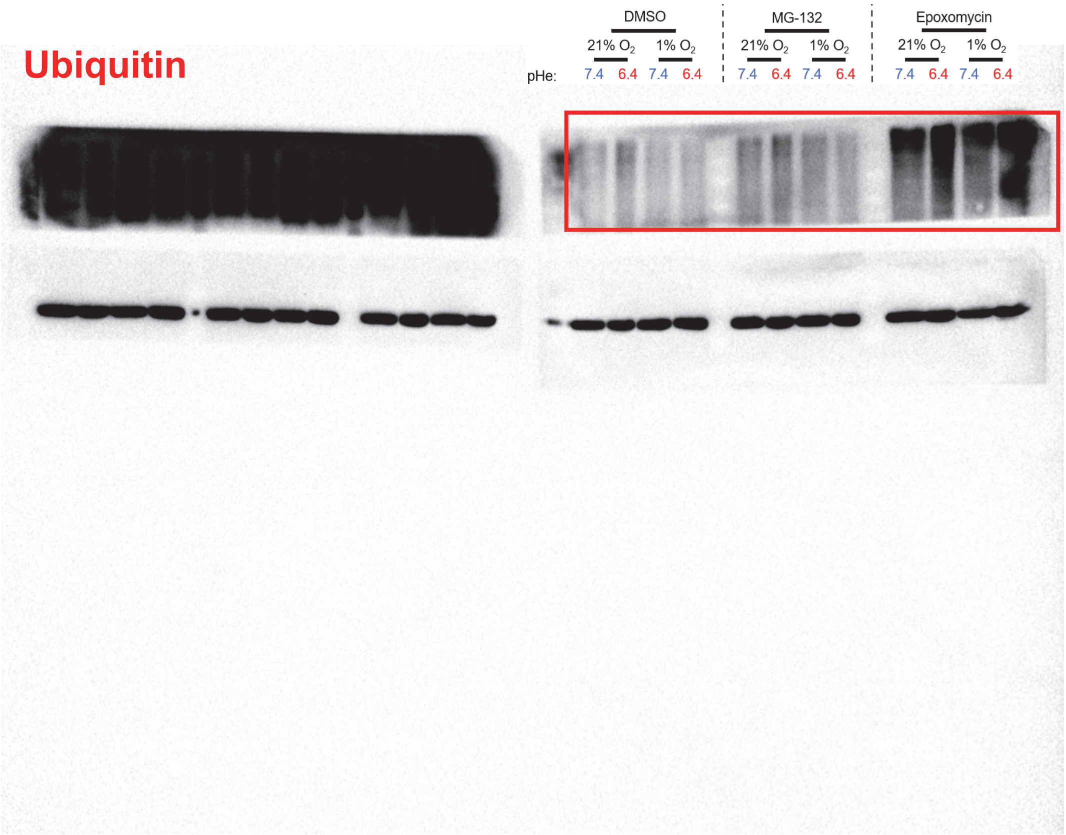
Ubiquitin
DMSO
MG-132
Epoxomycin
21% O_2
1% O_2
pHe:
7.4
6.4

F

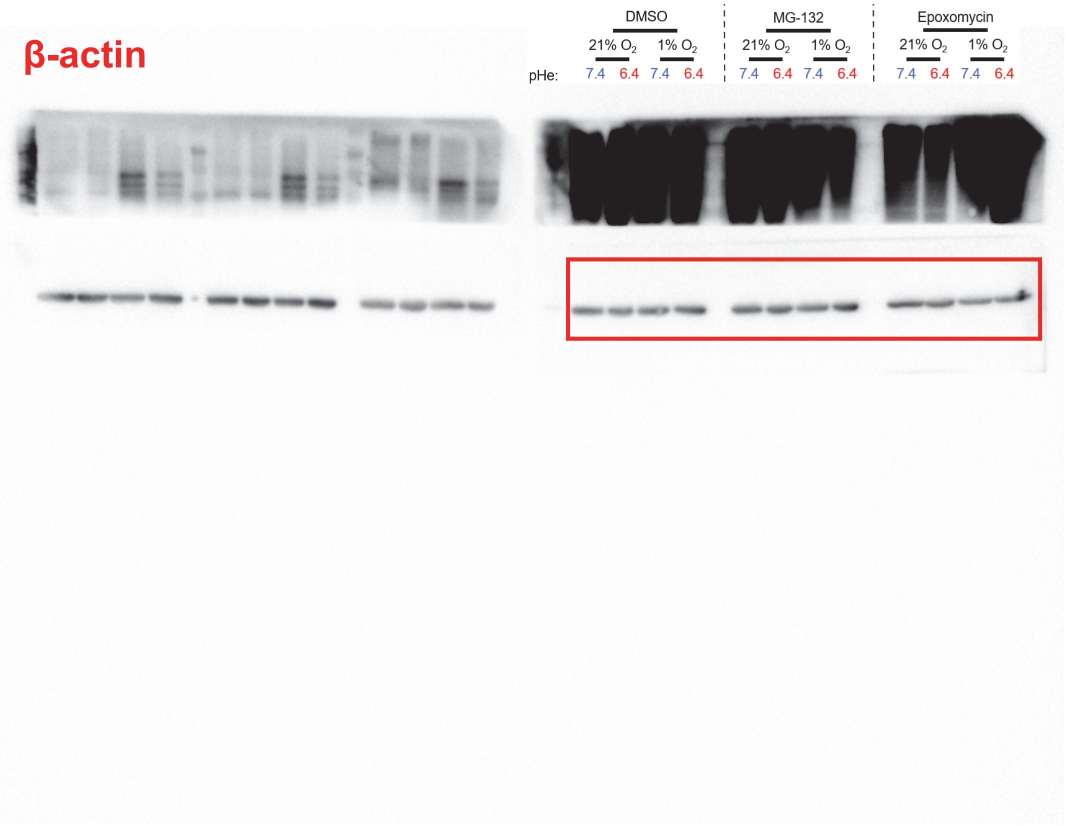
β-actin
DMSO
MG-132
Epoxomycin
21% O_2
1% O_2
pHe:
7.4
6.4

Figure 8

G

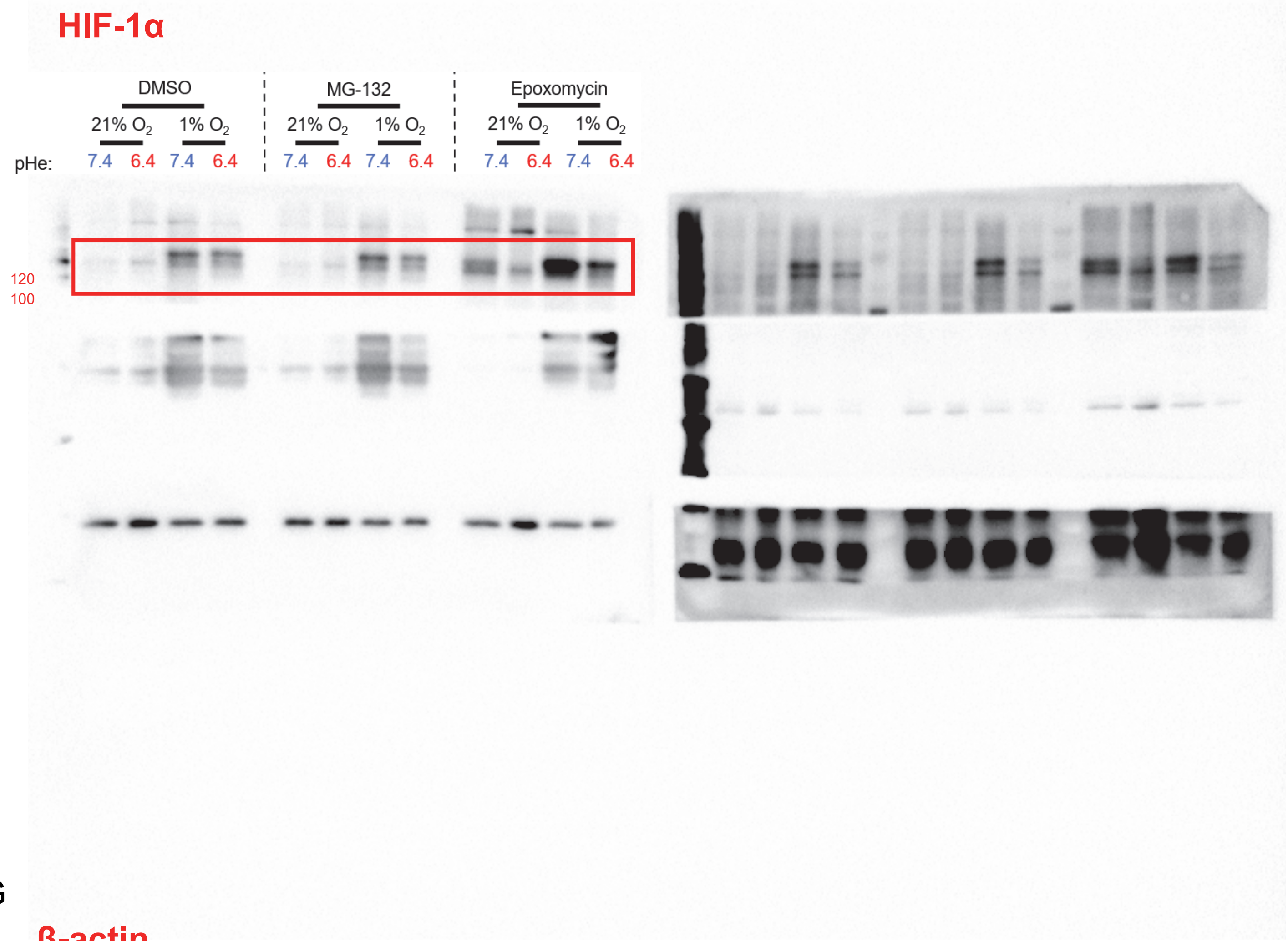
HIF-1α
DMSO
MG-132
Epoxomycin
21% O2
1% O2
21% O2
1% O2
21% O2
1% O2
pHe:
7.4 6.4 7.4 6.4 7.4 6.4 7.4 6.4 7.4 6.4 7.4 6.4
120
100

G

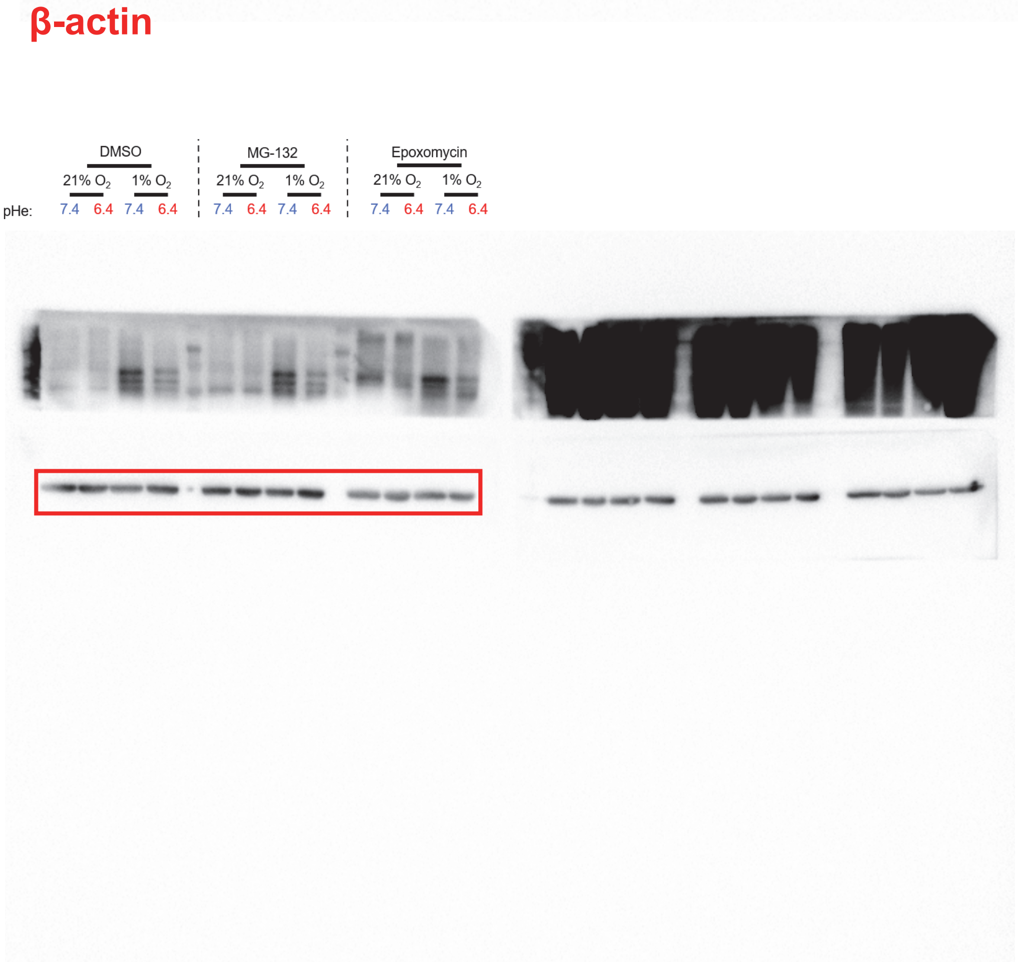
β-actin
DMSO
MG-132
Epoxomycin
21% O2
1% O2
21% O2
1% O2
21% O2
1% O2
pHe:
7.4 6.4 7.4 6.4 7.4 6.4 7.4 6.4 7.4 6.4 7.4 6.4
