# Supplementary material for: Acidosis attenuates the hypoxic stabilization of HIF-1α by activating lysosomal degradation
Source: J Cell Biol. 2025 Jun 24;224(8):e202409103. doi: 10.1083/jcb.202409103 (PMC12187095; doi:10.1083/jcb.202409103)

Figure10

A

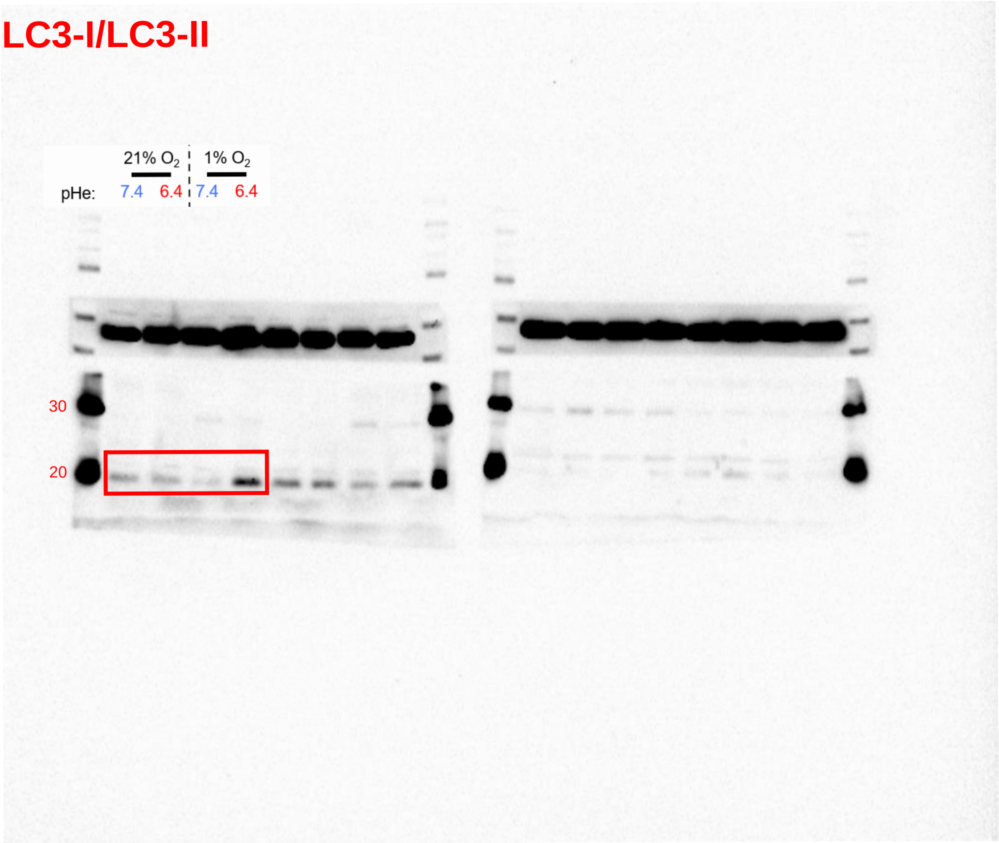

LC3-I/LC3-II
21% O_2
1% O_2
pHe: 7.4 6.4 7.4 6.4
30
20

A

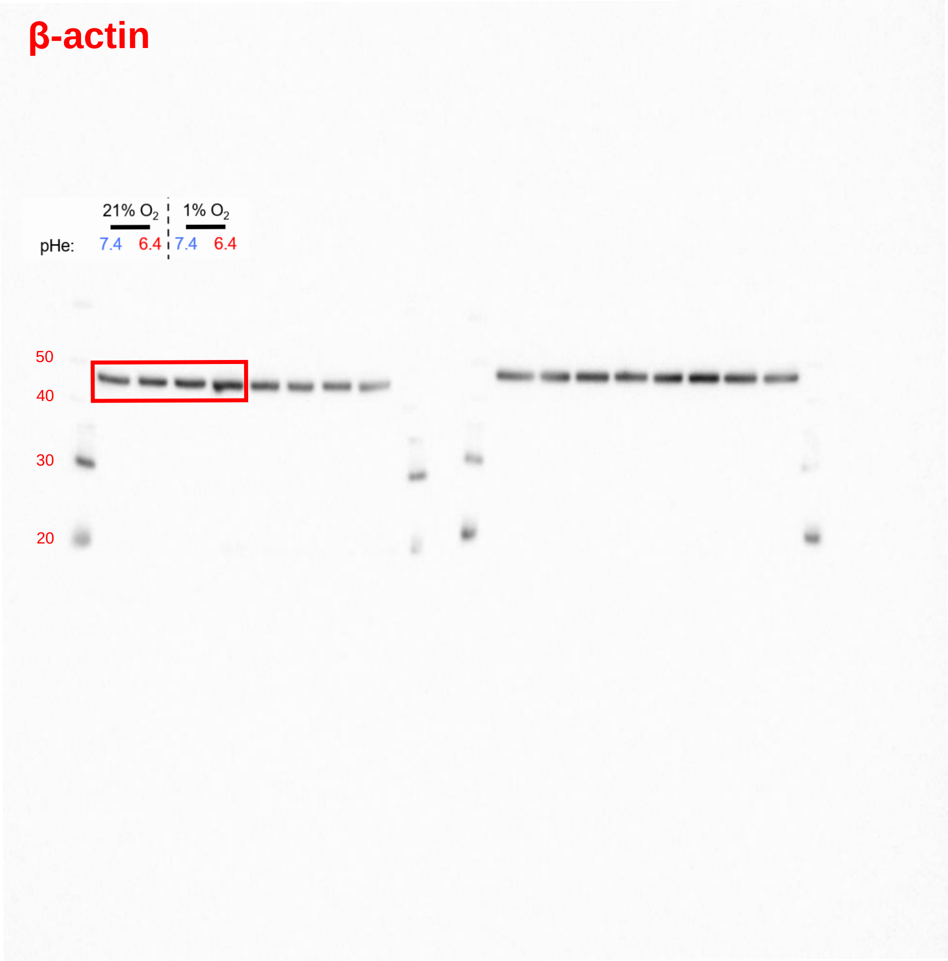

β-actin
21% O_2
1% O_2
pHe: 7.4 6.4 7.4 6.4
50
40
30
20

Figure 10

B

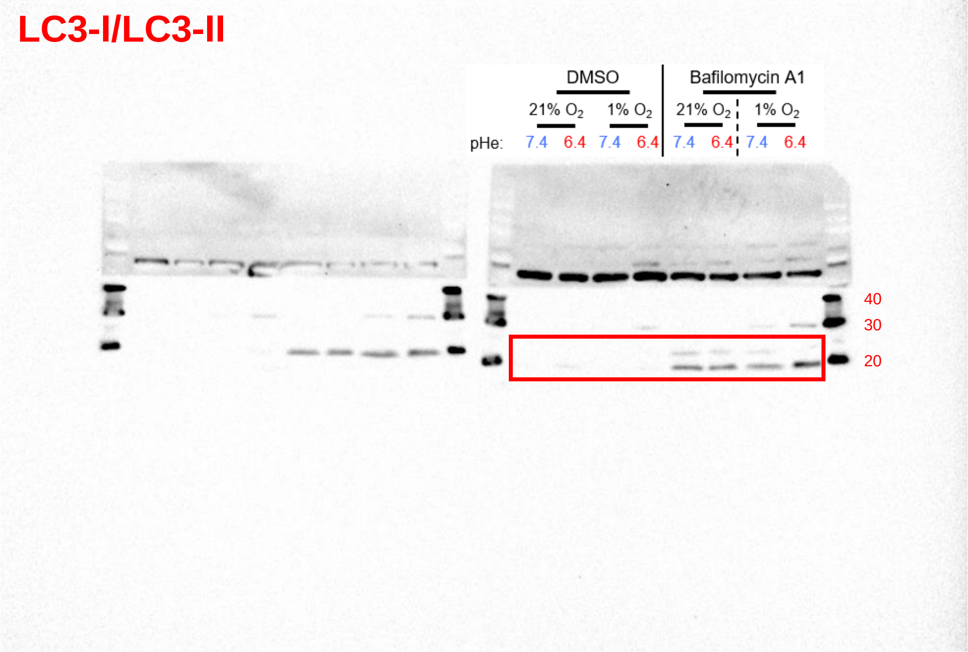
LC3-I/LC3-II
DMSO
Bafilomycin A1
21% O2
1% O2
21% O2
1% O2
pHe:
7.4
6.4
7.4
6.4
7.4
6.4
7.4
6.4
40
30
20

B

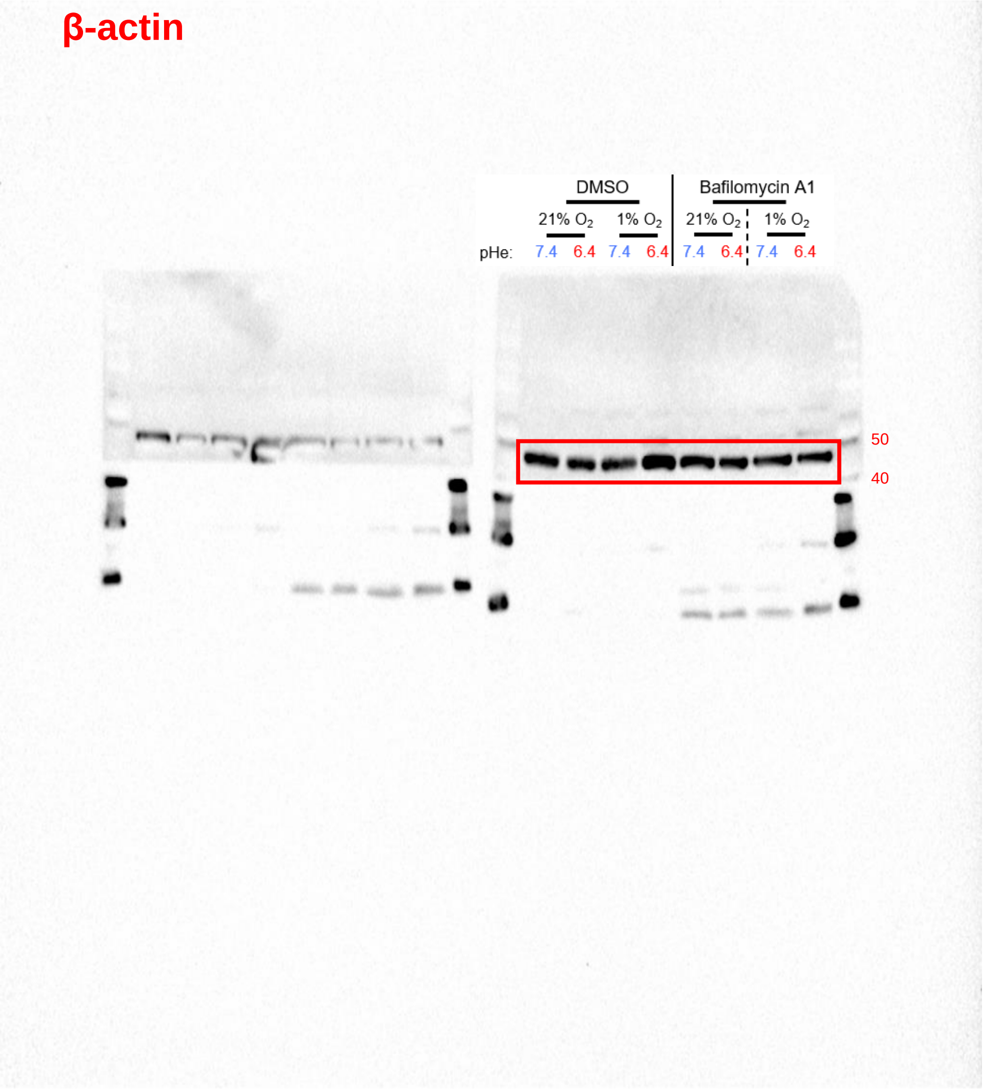
β-actin
DMSO
Bafilomycin A1
21% O2
1% O2
21% O2
1% O2
pHe:
7.4
6.4
7.4
6.4
7.4
6.4
7.4
6.4
50
40

Figure 10

C

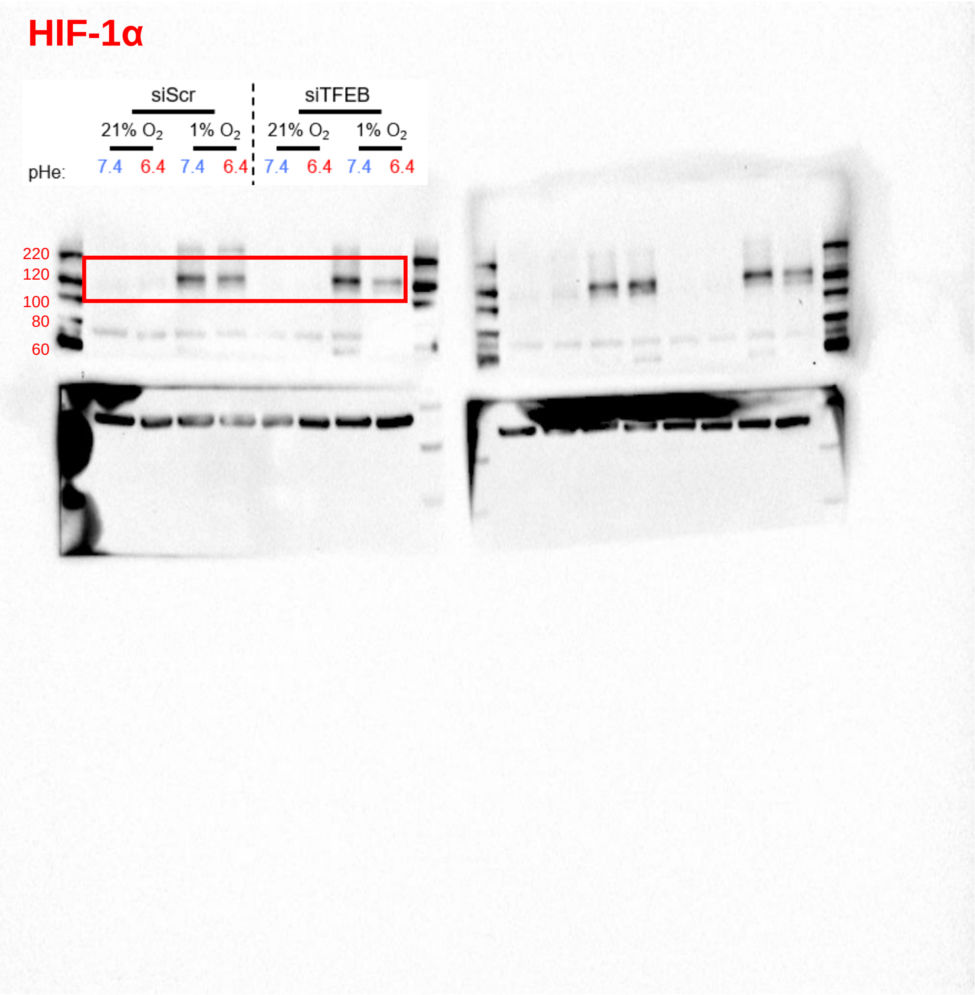
HIF-1α
siScr
siTFEB
21% O_2
1% O_2
21% O_2
1% O_2
pHe:
7.4
6.4
7.4
6.4
7.4
6.4
7.4
6.4
220
120
100
80
60

Figure 10

C

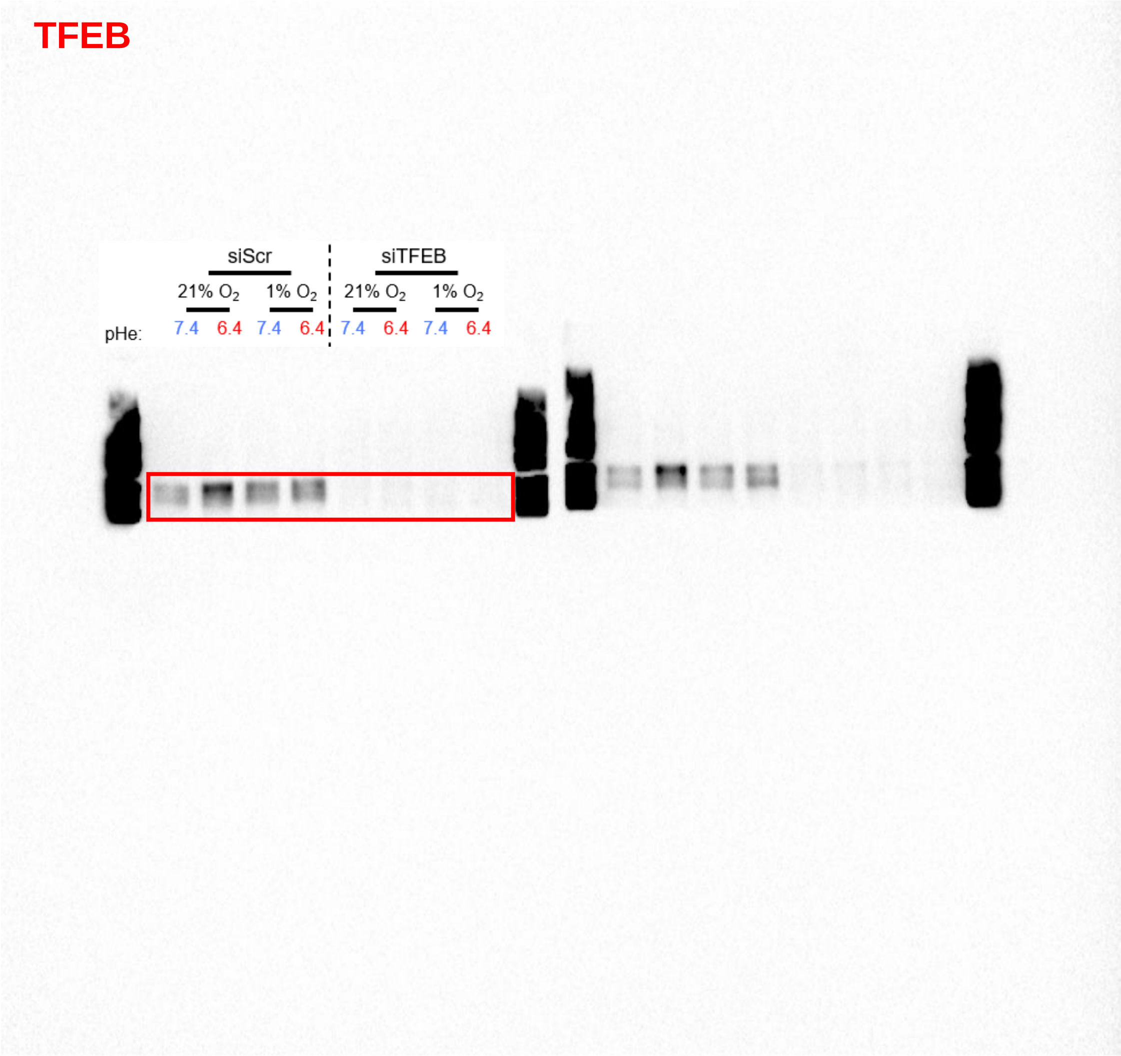

Figure 10

C

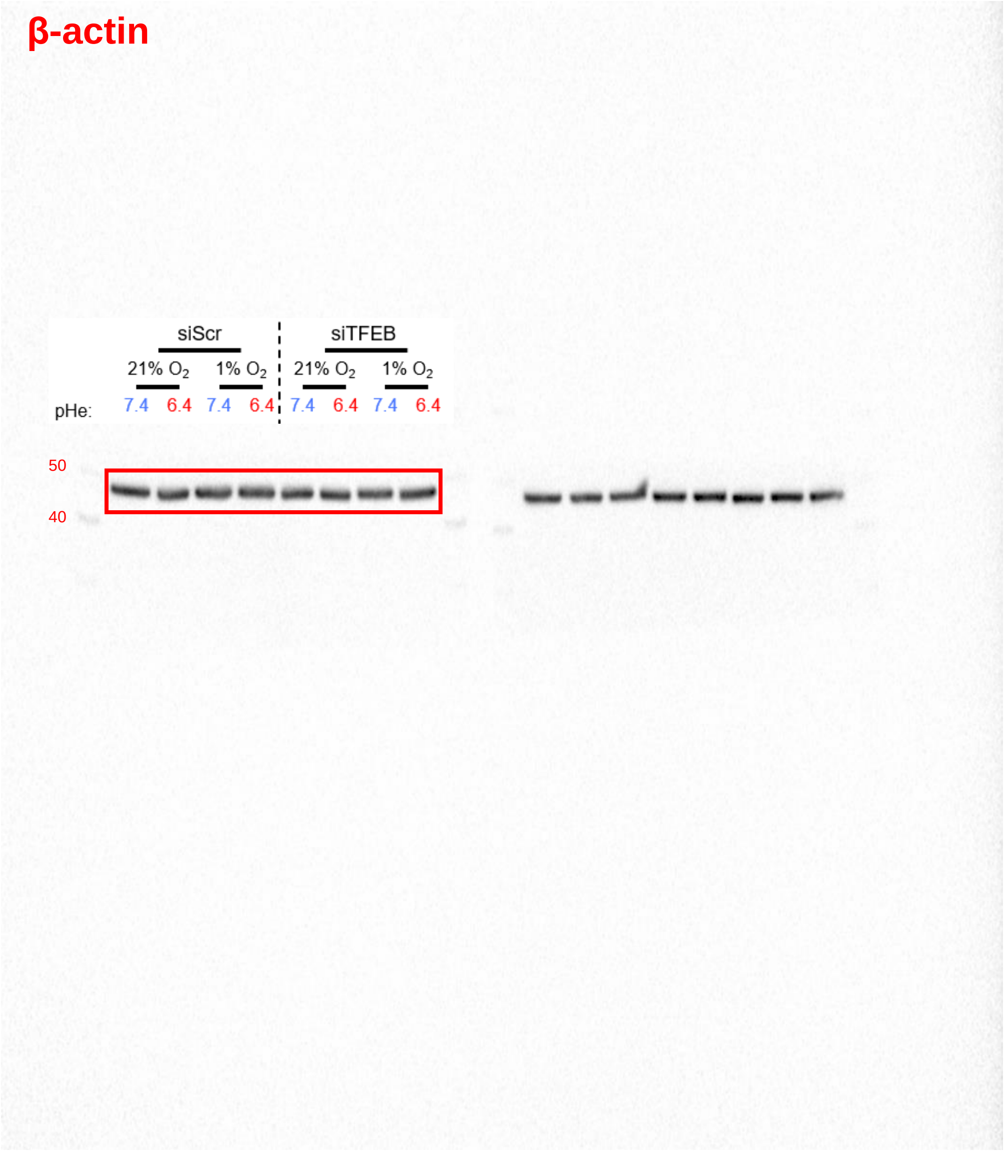

β-actin
siScr
siTFEB
21% O2
1% O2
21% O2
1% O2
pHe:
7.4
6.4
7.4
6.4
7.4
6.4
7.4
6.4
50
40

Figure 10

D

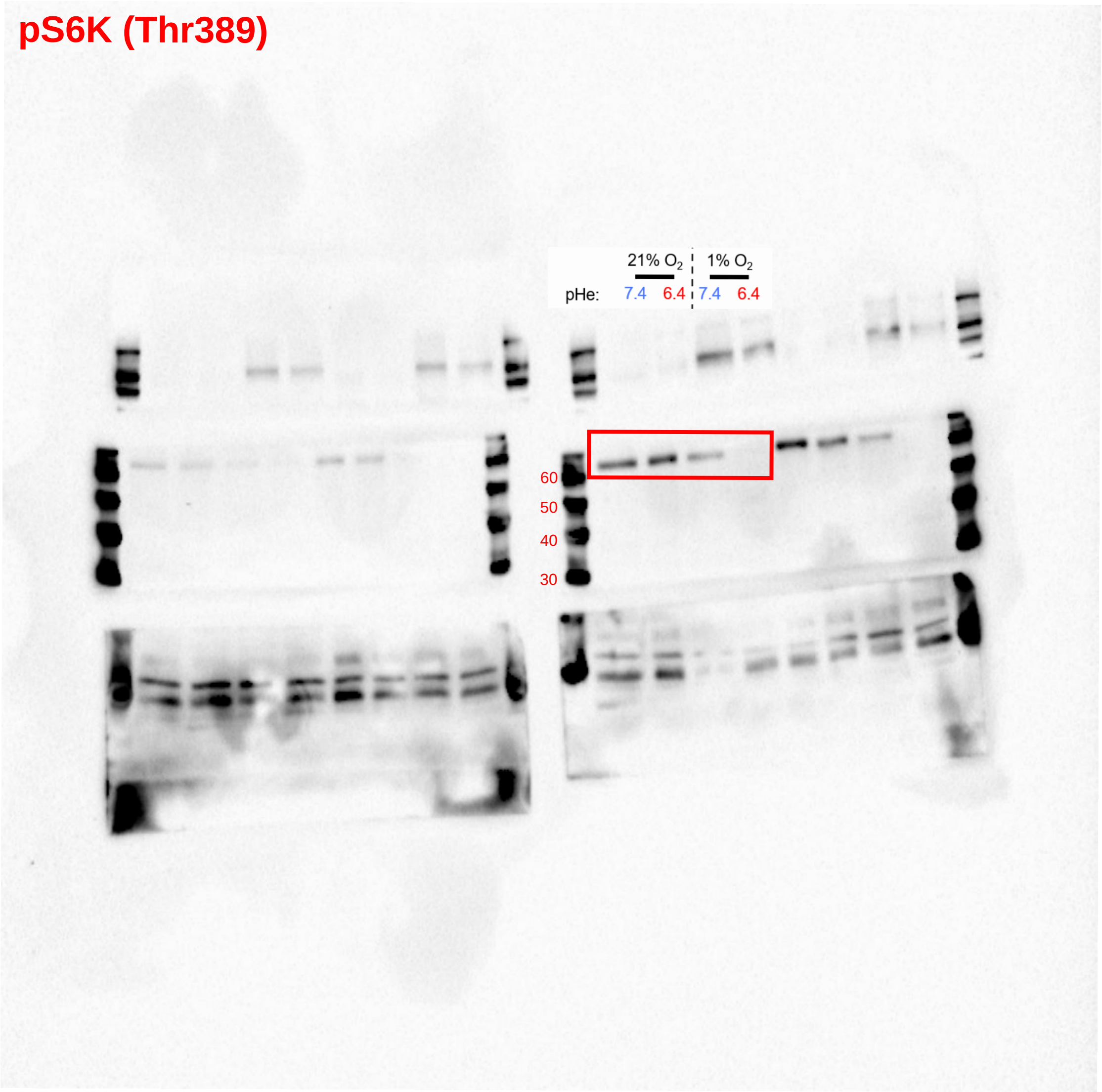

Figure 10

D

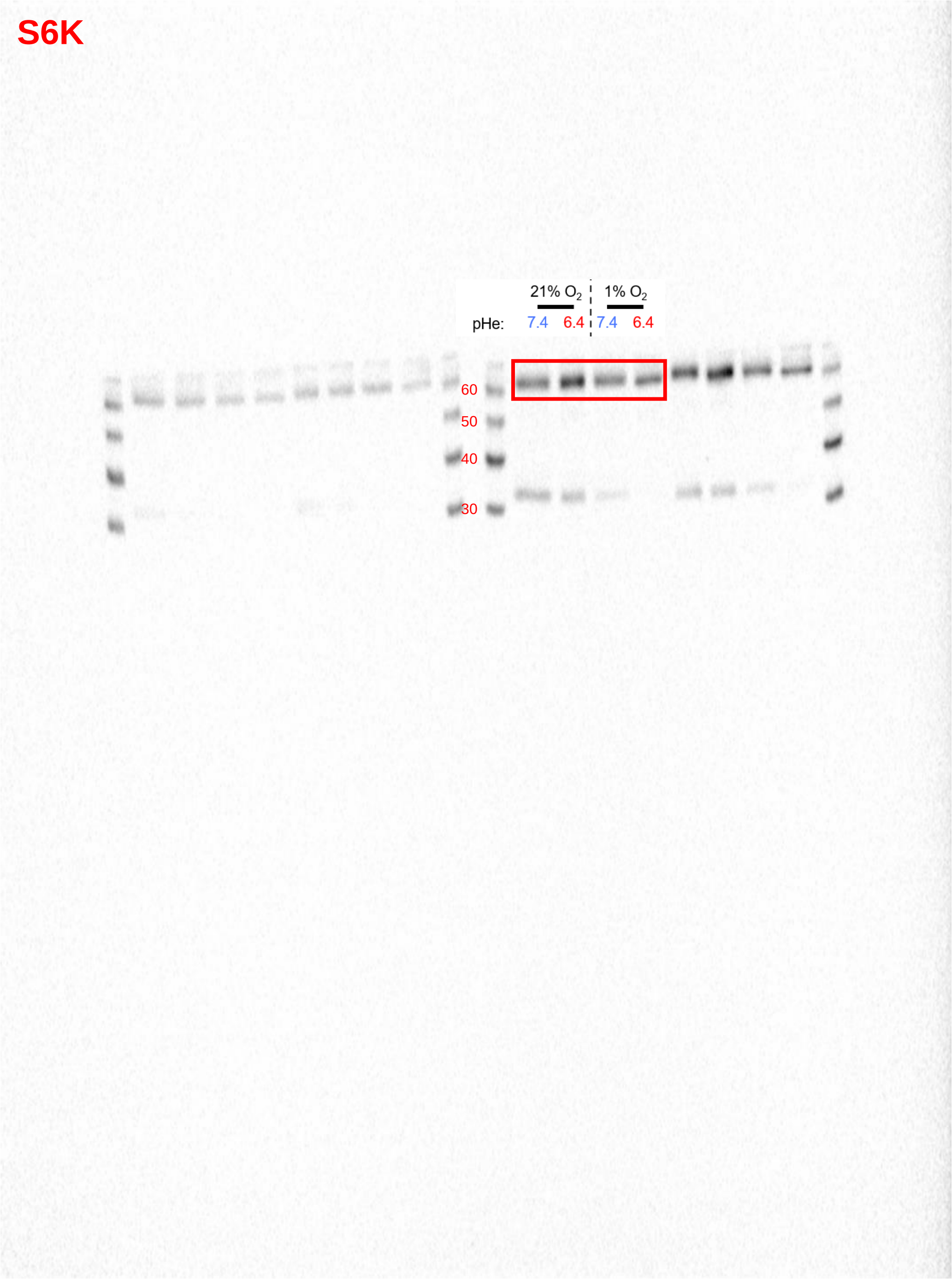

Figure 10

D

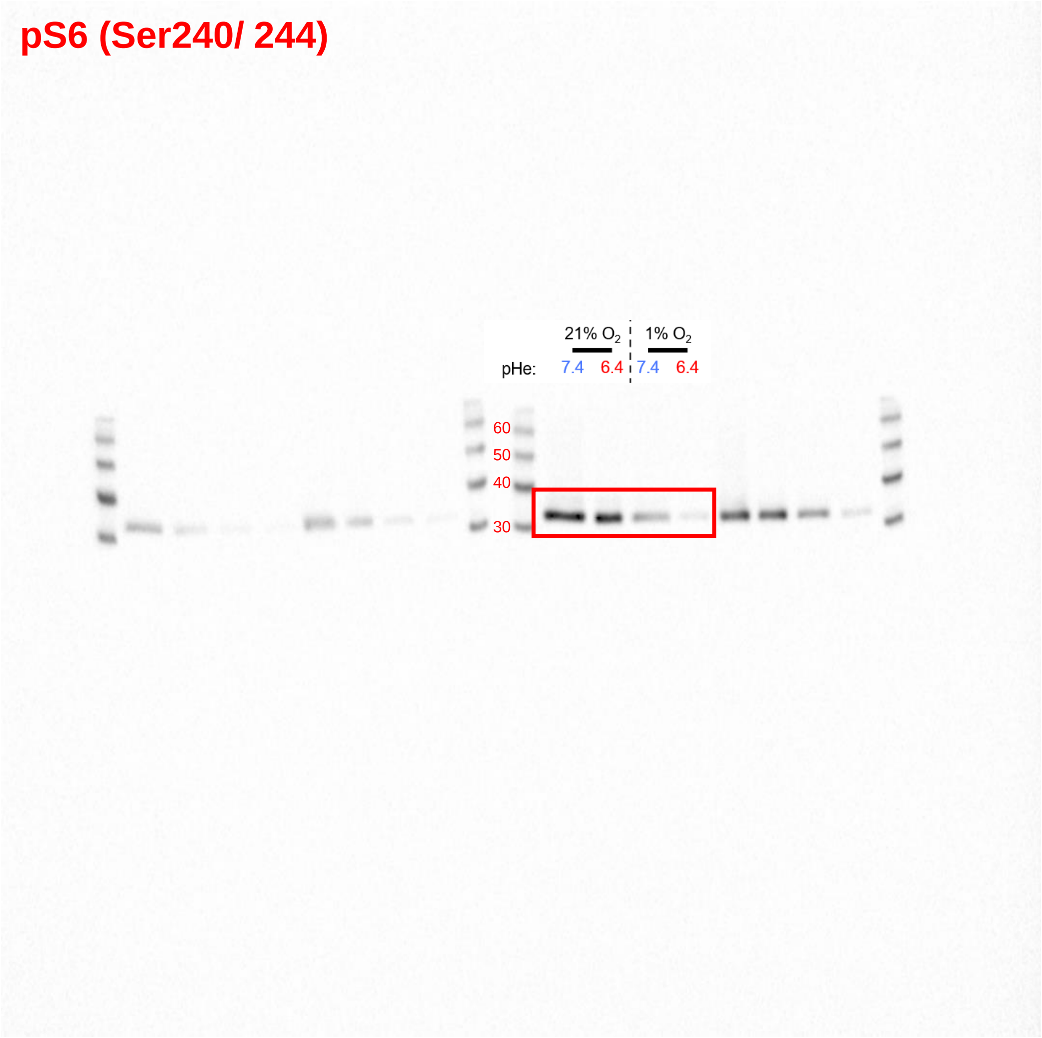

Figure 10

D

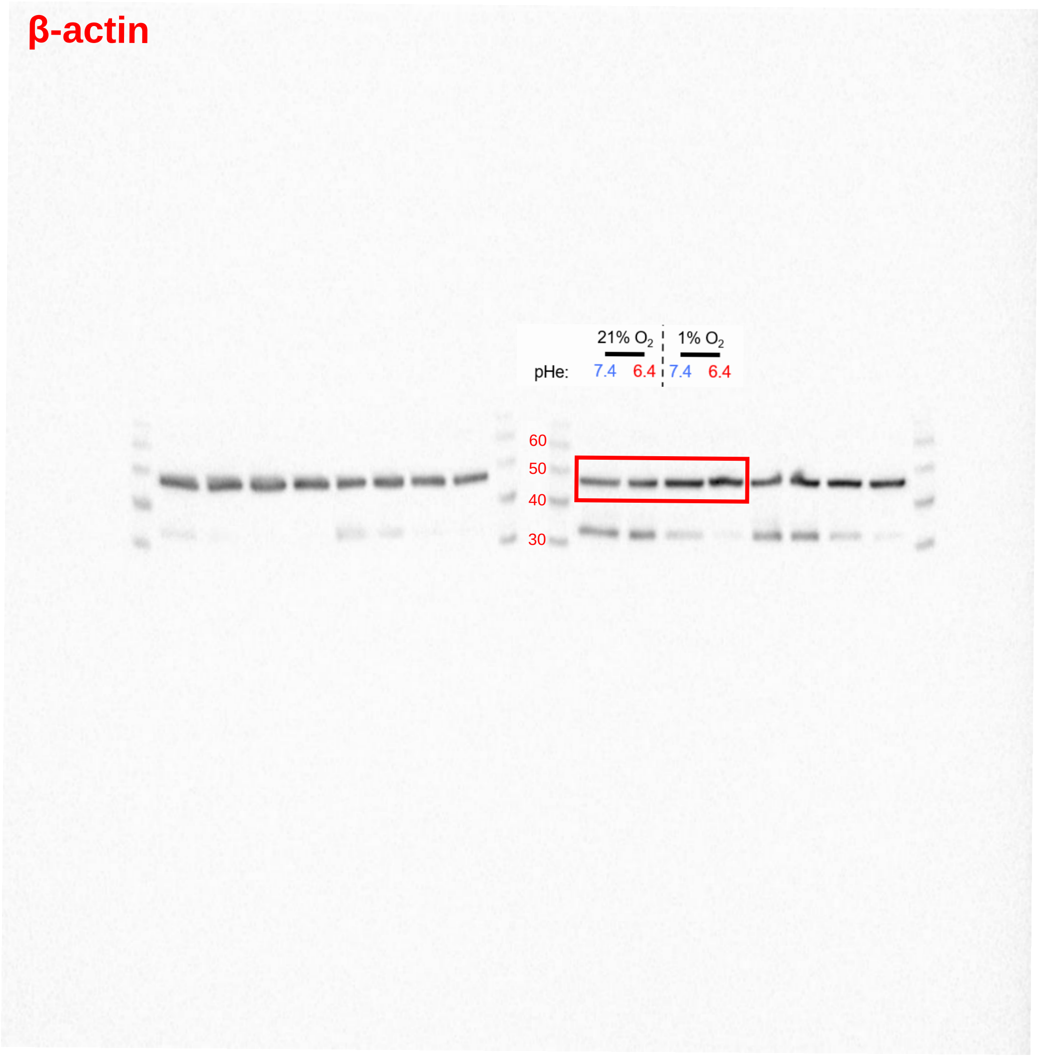

Figure 10

E

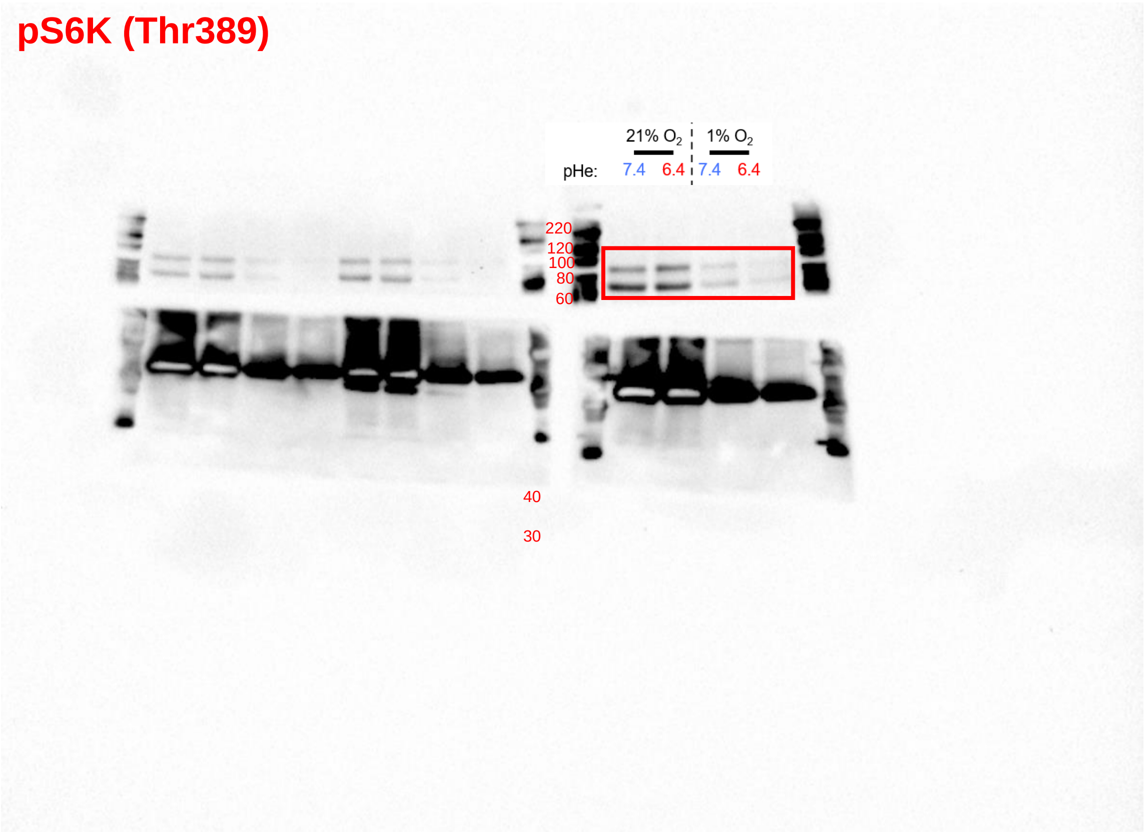

Figure 10

E

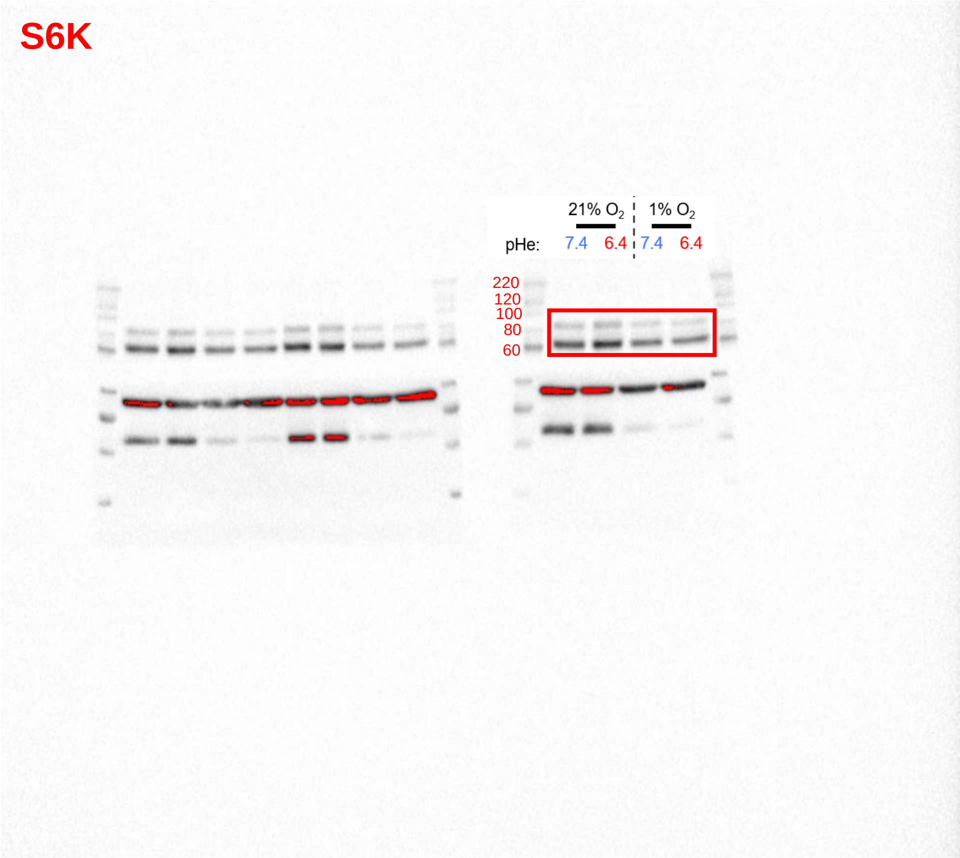

Figure 10

E

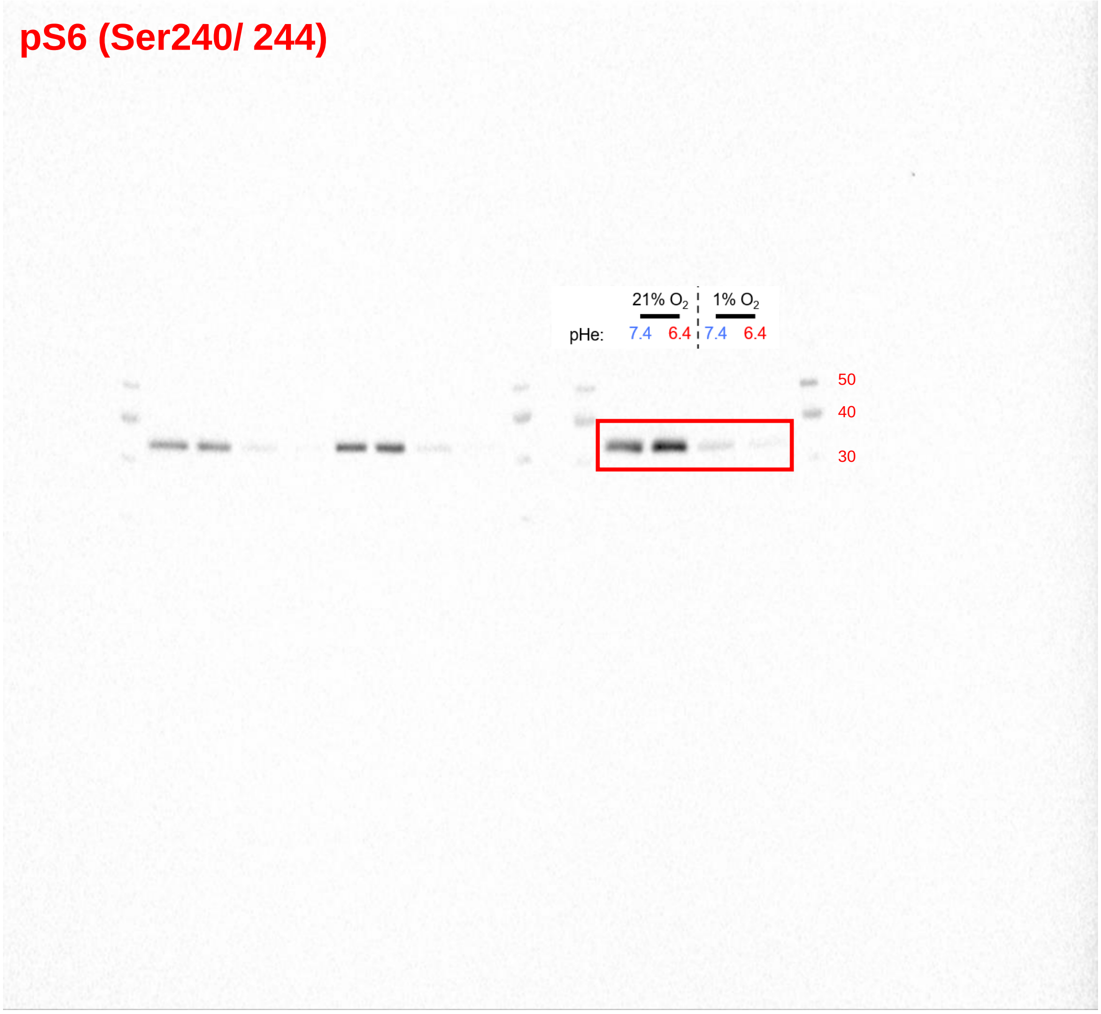

Figure 10

E

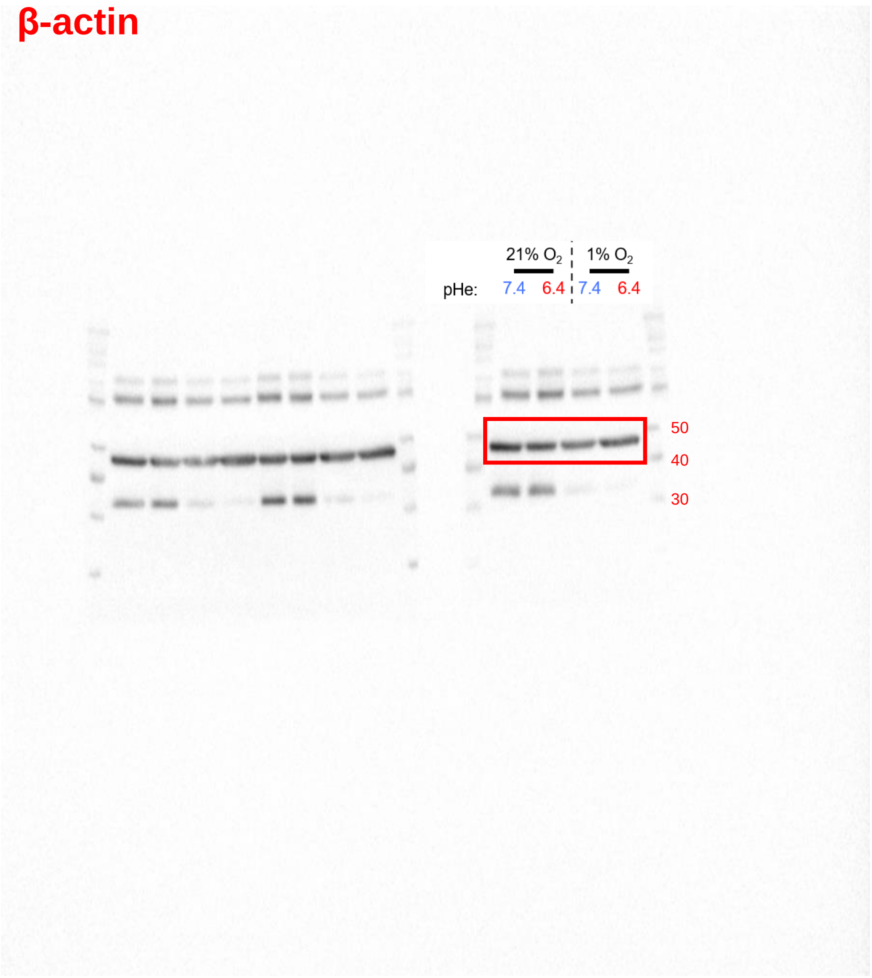

Supplement: SourceData F10 — is the source file for Fig. 10. [file jcb_202409103_sourcedataf10.pdf]
