# Supplementary material for: Acidosis attenuates the hypoxic stabilization of HIF-1α by activating lysosomal degradation
Source: J Cell Biol. 2025 Jun 24;224(8):e202409103. doi: 10.1083/jcb.202409103 (PMC12187095; doi:10.1083/jcb.202409103)

Figure S2

A

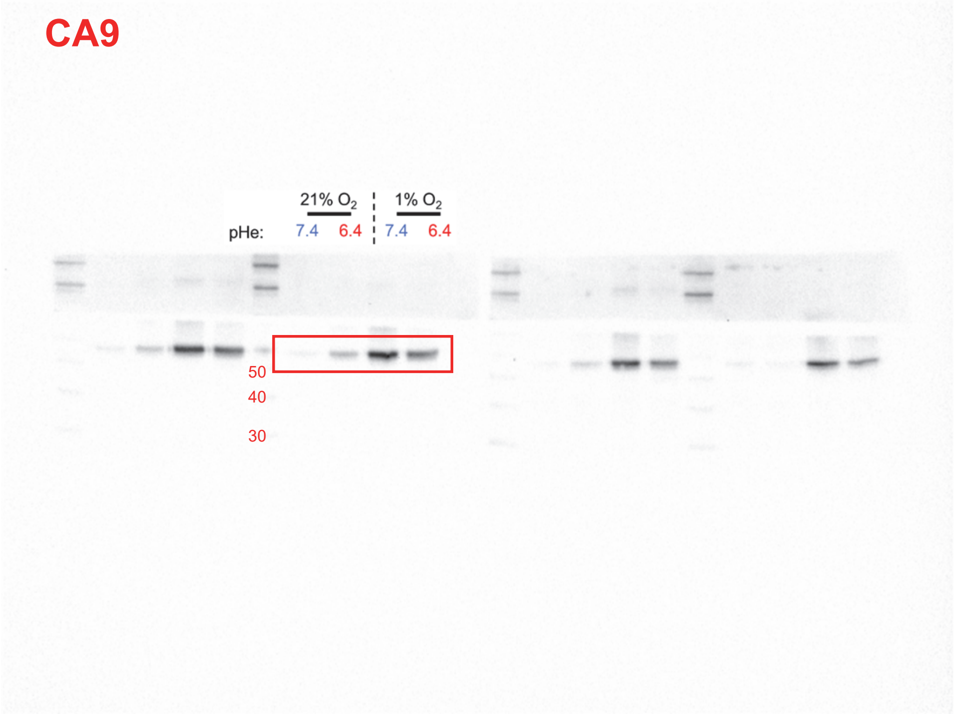
CA9
21% O_2
1% O_2
pHe: 7.4 6.4 7.4 6.4
50
40
30

A

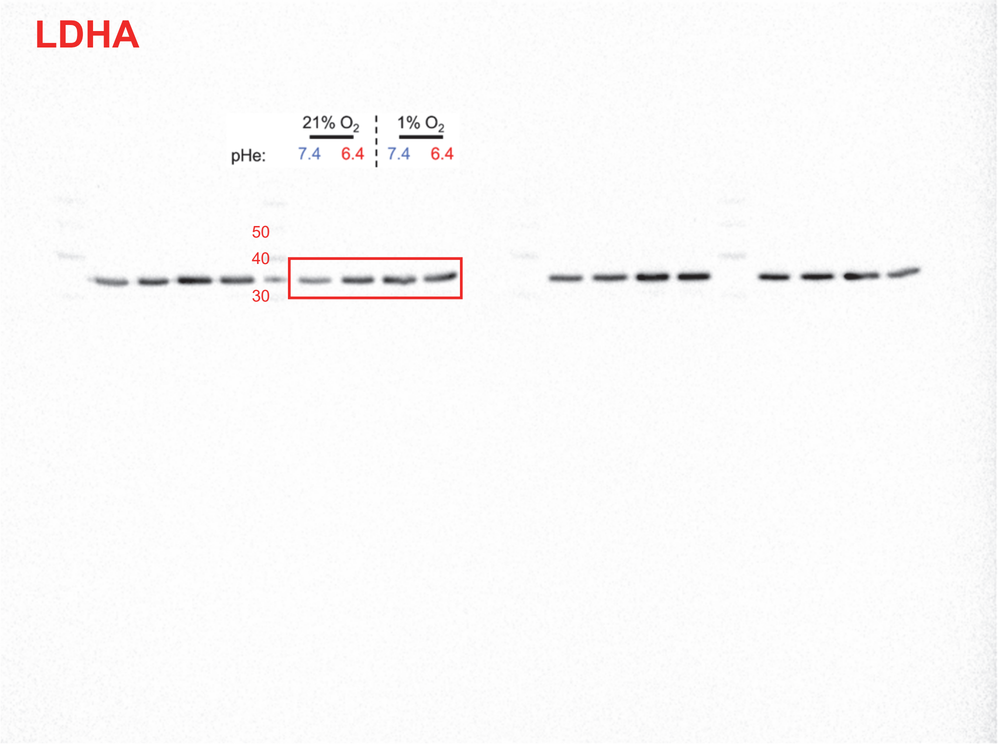
LDHA
21% O_2
1% O_2
pHe: 7.4 6.4 7.4 6.4
50
40
30

Figure S2

A

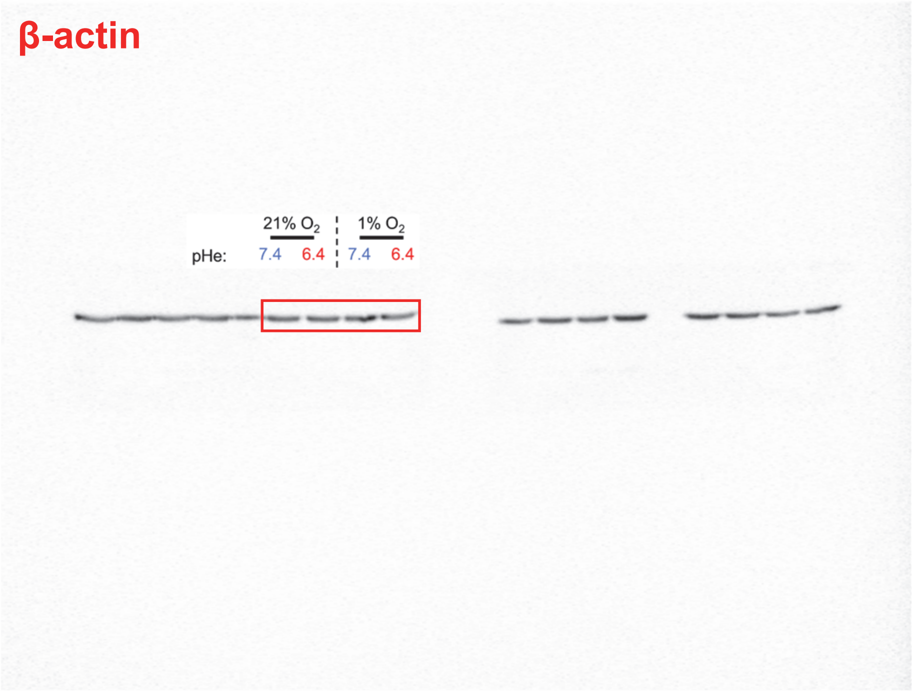

Figure S2

B

CA9

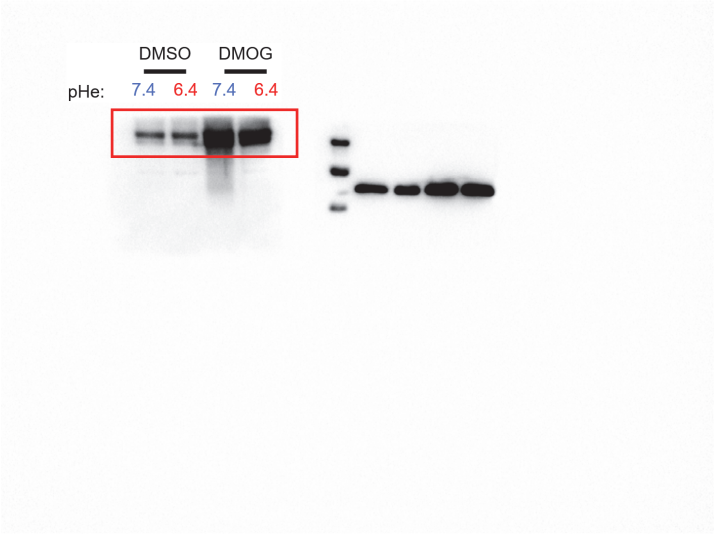

B

β-actin (left)

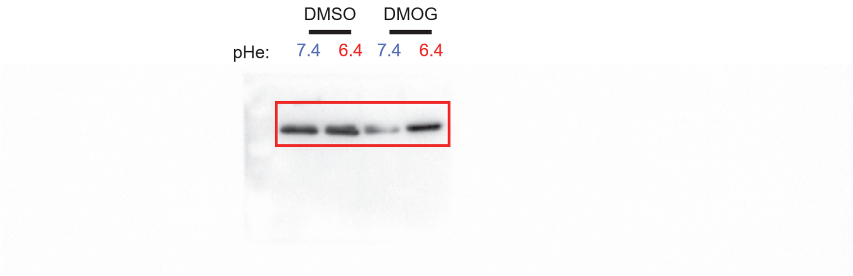

Figure S2

B

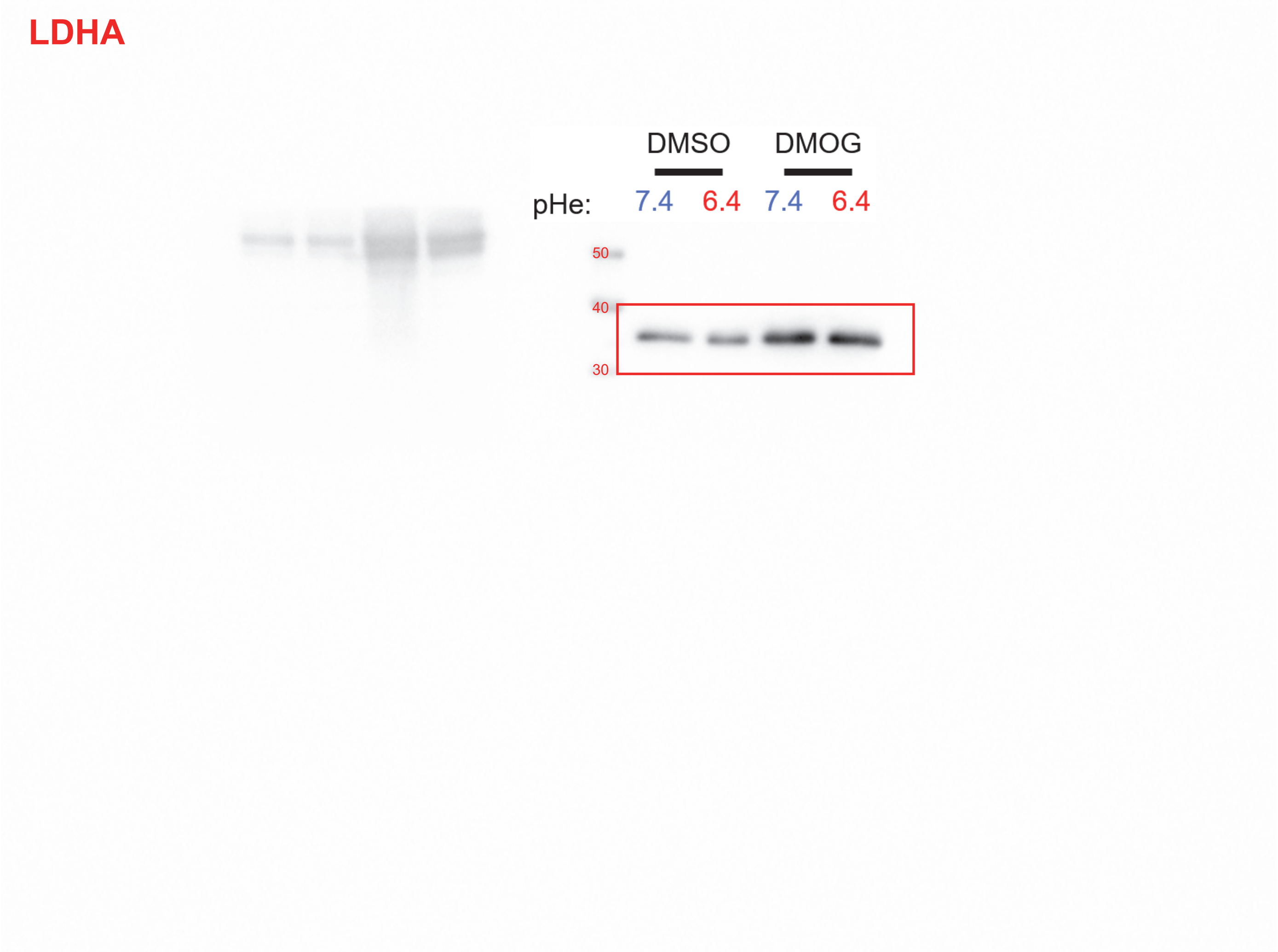

Figure S2

B

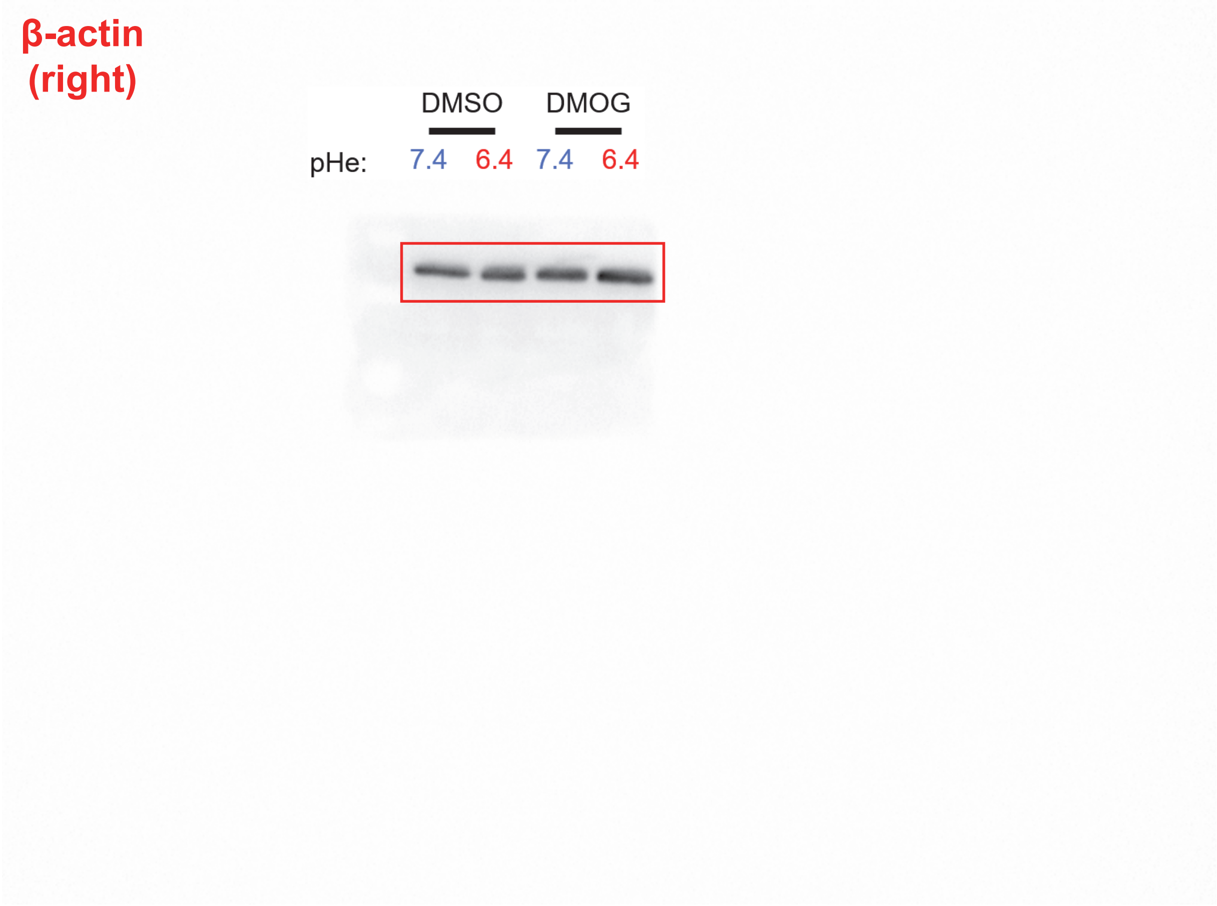

Figure S2

C

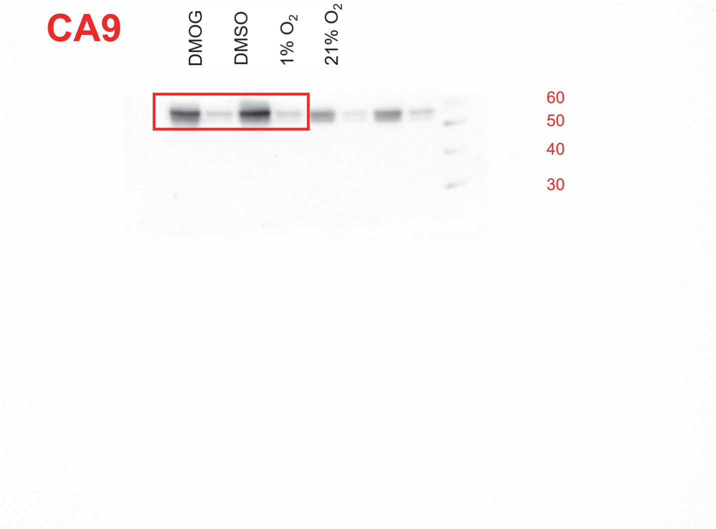

CA9
DMOG
DMSO
1% O_2
21% O_2
60
50
40
30

C

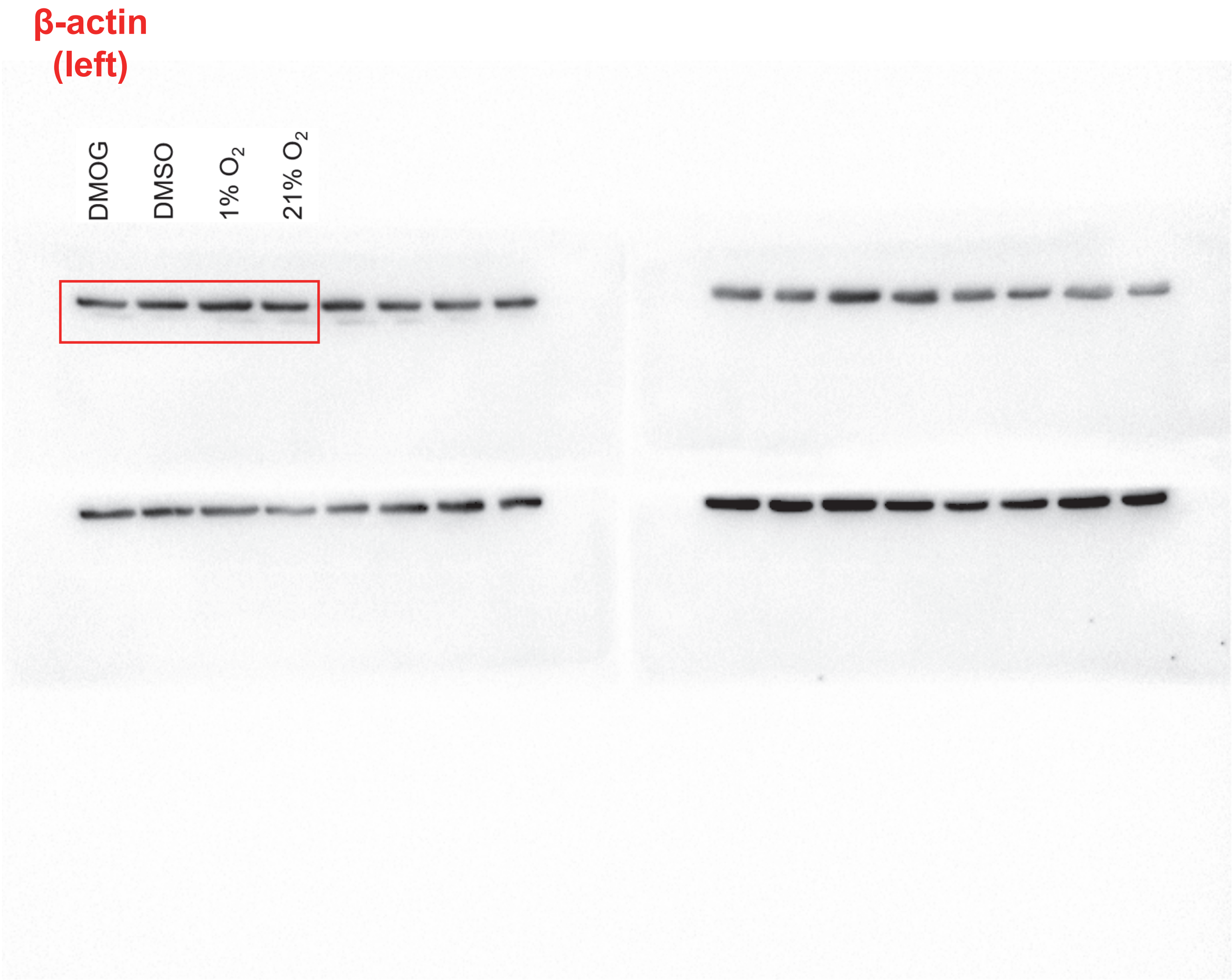

β-actin
(left)
DMOG
DMSO
1% O_2
21% O_2

Figure S2

C

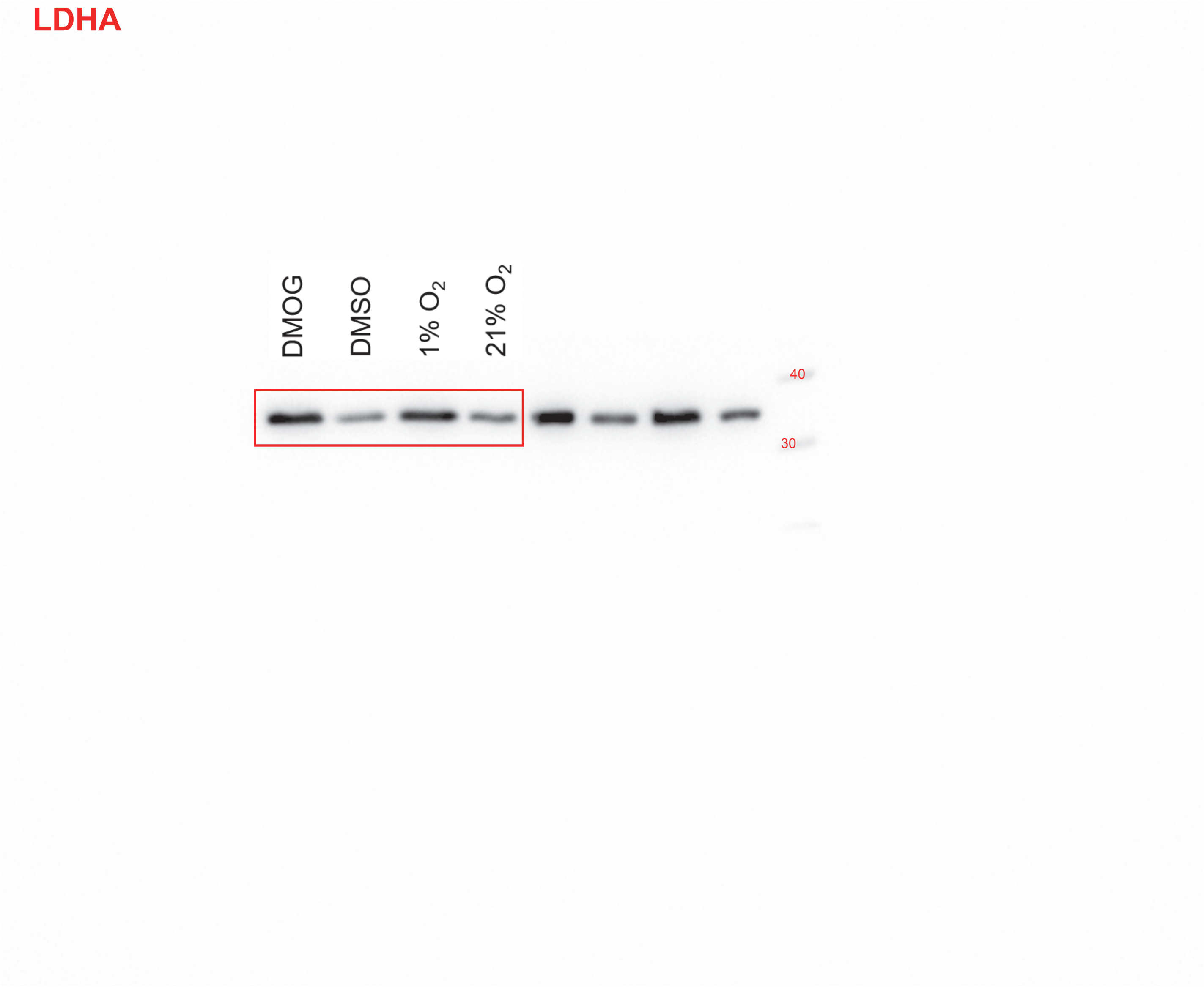

Figure S2

C

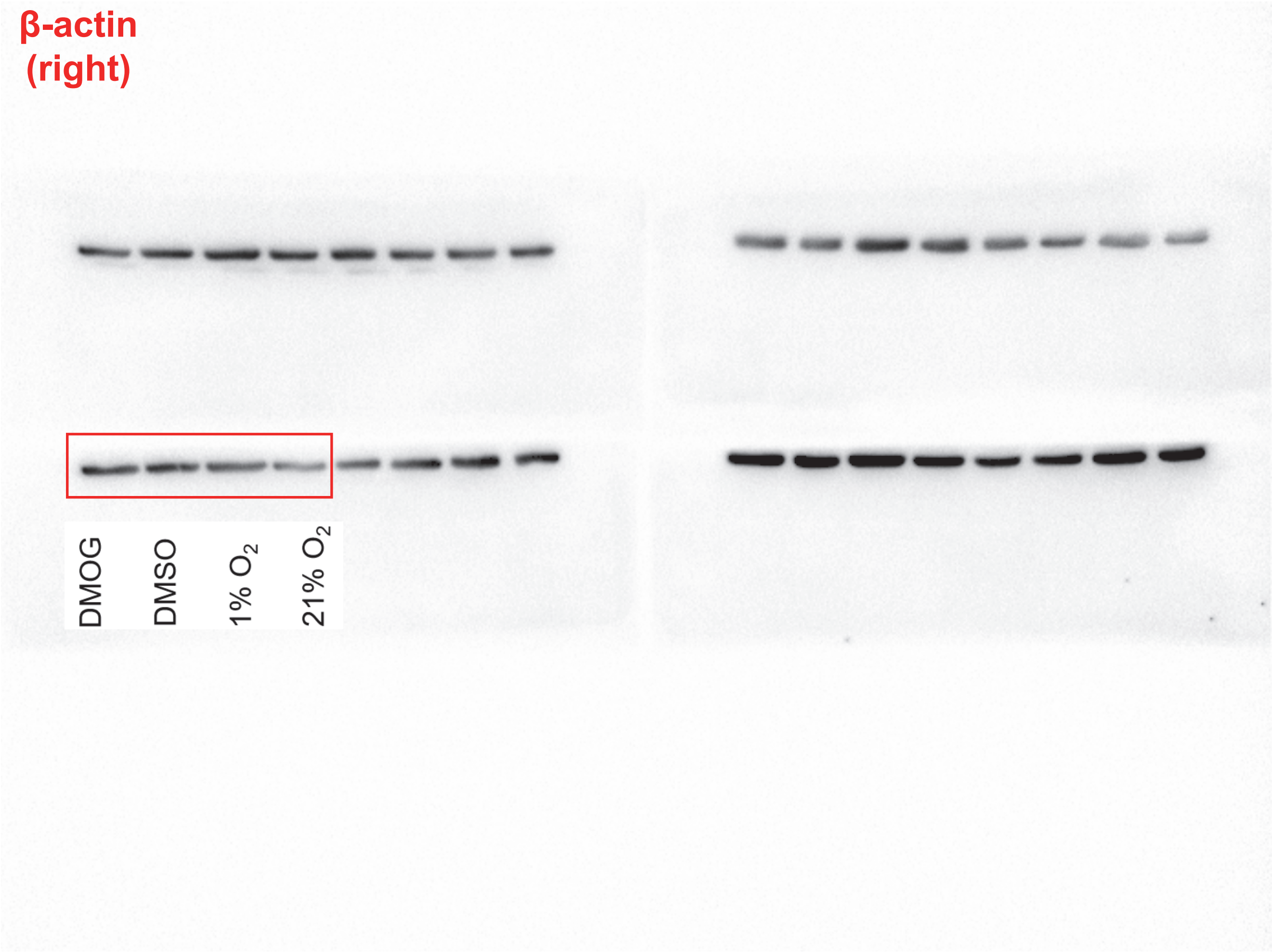

Figure S2

E

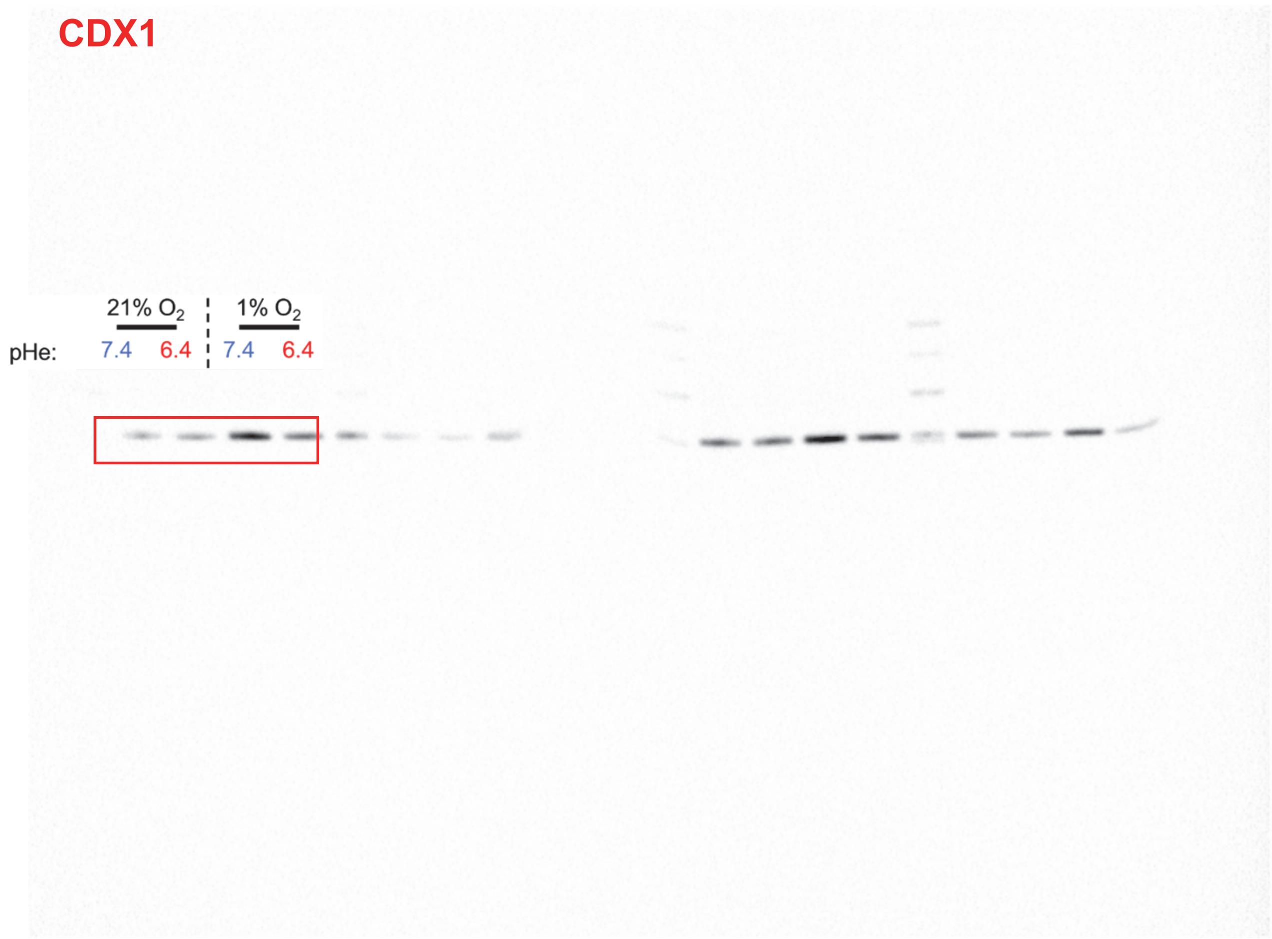

E

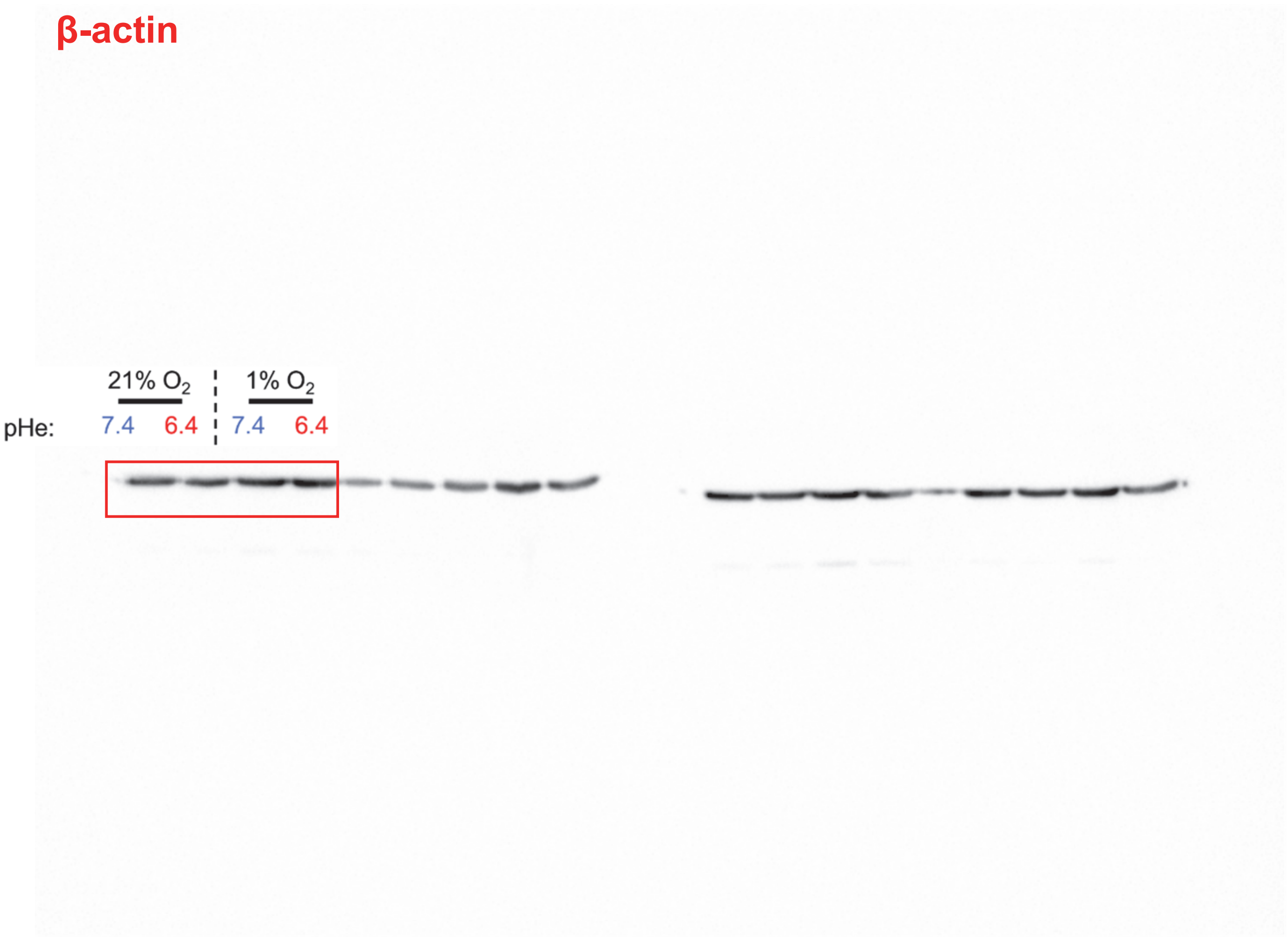

Supplement: SourceData FS2 — is the source file for Fig. S2. [file jcb_202409103_sourcedatafs2.pdf]
