# Supplementary material for: Acidosis attenuates the hypoxic stabilization of HIF-1α by activating lysosomal degradation
Source: J Cell Biol. 2025 Jun 24;224(8):e202409103. doi: 10.1083/jcb.202409103 (PMC12187095; doi:10.1083/jcb.202409103)

**Figure S3**

A

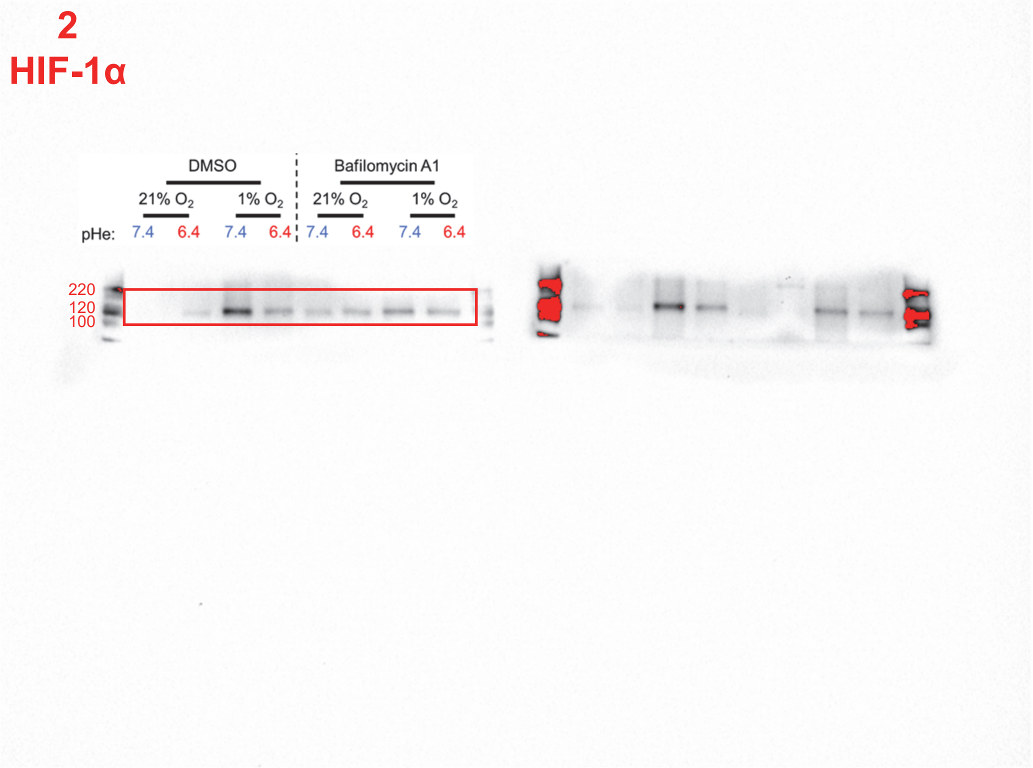
2
HIF-1α
DMSO
Bafilomycin A1
21% $O_2$
1% $O_2$
21% $O_2$
1% $O_2$
pHe: 7.4 6.4 7.4 6.4 7.4 6.4 7.4 6.4
220
120
100

A

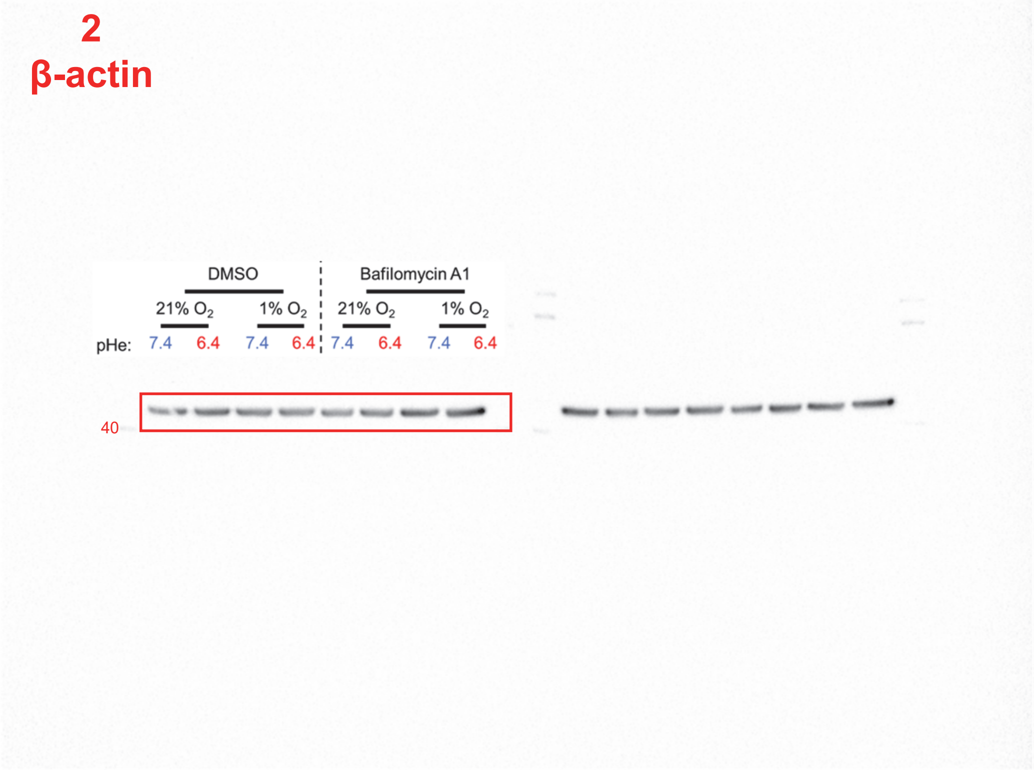
2
β-actin
DMSO
Bafilomycin A1
21% $O_2$
1% $O_2$
21% $O_2$
1% $O_2$
pHe: 7.4 6.4 7.4 6.4 7.4 6.4 7.4 6.4
40

# Figure S3

A

3
HIF-1α

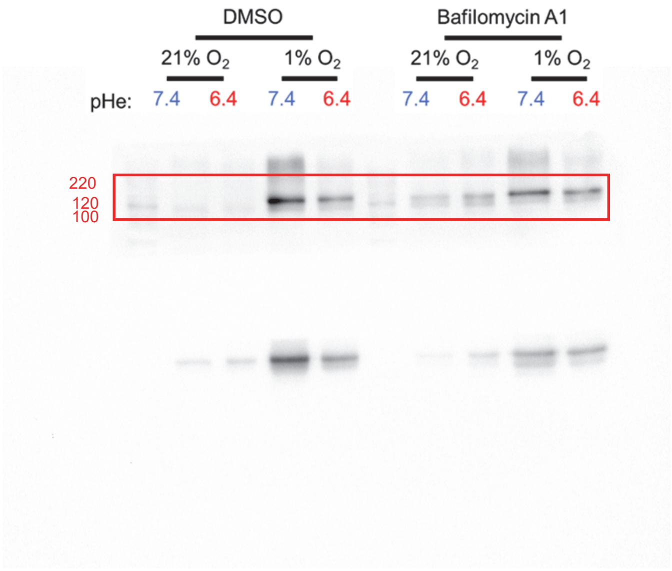


A

3
β-actin

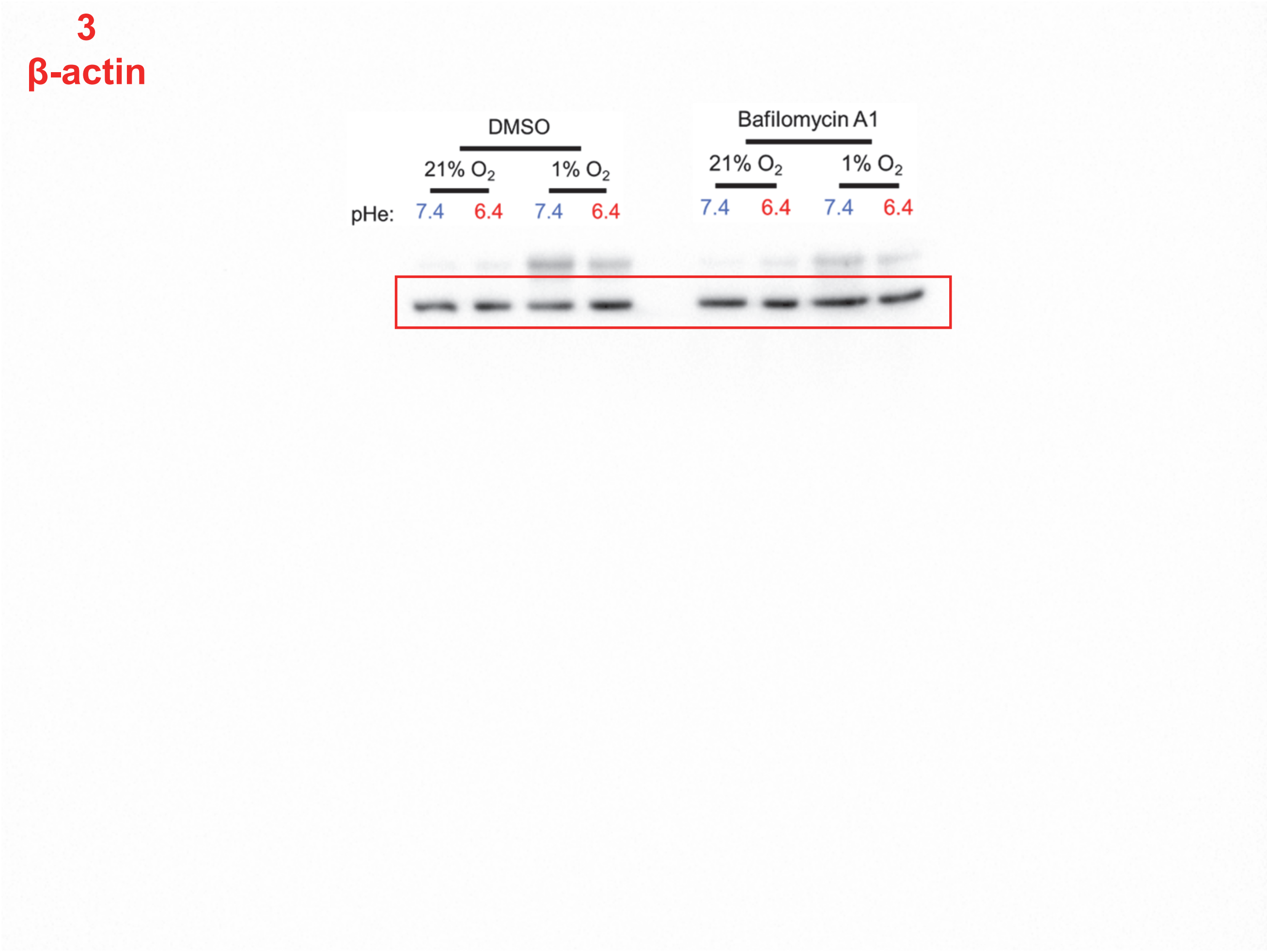

**Figure S3**

A

**4**
**HIF-1α**

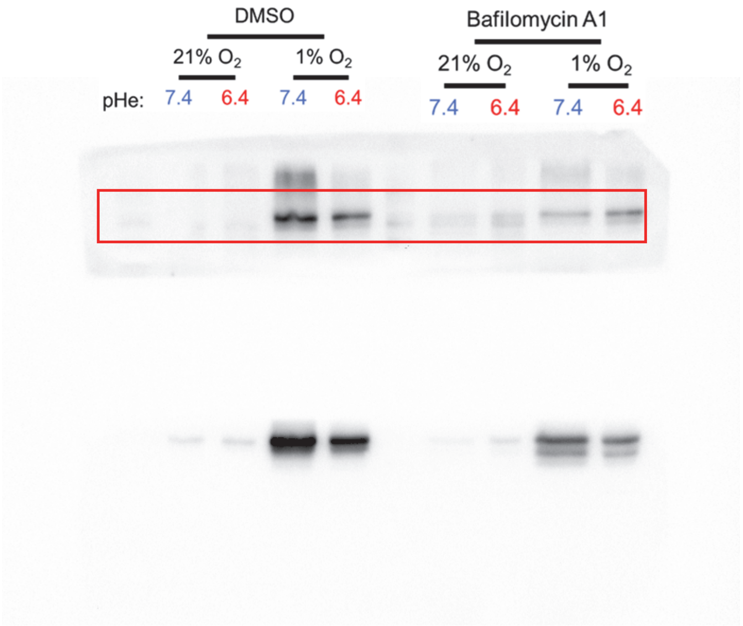


A

**4**
**β-actin**

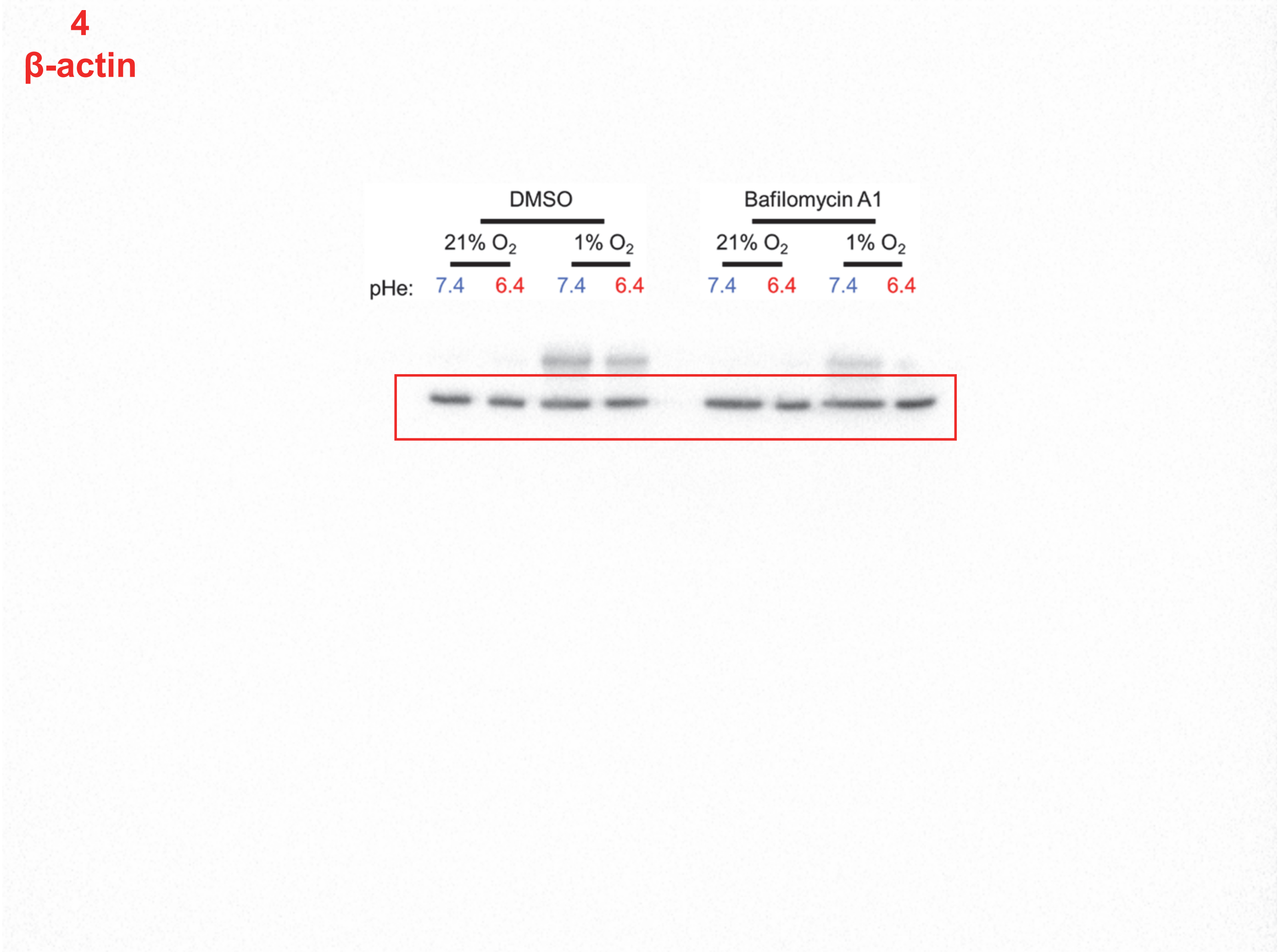

**Figure S3**

B

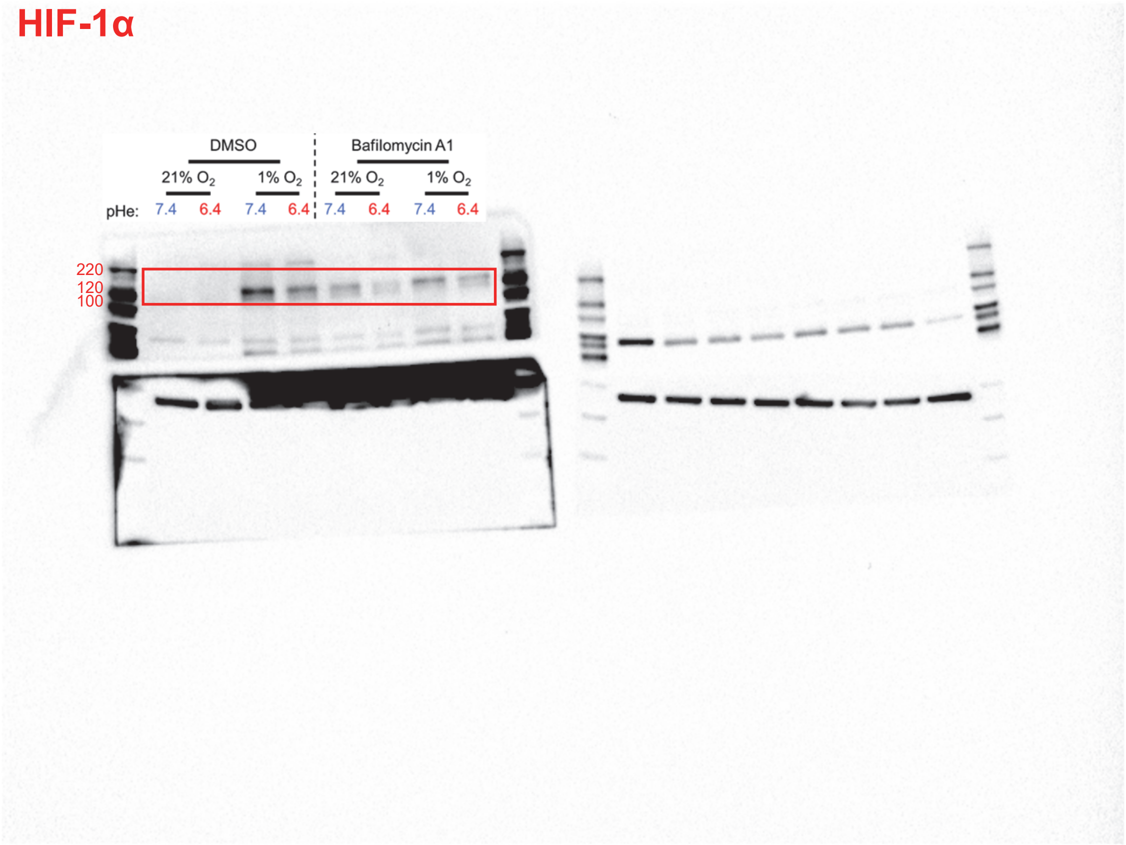
HIF-1α
DMSO
Bafilomycin A1
21% O2
1% O2
21% O2
1% O2
pHe: 7.4 6.4 7.4 6.4 7.4 6.4 7.4 6.4
220
120
100

B

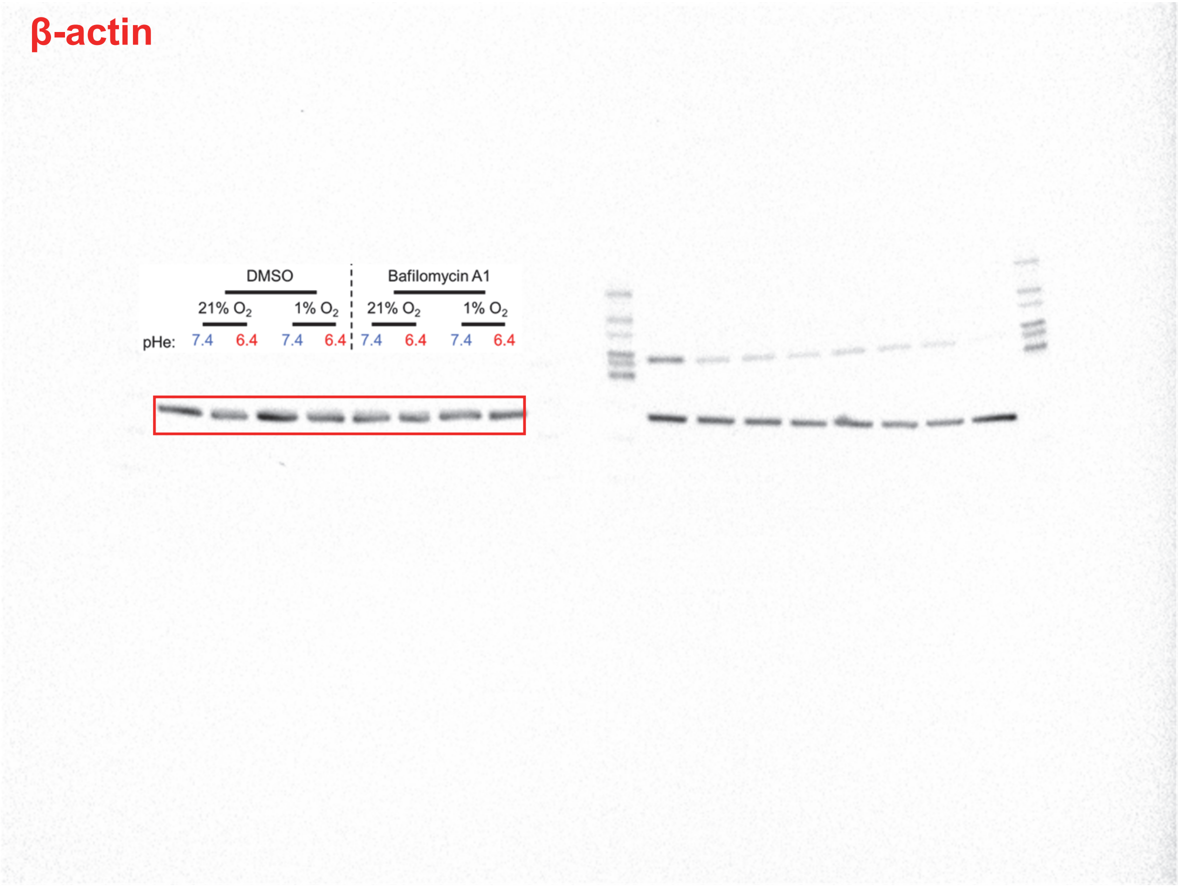
β-actin
DMSO
Bafilomycin A1
21% O2
1% O2
21% O2
1% O2
pHe: 7.4 6.4 7.4 6.4 7.4 6.4 7.4 6.4

**Figure S3**

C

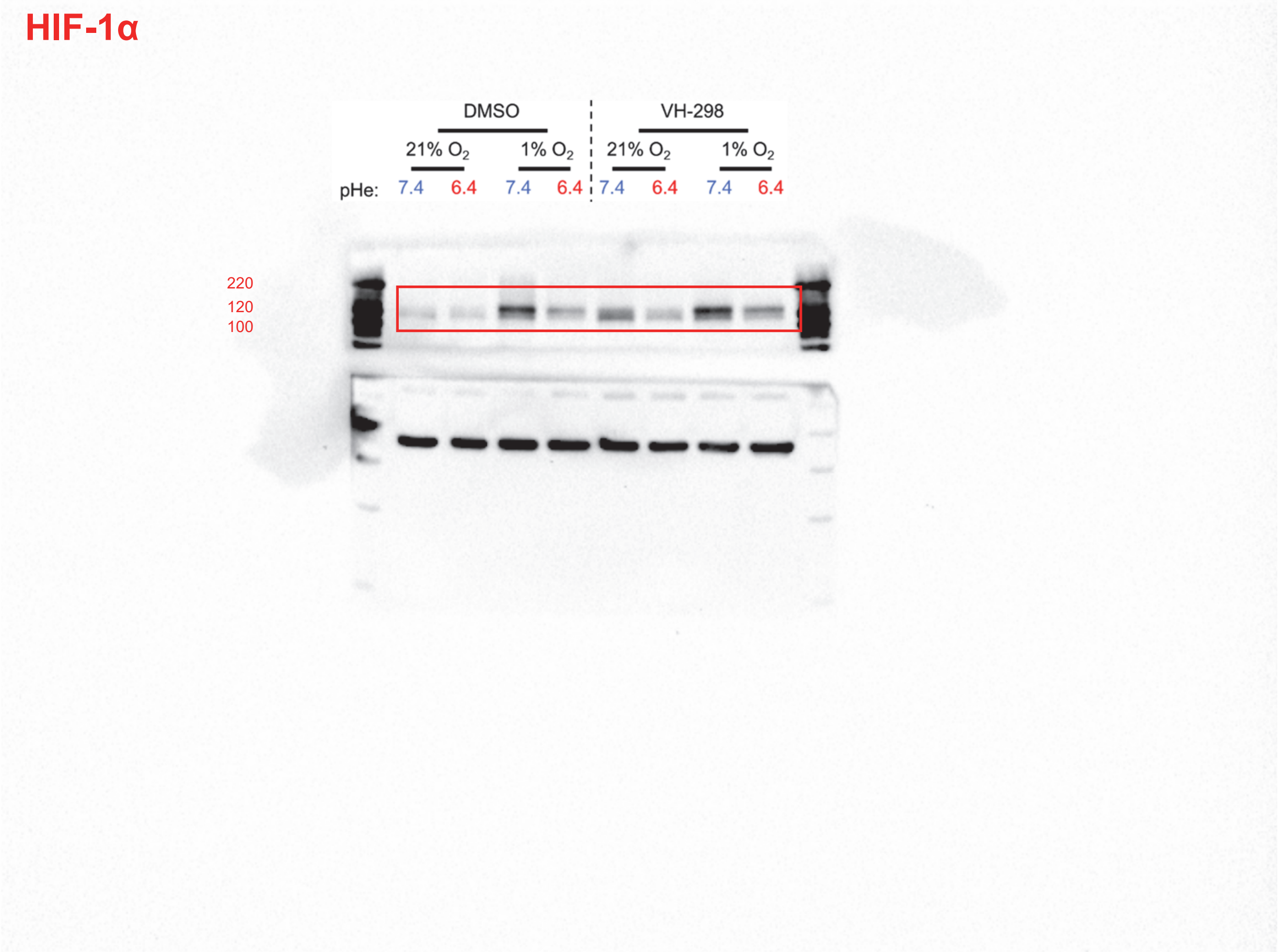
HIF-1α
DMSO
VH-298
21% $O_2$
1% $O_2$
21% $O_2$
1% $O_2$
pHe: 7.4 6.4 7.4 6.4 7.4 6.4 7.4 6.4
220
120
100

## Figure S3

C

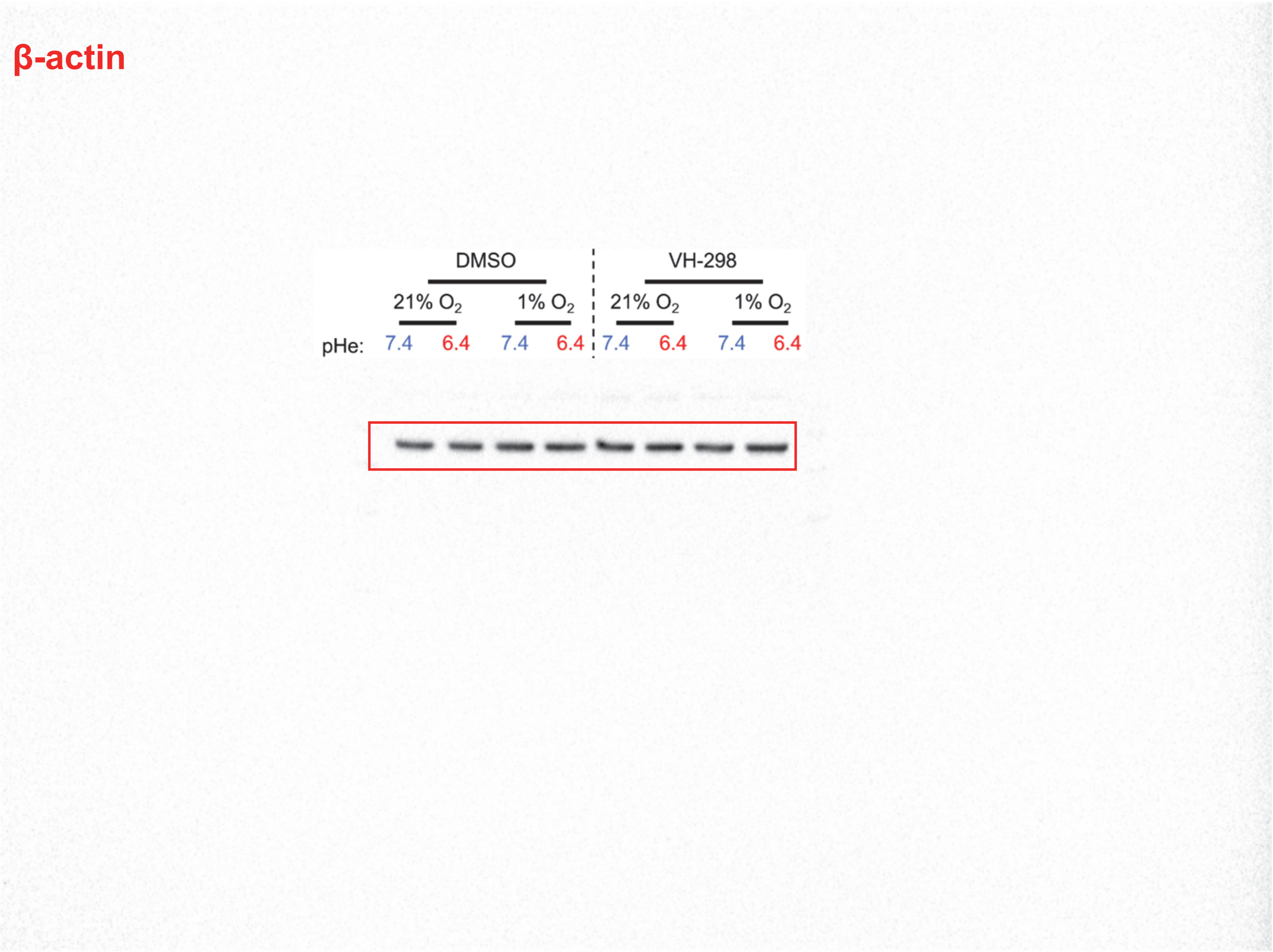

Supplement: SourceData FS3 — is the source file for Fig. S3. [file jcb_202409103_sourcedatafs3.pdf]
