# Supplementary material for: Acidosis attenuates the hypoxic stabilization of HIF-1α by activating lysosomal degradation
Source: J Cell Biol. 2025 Jun 24;224(8):e202409103. doi: 10.1083/jcb.202409103 (PMC12187095; doi:10.1083/jcb.202409103)

Figure S5

A

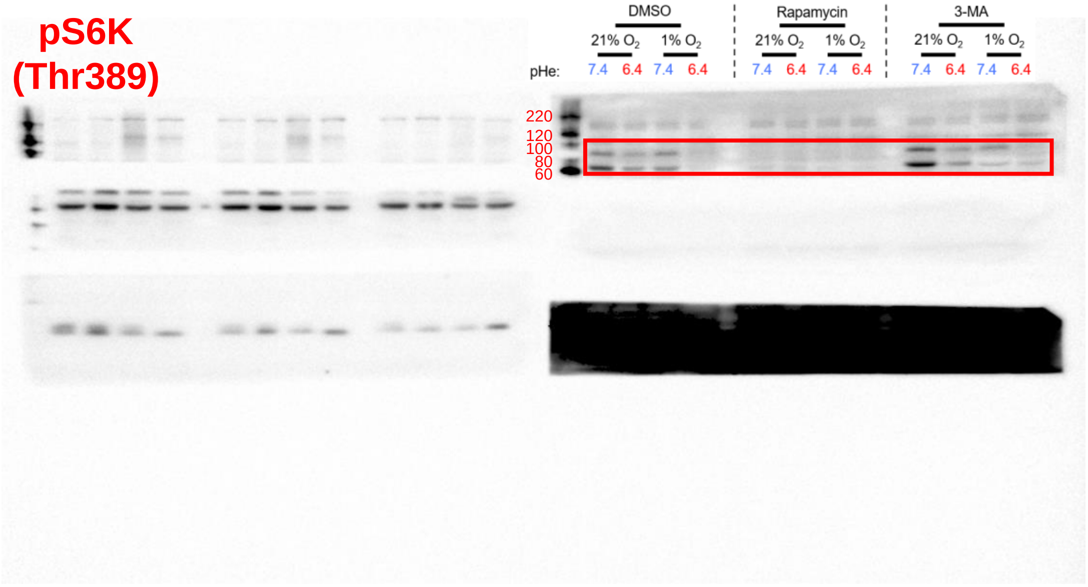
pS6K
(Thr389)
DMSO
Rapamycin
3-MA
21% O_2
1% O_2
pHe:
7.4
6.4
220
120
100
80
60

Figure S5

A

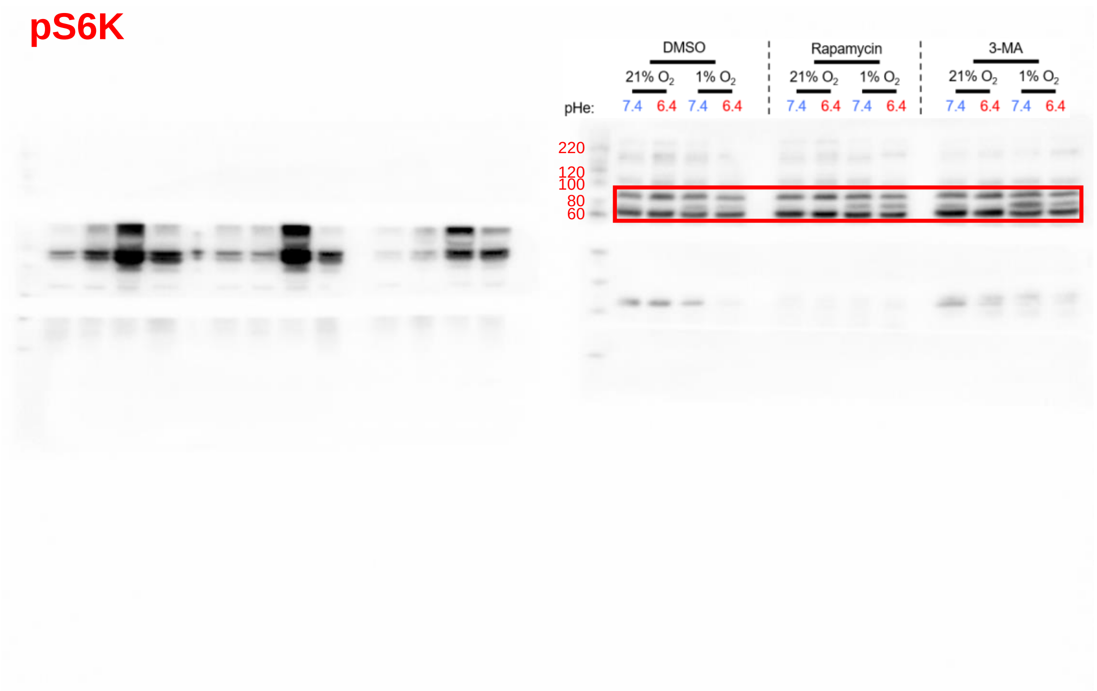

Figure S5

A

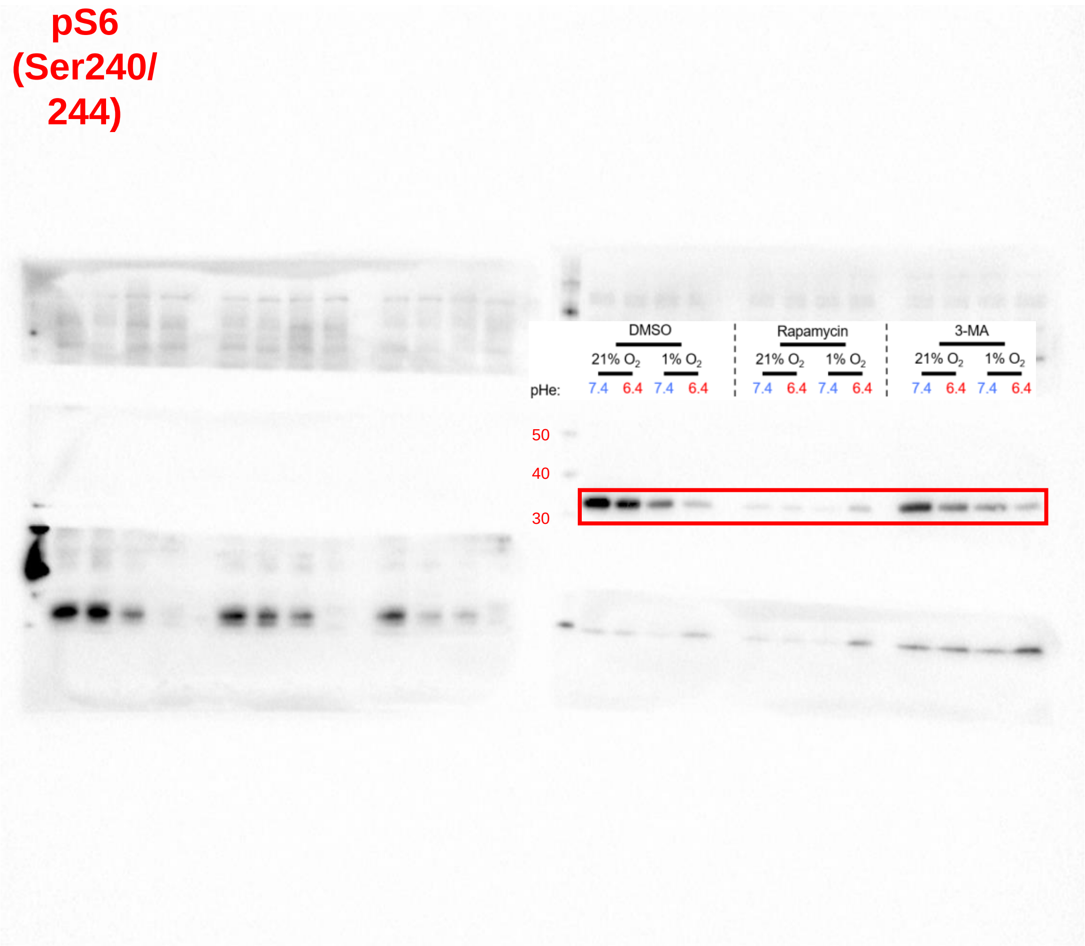

Figure S5

A

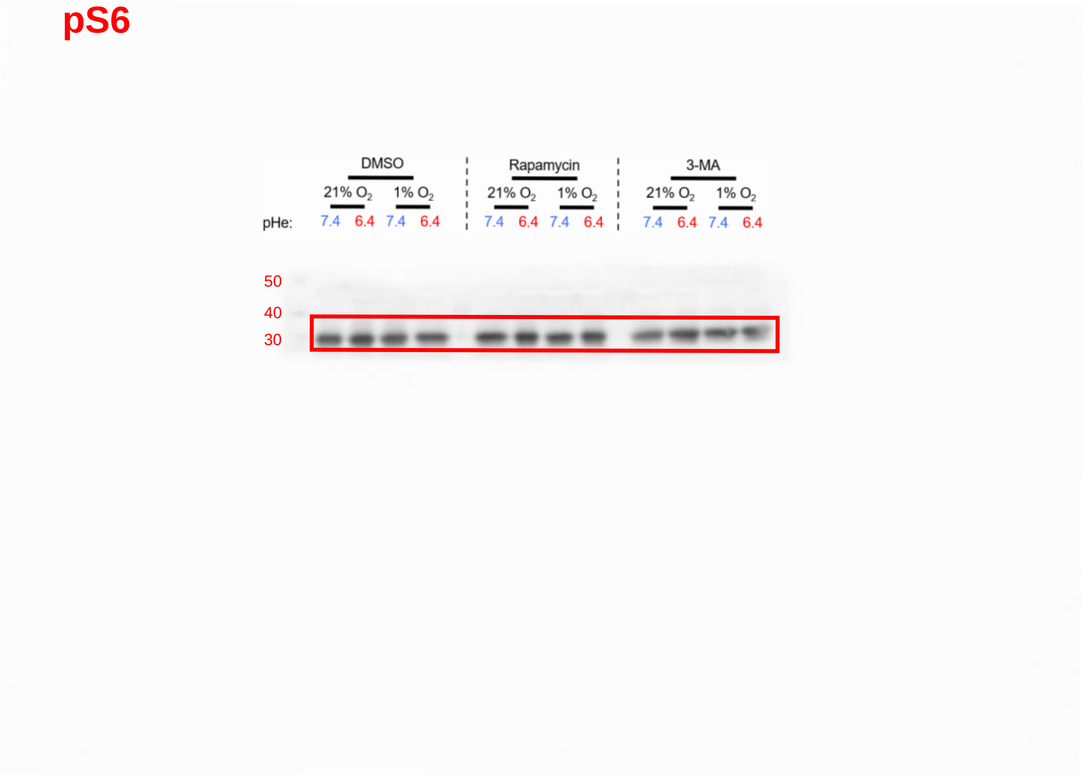

pS6
DMSO
Rapamycin
3-MA
21% O2
1% O2
21% O2
1% O2
21% O2
1% O2
pHe:
7.4 6.4 7.4 6.4
7.4 6.4 7.4 6.4
7.4 6.4 7.4 6.4
50
40
30

Figure S5

A

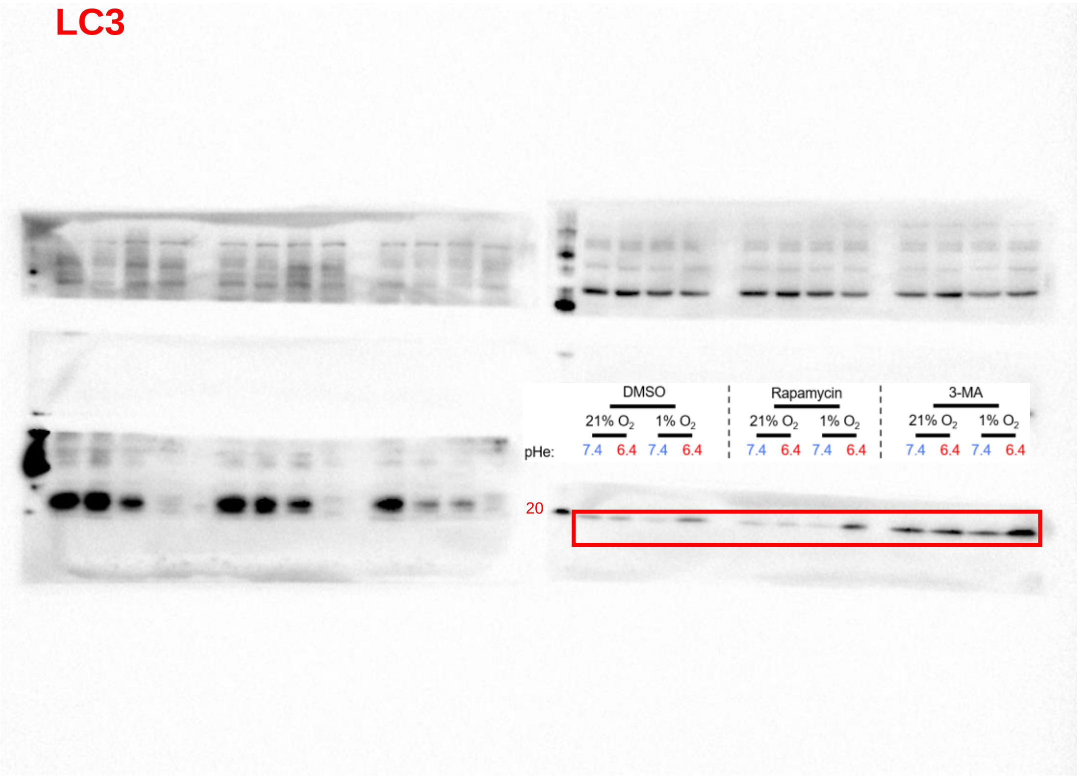

LC3
DMSO
Rapamycin
3-MA
21% O2
1% O2
21% O2
1% O2
21% O2
1% O2
pHe:
7.4
6.4
7.4
6.4
7.4
6.4
7.4
6.4
7.4
6.4
7.4
6.4
20

Figure S5

A

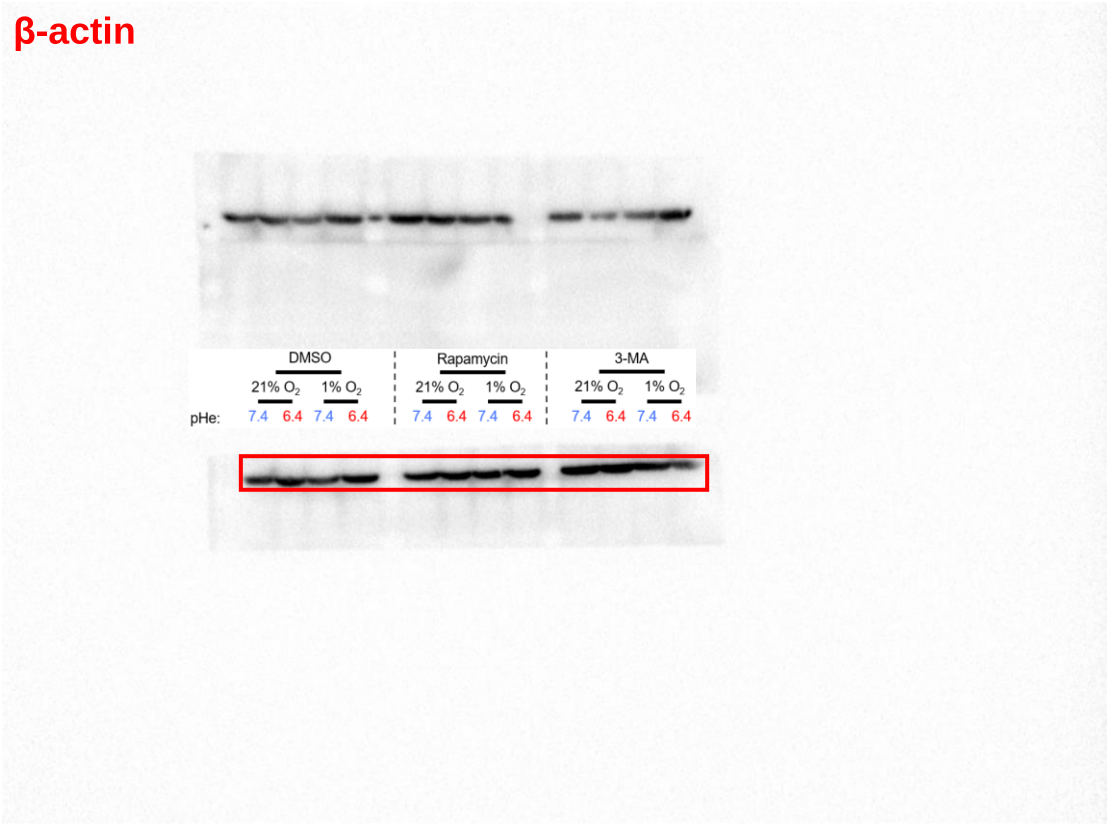
β-actin
DMSO
Rapamycin
3-MA
21% O2
1% O2
21% O2
1% O2
21% O2
1% O2
pHe:
7.4 6.4 7.4 6.4
7.4 6.4 7.4 6.4
7.4 6.4 7.4 6.4

Figure S5

B

HIF-1α

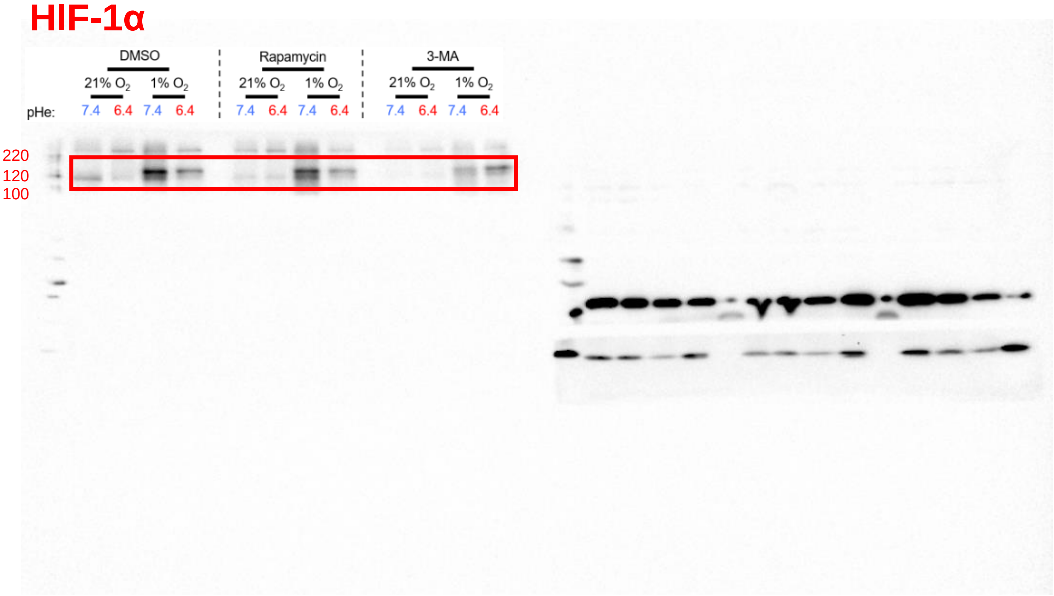

Figure S5

B

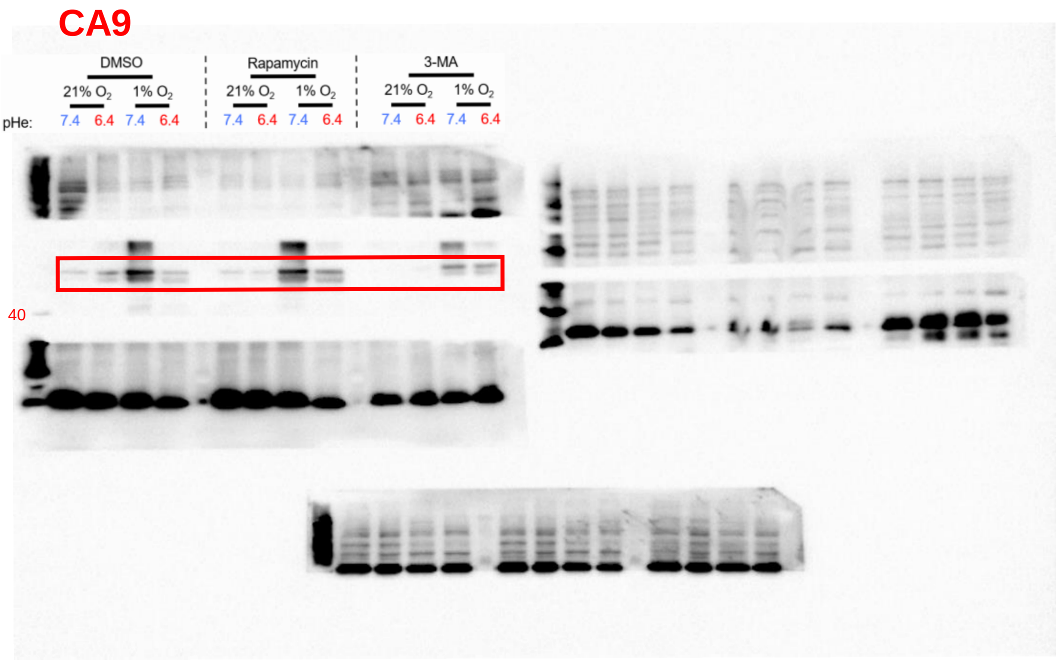

Figure S5

B

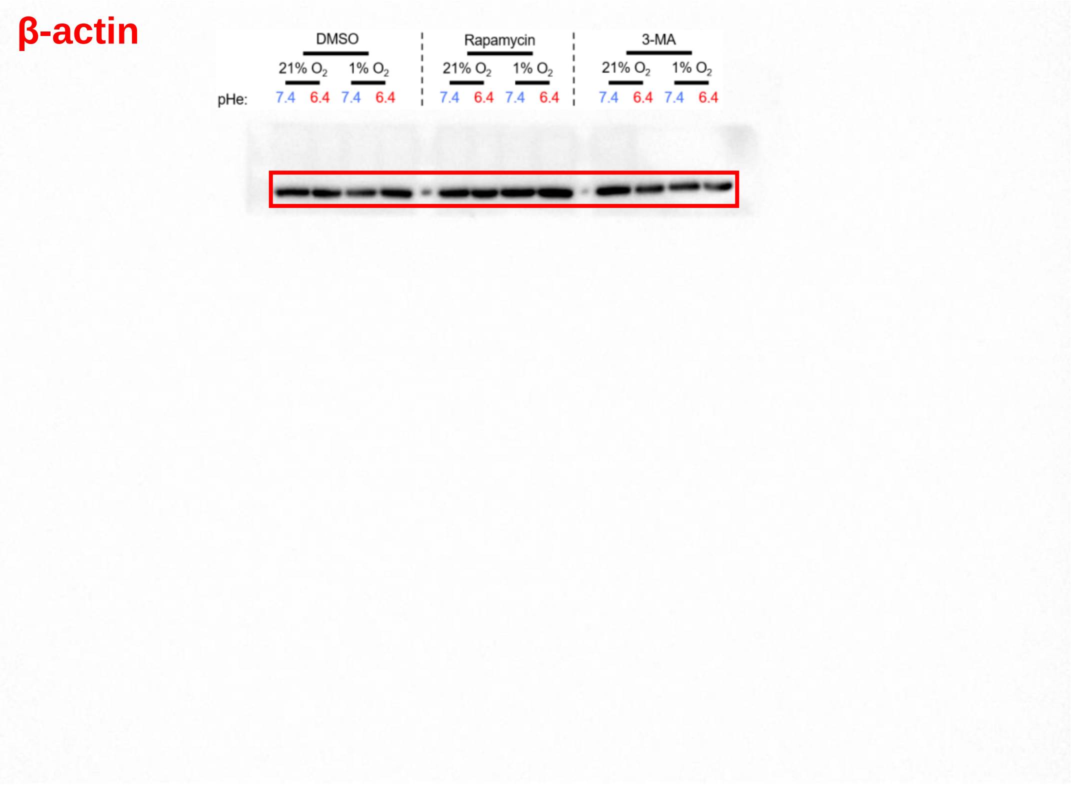
β-actin
DMSO
Rapamycin
3-MA
21% O2
1% O2
21% O2
1% O2
21% O2
1% O2
pHe:
7.4 6.4 7.4 6.4 7.4 6.4 7.4 6.4 7.4 6.4 7.4 6.4

Figure S5

C

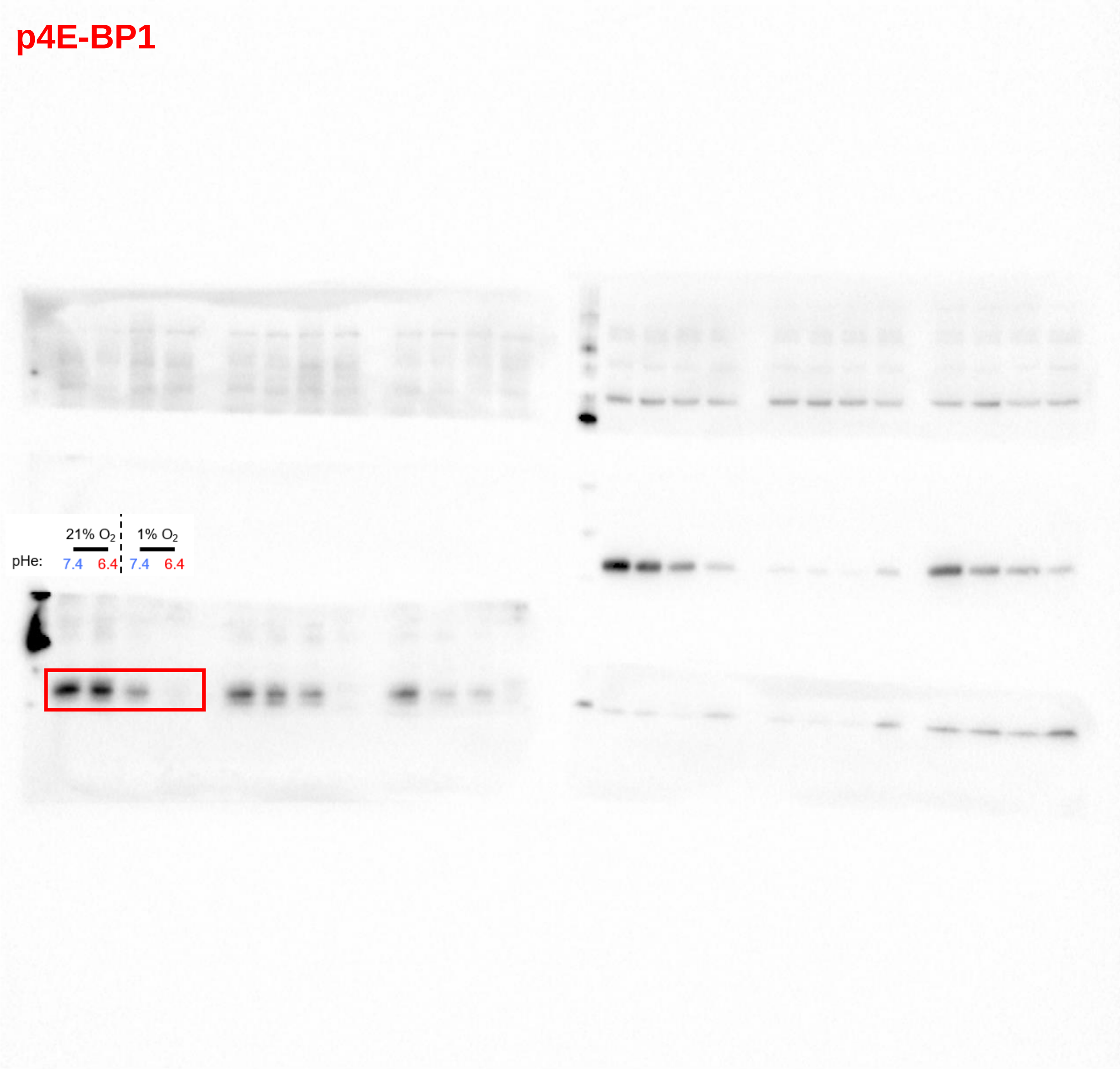

Figure S5

C

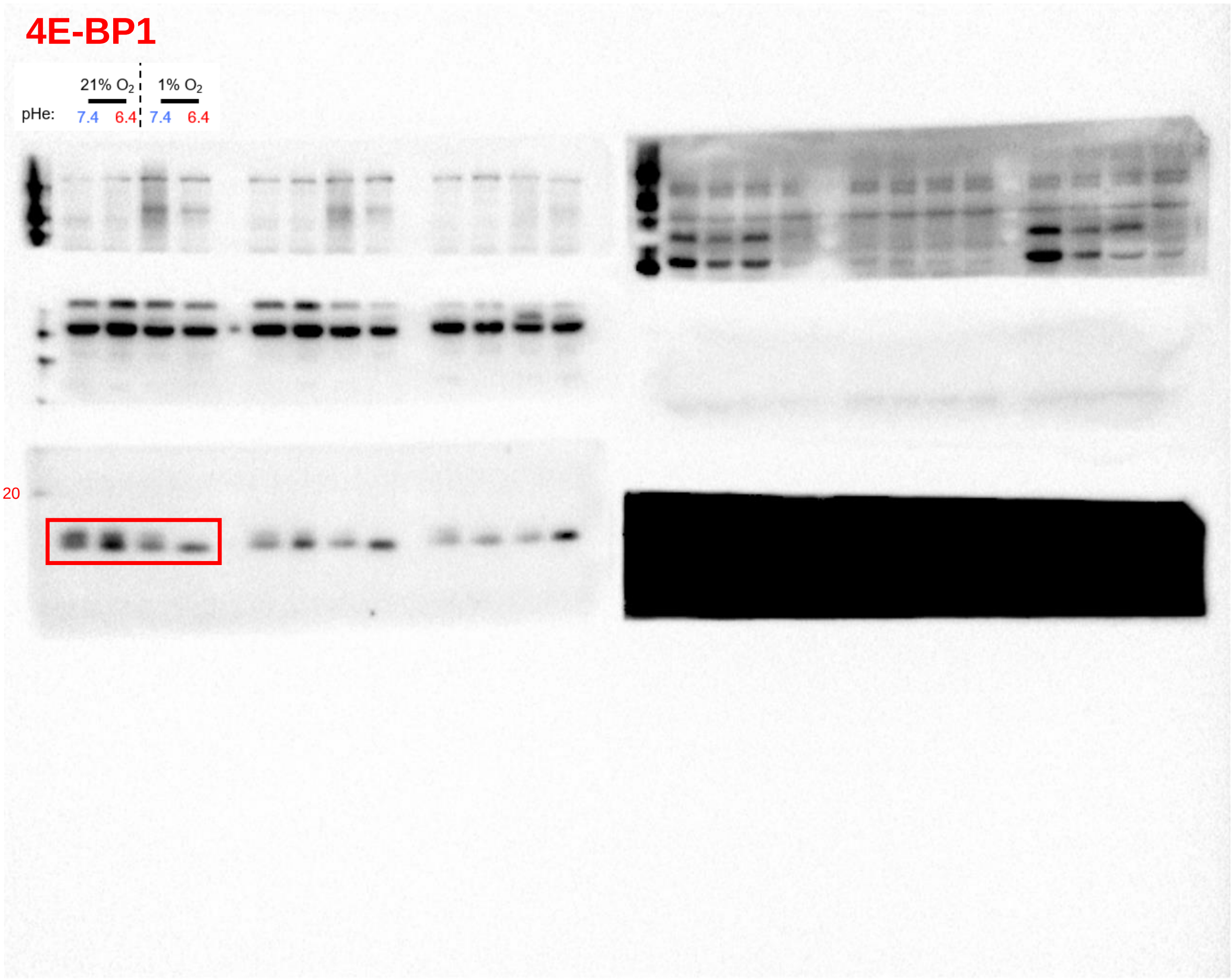

Figure S5

C

β-actin

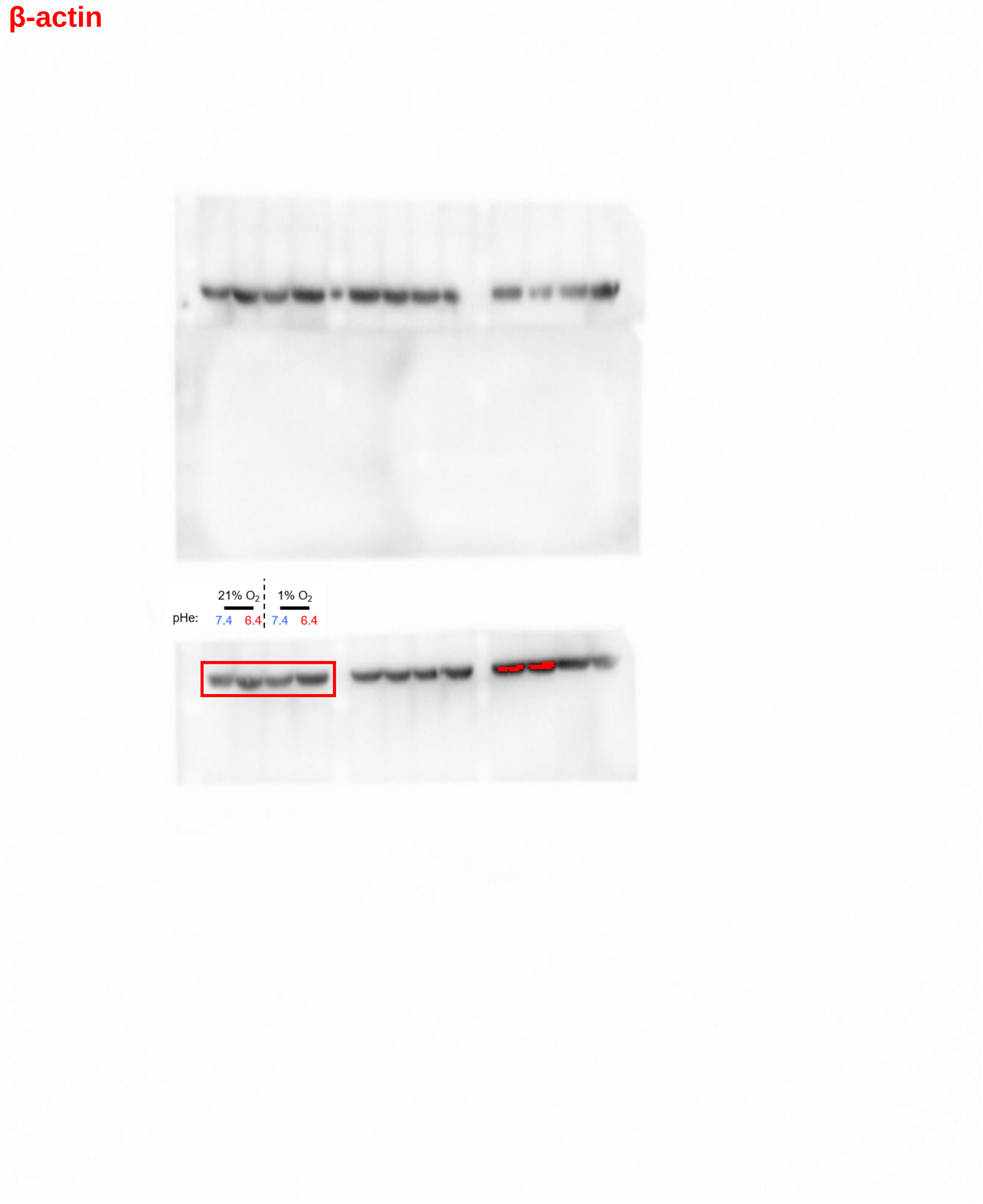

Supplement: SourceData FS5 — is the source file for Fig. S5. [file jcb_202409103_sourcedatafs5.pdf]
